# Spiral-wave dynamics in ionically realistic mathematical models for human ventricular tissue: the effects of periodic deformation

**DOI:** 10.3389/fphys.2014.00207

**Published:** 2014-06-10

**Authors:** Alok R. Nayak, Rahul Pandit

**Affiliations:** ^1^Centre for Condensed Matter Theory, Department of Physics, Indian Institute of ScienceBangalore, India; ^2^Robert Bosch Centre for Cyber Physical Systems, Indian Institute of ScienceBangalore, India; ^3^Jawaharlal Nehru Centre for Advanced Scientific ResearchBangalore, India

**Keywords:** arrhythmias, fibrillation, ventricular model, wave-dynamics, spiral turbulence, periodic deformation, low-amplitude pulses

## Abstract

We carry out an extensive numerical study of the dynamics of spiral waves of electrical activation, in the presence of periodic deformation (PD) in two-dimensional simulation domains, in the biophysically realistic mathematical models of human ventricular tissue due to (a) ten-Tusscher and Panfilov (the TP06 model) and (b) ten-Tusscher, Noble, Noble, and Panfilov (the TNNP04 model). We first consider simulations in cable-type domains, in which we calculate the conduction velocity θ and the wavelength λ of a plane wave; we show that PD leads to a periodic, spatial modulation of θ and a temporally periodic modulation of λ; both these modulations depend on the amplitude and frequency of the PD. We then examine three types of initial conditions for both TP06 and TNNP04 models and show that the imposition of PD leads to a rich variety of spatiotemporal patterns in the transmembrane potential including states with a single rotating spiral (RS) wave, a spiral-turbulence (ST) state with a single meandering spiral, an ST state with multiple broken spirals, and a state SA in which all spirals are absorbed at the boundaries of our simulation domain. We find, for both TP06 and TNNP04 models, that spiral-wave dynamics depends sensitively on the amplitude and frequency of PD and the initial condition. We examine how these different types of spiral-wave states can be eliminated in the presence of PD by the application of low-amplitude pulses by square- and rectangular-mesh suppression techniques. We suggest specific experiments that can test the results of our simulations.

## 1. Introduction

Sudden cardiac arrest is caused, in many cases, by cardiac arrhythmias, such as ventricular tachyacardia (VT) and ventricular fibrillation (VF) (Roger et al., 2011, 2012). Estimates suggest that VF is the main reason for death in 30% of the cases in which heart failure occurs (Zipes and Wellens, 1998; Fogoros, 2011). Thus, the importance of studying such arrhythmias cannot be overemphasized. Such studies must use interdisciplinary approaches because they require inputs from biology, bio-medical engineering, and cardiology, on the one hand, and physics, non-linear dynamics, and numerical methods, on the other; methods from these areas must be used to study the complicated, non-linear, partial-differential-equation models that have been developed for cardiac tissue. Such equations can show, *inter alia*, spiral-wave turbulence and spatiotemporal chaos, which is believed to be one of the mathematical analogs of VF. The study we present here combines theoretical ideas from spatiotemporal chaos in extended dynamical systems with extensive direct numerical simulations, to elucidate the effects of periodic deformation (PD) on spiral-wave dynamics in detailed mathematical models for cardiac tissue and to investigate the elimination of such spiral waves, in the presence of PD, by the application of low-amplitude current pulses.

The mechanisms underlying VT and VF are not understood with complete certainty; however, various clinical studies (Fenton et al., 2008; Fogoros, 2011) have suggested that such arrhythmias comprise the abnormal propagation of a wave of electrical activation across the ventricles. Such irregular waves appear in various ways. For example, they can arise because of an infarction scar (De Bakker et al., 1993), which can create an anatomical path that anchors electrical waves; they are also seen around obstacles *in vitro* (Valderrábano et al., 2000) and in cell-culture experiments (Lim et al., 2006) (anatomically reentry). However, reentry can occur in the absence of an anatomical pathway. For instance, *in vitro* experiments (Davidenko et al., 1990; Ikeda et al., 1996) have shown that fibrillation can be maintained by functional reentry. However, ectopic activations (Chen et al., 1999; Zimmermann and Kalusche, 2001), because of pulmonary veins or abnormal cells, can also initiate fibrillation. In particular, both experimental (Davidenko et al., 1992; Pertsov et al., 1993; Gray et al., 1998; Jalife et al., 1998) and computational (Jalife et al., 1998; Fenton et al., 2002; Cherry and Fenton, 2008) studies have suggested that VT and VF are, respectively, manifestations of (a) a rotating spiral (RS) or scroll wave or (b) broken spiral or scroll waves that lead to spiral- or scroll-wave turbulence (ST).

Several studies have investigated the transition from RS to ST, both in experiments on cardiac tissue and in computational studies of mathematical models for cardiac tissue; they find that this transition can occur because of (a) a steep, increasing initial segment in the restitution curve, a plot of the action potential duration (APD) versus the diastolic interval (DI) (Koller et al., 1998; Garfinkel et al., 2000; Fenton et al., 2002), (b) a similar steep part in an analogous plot of the conduction velocity θ versus DI (Qu et al., 2000b; Fenton et al., 2002), (c) alternans (Karma, 1994; Koller et al., 1998; Qu et al., 1999; Cherry and Fenton, 2008), and (d) heterogeneities, such as, conduction and ionic inhomogeneities (Xie et al., 1998; Shajahan et al., 2007, 2009; Majumder et al., 2011a,b). Recently, some groups (Zhang et al., 2004, 2006; Panfilov et al., 2007; Chen et al., 2008; Weise et al., 2011) have begun to study the effects of the deformation of cardiac tissue on the RS-ST transition; such a transition arises either because of periodic deformation or the stretch-activated current associated with such deformation. These studies have used simple, two-variable mathematical models for electrical activation in such tissue. One of the goals of our study is to investigate spiral-wave dynamics in general, and RS-ST transitions in particular, in a simple mathematical model for periodic deformation (PD) of cardiac tissue (Zhang et al., 2004, 2006; Chen et al., 2008) that we couple with ionically realistic human-ventricular-tissue mathematical models, namely, (a) the TP06 model, due to ten Tusscher and Panfilov (Ten Tusscher and Panfilov, 2006), or (b) the TNNP04 model, of ten Tusscher, Noble, Noble, and Panfilov (Ten Tusscher et al., 2004). A deformation of cardiac tissue leads to modifications of ion-channel parameters, because of stretch-activated currents, and also a modification of intracellular couplings. Our model for deformation, based on Zhang et al. (2004, 2006) and Chen et al. (2008), is a very simplified one in which the effects of deformation are accounted for only by a temporal modulation of diffusion constants (see section 2), which are related to intracellular couplings; we do not include stretch-activated currents as considered in Panfilov et al. (2005, 2007) and Weise et al. (2011). However, in spite of this simplified representation of deformation, our study yields important results that have been observed in two-variable models for cardiac tissue both with periodic deformation (PD) (Zhang et al., 2004, 2006; Chen et al., 2008) or mechanical deformation (Panfilov et al., 2005, 2007; Weise et al., 2011); the latter studies include stretch-activated currents. Such stretch-activated currents with mechano-electrical feedback have also been shown to affect electrical activation in anatomically realistic human models (Keldermann et al., 2010; Kuijpers et al., 2011); mechano-electrical feedback can enhance electrical activation as discussed in Thompson et al. (2011) in the context of myofibroblast-myocyte interactions (of course, pharmacological and electrochemical interventions can also enhance electrical activation). On the positive side, our study uses ionically realistic models that have not been employed in such deformation studies so far (but see the recent Weise and Panfilov, 2013). We discuss the principal results of the studies carried out in Zhang et al. (2004, 2006), Panfilov et al. (2005, 2007), Chen et al. (2008), and Weise et al. (2011), in section 4, where we compare their findings with ours.

We also investigate the efficacy of a low-amplitude suppression scheme, developed for the suppression of spiral-wave turbulence in 2D models for cardiac tissue (Sinha et al., 2001; Pandit et al., 2002; Shajahan et al., 2009; Majumder et al., 2011a) in the absence of PD. Our study yields several interesting results that we summarize below before we discuss them in detail. We find first that PD leads to a periodic, spatial modulation of the conduction velocity θ and a temporally periodic modulation of the wavelength λ of a plane wave. We then use three different parameter sets, for both TP06 and TNNP04 models, to obtain three different prototypical spiral configurations, which we use as the initial conditions *IC*1, *IC*2, and *IC*3 (see section 2). We find, for the TP06 model, that spiral-wave dynamics depends sensitively on PD and the initial condition. (For similar studies of the sensitive dependence of spiral-wave dynamics on inhomogeneities, see Shajahan et al., 2007, 2009.) The initial condition *IC*1 can lead to (a) an RS state with *n*-cycle temporal evolution (here *n* is a positive integer), (b) rotating-spiral states with quasi-periodic (QP) temporal evolution, (c) a state with a single meandering spiral MS, which displays spatiotemporal chaos, (d) an ST state, with multiple broken spirals, and (e) a quiescent state SA, in which all spirals are absorbed; the initial condition *IC*2, with PD, can lead either to (a) an ST state, with multiple spirals, or (b) an SA state, with no spirals; and for *IC*3, it can be driven into (a) an ST state, with a single meandering spiral, (b) an ST state, with multiple spirals, and (c) the state SA. For all these initial conditions, precisely which one of these states is obtained depends on the amplitudes *A*_*x*_ and *A*_*y*_ and the frequencies *f*_*x*_ and *f*_*y*_ of the PD in the *x* and *y* directions. Spiral-wave dynamics in the TNNP04 model, with PD, also shows sensitive dependence on PD and the initial condition. This sensitive dependence on parameters is a hallmark of extended dynamical systems that show spatiotemporal chaos (Shajahan et al., 2007, 2009). We also study, in the presence of PD, the efficacy of a low-amplitude suppression scheme (Sinha et al., 2001; Shajahan et al., 2009) that has been suggested, hitherto only without PD, for the suppression of spiral-wave turbulence, via low-amplitude current pulses applied on a square mesh, in mathematical models for cardiac tissue. Furthermore, we develop line-mesh and rectangular-mesh variants of this suppression scheme. The latter suppresses spiral turbulence in all cases we consider.

## 2. Methods

The electrical activation of the transmembrane potential *V*_*m*_ of cardiac tissue is often modeled by a reaction-diffusion-type equation,

(1) $$\frac{\partial V_{m}}{\partial t}+\frac{I_{ion}}{C_{m}}=D_{x}\frac{\partial^{2}V_{m}}{\partial x^{2}}+D_{y}\frac{\partial^{2}V_{m}}{\partial y^{2}},$$

where *C*_*m*_ is the membrane capacitance density, *I*_*ion*_ is the sum of all the ionic currents that cross the cell membrane, and *D*_*x*_ and *D*_*y*_ are, respectively, the *diffusion coefficients* along *x* and *y* directions; such diffusion terms are related to gap junctions (Ten Tusscher et al., 2004; Ten Tusscher and Panfilov, 2006), which are networks of protein channels that allow the passage of ions from cell to cell. We use two biophysically realistic ionic models for human cardiac myocytes: (a) the ten Tusscher and Panfilov model (the TP06 model) (Ten Tusscher and Panfilov, 2006) and (b) the ten Tusscher, Noble, Noble, and Panfilov model (the TNNP04 model) (Ten Tusscher et al., 2004). It is important to check the compatibility of the models that we consider in our study with the standard version of the original models (Ten Tusscher et al., 2004; Ten Tusscher and Panfilov, 2006). One way to check for such compatibility is by comparing the AP and its morphological properties (Figure S1 and Table S1, Supplementary Material S1) with those in our studies; we have checked this explicitly, as we discuss in detail in the Supplementary Material S1.

We follow the method suggested in Zhang et al. (2004, 2006) and Chen et al. (2008) for the introduction of PD into a mathematical model for cardiac tissue. In particular, we note that any point **x** = (*x, y*) in the medium changes to **x**′(*t*) = (*x*′(*t*), *y*′(*t*)) with

(2) $$\begin{array}{l} x^{\prime }(t)=x\left[1+A_{x}(t)\right],\\ y^{\prime }(t)=y\left[1+A_{y}(t)\right], \end{array}$$

if we impose a PD with *A*_*x*_(*t*) = *A*_*x*_ cos (2π*f*_*x*_*t*) and *A*_*y*_(*t*) = *A*_*y*_ cos (2π*f*_*y*_*t*). By substituting Equation (2) into Equation (1), we obtain

(3) $$\begin{array}{l} \frac{\partial V_{m}}{\partial t}+\frac{I_{ion}}{C_{m}}=\frac{1}{{\left[1+A_{x}(t)\right]}^{2}}D_{x}\frac{\partial^{2}V_{m}}{\partial x^{2}}\\ \;+\frac{1}{{\left[1+A_{y}(t)\right]}^{2}}D_{y}\frac{\partial^{2}V_{m}}{\partial y^{2}}; \end{array}$$

a comparison of Equations (1, 3) shows that Equation (3) can be rewritten as

(4) $$\frac{\partial V_{m}}{\partial t}+\frac{I_{ion}}{C_{m}}=D_{x}(t)\frac{\partial^{2}V_{m}}{\partial x^{2}}+D_{y}(t)\frac{\partial^{2}V_{m}}{\partial y^{2}},$$

with *D*_*x*_(*t*) = *D*_*x*_ (1 + *A*_*x*_(*t*))^−2^ and *D*_*y*_(*t*) = *D*_*y*_ (1 + *A*_*y*_(*t*))^−2^.

In our numerical simulations, we use 2D square domains with 1024 × 1024 grid points and lattice spacings δ*x* = δ*y* = 0.25 mm for both TP06 and TNNP04 models, so the sides of our square simulation domains are *L* = 256 mm in the absence of PD. We use a forward-Euler method for time evolution, with a time step δ*t* = 0.02 ms, a five-point stencil for the Laplacian, and no-flux (Neumann) boundary conditions. We set the diffusion coefficients *D*_*x*_ = *D*_*y*_ = *D* = 0.00154 cm^2^/ms (Ten Tusscher et al., 2004; Ten Tusscher and Panfilov, 2006) for both the TP06 and the TNNP04 models for our numerical investigations. Other parameters for our calculations are given in Tables 1, 2 and an examination of the numerical stability of our numerical scheme is given in the Supplementary Material S1.

**Table 1:** **Parameters for the periodic deformation (PD) that we use to study the wave dynamics in our cable-type and square simulation domains in both TP06 and TNNP04 ventricular models**.

**Type of domain**	**Dimension of domain (mm)**	**Parameter sets**	**Amplitude of PD**	**Frequency of PD (Hz)**
	*L*_*x*_ = 1024, *L*_*y*_ = 4	(a00)	*A*_*x*_ = 0, *A*_*y*_ = 0	*f*_*x*_ = 0, *f*_*y*_ = 0
	*L*_*x*_ = 1024, *L*_*y*_ = 4	(a01)	*A*_*x*_ = 0.1, *A*_*y*_ = 0	*f*_*x*_ = 1, *f*_*y*_ = 0
	*L*_*x*_ = 1024, *L*_*y*_ = 4	(a02)	*A*_*x*_ = 0.2, *A*_*y*_ = 0	*f*_*x*_ = 1, *f*_*y*_ = 0
	*L*_*x*_ = 1024, *L*_*y*_ = 4	(a03)	*A*_*x*_ = 0.3, *A*_*y*_ = 0	*f*_*x*_ = 1, *f*_*y*_ = 0
	*L*_*x*_ = 1024, *L*_*y*_ = 4	(a04)	*A*_*x*_ = 0.4, *A*_*y*_ = 0	*f*_*x*_ = 1, *f*_*y*_ = 0
	*L*_*x*_ = 1024, *L*_*y*_ = 4	(a05)	*A*_*x*_ = 0.5, *A*_*y*_ = 0	*f*_*x*_ = 1, *f*_*y*_ = 0
	*L*_*x*_ = 1024, *L*_*y*_ = 4	(a06)	*A*_*x*_ = 0.1, *A*_*y*_ = 0	*f*_*x*_ = 3, *f*_*y*_ = 0
	*L*_*x*_ = 1024, *L*_*y*_ = 4	(a07)	*A*_*x*_ = 0.2, *A*_*y*_ = 0	*f*_*x*_ = 3, *f*_*y*_ = 0
	*L*_*x*_ = 1024, *L*_*y*_ = 4	(a08)	*A*_*x*_ = 0.3, *A*_*y*_ = 0	*f*_*x*_ = 3, *f*_*y*_ = 0
	*L*_*x*_ = 1024, *L*_*y*_ = 4	(a09)	*A*_*x*_ = 0.4, *A*_*y*_ = 0	*f*_*x*_ = 3, *f*_*y*_ = 0
	*L*_*x*_ = 1024, *L*_*y*_ = 4	(a10)	*A*_*x*_ = 0.5, *A*_*y*_ = 0	*f*_*x*_ = 3, *f*_*y*_ = 0
Cable	*L*_*x*_ = 1024, *L*_*y*_ = 4	(a11)	*A*_*x*_ = 0.1, *A*_*y*_ = 0	*f*_*x*_ = 5, *f*_*y*_ = 0
	*L*_*x*_ = 1024, *L*_*y*_ = 4	(a12)	*A*_*x*_ = 0.2, *A*_*y*_ = 0	*f*_*x*_ = 5, *f*_*y*_ = 0
	*L*_*x*_ = 1024, *L*_*y*_ = 4	(a13)	*A*_*x*_ = 0.3, *A*_*y*_ = 0	*f*_*x*_ = 5, *f*_*y*_ = 0
	*L*_*x*_ = 1024, *L*_*y*_ = 4	(a14)	*A*_*x*_ = 0.4, *A*_*y*_ = 0	*f*_*x*_ = 5, *f*_*y*_ = 0
	*L*_*x*_ = 1024, *L*_*y*_ = 4	(a15)	*A*_*x*_ = 0.5, *A*_*y*_ = 0	*f*_*x*_ = 5, *f*_*y*_ = 0
	*L*_*x*_ = 1024, *L*_*y*_ = 4	(a16)	*A*_*x*_ = 0.1, *A*_*y*_ = 0	*f*_*x*_ = 7, *f*_*y*_ = 0
	*L*_*x*_ = 1024, *L*_*y*_ = 4	(a17)	*A*_*x*_ = 0.2, *A*_*y*_ = 0	*f*_*x*_ = 7, *f*_*y*_ = 0
	*L*_*x*_ = 1024, *L*_*y*_ = 4	(a18)	*A*_*x*_ = 0.3, *A*_*y*_ = 0	*f*_*x*_ = 7, *f*_*y*_ = 0
	*L*_*x*_ = 1024, *L*_*y*_ = 4	(a19)	*A*_*x*_ = 0.4, *A*_*y*_ = 0	*f*_*x*_ = 7, *f*_*y*_ = 0
	*L*_*x*_ = 1024, *L*_*y*_ = 4	(a20)	*A*_*x*_ = 0.5, *A*_*y*_ = 0	*f*_*x*_ = 7, *f*_*y*_ = 0
	*L*_*x*_ = 256, *L*_*y*_ = 256	(a1)	*A*_*x*_ = 0.1, *A*_*y*_ = 0.1	*f*_*x*_ = 1, *f*_*y*_ = 1
	*L*_*x*_ = 256, *L*_*y*_ = 256	(a2)	*A*_*x*_ = 0.2, *A*_*y*_ = 0.2	*f*_*x*_ = 1, *f*_*y*_ = 1
	*L*_*x*_ = 256, *L*_*y*_ = 256	(a3)	*A*_*x*_ = 0.3, *A*_*y*_ = 0.3	*f*_*x*_ = 1, *f*_*y*_ = 1
	*L*_*x*_ = 256, *L*_*y*_ = 256	(a4)	*A*_*x*_ = 0.4, *A*_*y*_ = 0.4	*f*_*x*_ = 1, *f*_*y*_ = 1
	*L*_*x*_ = 256, *L*_*y*_ = 256	(a5)	*A*_*x*_ = 0.5, *A*_*y*_ = 0.5	*f*_*x*_ = 1, *f*_*y*_ = 1
	*L*_*x*_ = 256, *L*_*y*_ = 256	(b1)	*A*_*x*_ = 0.1, *A*_*y*_ = 0.1	*f*_*x*_ = 3, *f*_*y*_ = 3
	*L*_*x*_ = 256, *L*_*y*_ = 256	(b2)	*A*_*x*_ = 0.2, *A*_*y*_ = 0.2	*f*_*x*_ = 3, *f*_*y*_ = 3
	*L*_*x*_ = 256, *L*_*y*_ = 256	(b3)	*A*_*x*_ = 0.3, *A*_*y*_ = 0.3	*f*_*x*_ = 3, *f*_*y*_ = 3
	*L*_*x*_ = 256, *L*_*y*_ = 256	(b4)	*A*_*x*_ = 0.4, *A*_*y*_ = 0.4	*f*_*x*_ = 3, *f*_*y*_ = 3
	*L*_*x*_ = 256, *L*_*y*_ = 256	(b5)	*A*_*x*_ = 0.5, *A*_*y*_ = 0.5	*f*_*x*_ = 3, *f*_*y*_ = 3
Tissue	*L*_*x*_ = 256, *L*_*y*_ = 256	(c1)	*A*_*x*_ = 0.1, *A*_*y*_ = 0.1	*f*_*x*_ = 5, *f*_*y*_ = 5
	*L*_*x*_ = 256, *L*_*y*_ = 256	(c2)	*A*_*x*_ = 0.2, *A*_*y*_ = 0.2	*f*_*x*_ = 5, *f*_*y*_ = 5
	*L*_*x*_ = 256, *L*_*y*_ = 256	(c3)	*A*_*x*_ = 0.3, *A*_*y*_ = 0.3	*f*_*x*_ = 5, *f*_*y*_ = 5
	*L*_*x*_ = 256, *L*_*y*_ = 256	(c4)	*A*_*x*_ = 0.4, *A*_*y*_ = 0.4	*f*_*x*_ = 5, *f*_*y*_ = 5
	*L*_*x*_ = 256, *L*_*y*_ = 256	(c5)	*A*_*x*_ = 0.5, *A*_*y*_ = 0.5	*f*_*x*_ = 5, *f*_*y*_ = 5
	*L*_*x*_ = 256, *L*_*y*_ = 256	(d1)	*A*_*x*_ = 0.1, *A*_*y*_ = 0.1	*f*_*x*_ = 7, *f*_*y*_ = 7
	*L*_*x*_ = 256, *L*_*y*_ = 256	(d2)	*A*_*x*_ = 0.2, *A*_*y*_ = 0.2	*f*_*x*_ = 7, *f*_*y*_ = 7
	*L*_*x*_ = 256, *L*_*y*_ = 256	(d3)	*A*_*x*_ = 0.3, *A*_*y*_ = 0.3	*f*_*x*_ = 7, *f*_*y*_ = 7
	*L*_*x*_ = 256, *L*_*y*_ = 256	(d4)	*A*_*x*_ = 0.4, *A*_*y*_ = 0.4	*f*_*x*_ = 7, *f*_*y*_ = 7
	*L*_*x*_ = 256, *L*_*y*_ = 256	(d5)	*A*_*x*_ = 0.5, *A*_*y*_ = 0.5	*f*_*x*_ = 7, *f*_*y*_ = 7

**Table 2:** **Parameters for the initial spiral-wave configurations for TP06 and TNNP04 models; we refer to these as *IC*1, *IC*2, and *IC*3 initial conditions**.

**Model**	**Initial condition**	***G*_*Na*_ (*nS/pF*)**	***G*_*kr*_ (*nS/pF*)**	***G*_*ks*_ (*nS/pF*)**	***G*_*pCa*_ (*nS/pF*)**	***G*_*pK*_ (*nS/pF*)**	**σ_*f*_**
TP06	*IC*1	14.838	0.153	0.392	0.1238	0.0146	1
TP06	*IC*2	5 × 14.838	0.153	0.392	0.1238	0.0146	1
TP06	*IC*3	14.838	0.172	0.441	0.8666	0.00219	2
TNNP04	*IC*1	14.838	0.096	0.245	0.825	0.0146	1
TNNP04	*IC*2	5 × 14.838	0.096	0.245	0.825	0.0146	1
TNNP04	*IC*3	3 × 14.838	0.096	0.245	5 × 0.825	0.0146	2

*Here, σ_f_ is the scale factor of the time constant τ_f_ (Ten Tusscher et al., 2004; Ten Tusscher and Panfilov, 2006)*.

To examine the spatiotemporal evolution of the electrical signal of the transmembrane potential, we obtain the local time series of *V*_*m*_(*x, y, t*), from a representative point (*x* = 125 mm, *y* = 125 mm) (shown by an asterisk in all pseudocolor plots of *V*_*m*_). To obtain the plots of the inter-beat interval (IBI), we use this local time series with 4 × 10^5^ data points; the IBI is the time interval between two successive beats in the time series of the signal *V*_*m*_. For the power spectra *E*(ω) we use the local time series with 2 × 10^5^ data points after the initial 10^5^ data points have been removed to eliminate transients. We present the spatiotemporal evolution of *V*_*m*_ by a series of (Videos S1–S9) of its pseudocolor plots; all these videos use 10 frames per second and each frame is separated from the succeeding frame by 8 ms.

We often have to track the trajectory of the tip of a spiral wave in a 2D simulation domain. The tip of such a spiral wave is normally defined as the point where the excitation wave front and repolarization wave back meet; this point can be found by a variety of methods (Barkley et al., 1990; Fenton and Karma, 1998; Fenton et al., 2002; Otani, 2002; Gray et al., 2009; Nayak et al., 2013). We use the tip-tracking algorithm of Nayak et al. (2013) that locates the tip position by monitoring *I*_*Na*_, the sodium current. Pseudocolor plots of *I*_*Na*_ show a fine line along the arm of a spiral wave (Figure 2A in Shajahan et al., 2009); this line terminates in the spiral tip and can, therefore, be used to obtain the spatiotemporal evolution of this tip.

In Figures S2A–F in the Supplementary Material S1, we show schematic diagrams for illustrative periodic deformations of a small part of our simulation domain, with 5 × 5 grid points (indicated by gpts on the axes of figures); in these diagrams, blue, open circles and blue, dashed lines show, at a particular instant of time, the deformed simulation domain superimposed on the undeformed one, which is represented by black, solid circles and black, full lines. We give representative diagrams for the case of expansion, with deformations along only *x*, only *y*, or both *x* and *y* directions, in Figures S2A–C, in the Supplementary Material S1, at time *t* = 20 ms; the corresponding plots for contraction, at time *t* = 180 ms, are shown in Figures S2D–F, in the Supplementary Material S1.

## 3. Results

We begin by exploring the effects of PD both on plane-wave propagation and on spiral-wave dynamics; here we vary the oscillation amplitude and the frequency in the ranges 0 ≲ *A*_*x*_, *A*_*y*_ ≲ 0.5 and 0 Hz ≲ *f* ≲ 7.0 Hz; the deformation amplitudes we use are comparable to those in other computational (Zhang et al., 2004, 2006; Weise et al., 2011) and experimental (McCULLOCH et al., 1987; Noble, 2002) studies; to set the scale of frequencies, we note that the frequency of rotation of a single spiral wave is 4.75 Hz for the TP06 model and 3.75 Hz for the TNNP04 model (see section 3.2). We then study the effects of PD on the suppression scheme of Pandit et al. (2002) and Shajahan et al. (2009).

### 3.1. Plane-wave dynamics in a cable

We study plane-wave propagation in a thin, cable-type simulation domain, with 16 × 4096 grid points, i.e., *L*_*x*_ = 4 mm and *L*_*y*_ = 1024 mm. We inject a stimulus of strength *I*_*stimulus*_ = 150 pA/pF at the left end of the cable for 3 ms and then study the effects of PD on the plane wave that propagates through this cable; in particular, we measure the conduction velocity θ and wavelength λ of the propagating wave in the cable. We find that θ ≃ 70.6 cm/s and λ ≃ 21.6 cm for the TP06 model, and θ ≃ 67.8 cm/s and λ ≃ 18.9 cm for the TNNP04 model, in the absence of PD. As suggested in Clayton and Panfilov (2008), Shajahan et al. (2009), and Ten Tusscher et al. (2004), it is useful to test the accuracy of the numerical scheme by varying both the time and space steps that we use for integration. We illustrate this for the TP06 model by measuring θ for a plane wave, which is injected into the medium by stimulating the left boundary of our simulation domain. We find that, with δ*x* = 0.025 cm, θ increases by 1.6% as we decrease δ*t* from 0.02 to 0.01 ms; if we use δ*t* = 0.02 ms and decrease δ*x* from 0.025 to 0.015 cm then θ increases by 4.7%; such changes are comparable to those found in earlier studies (Ten Tusscher et al., 2004; Shajahan et al., 2009).

**Figure 1:**
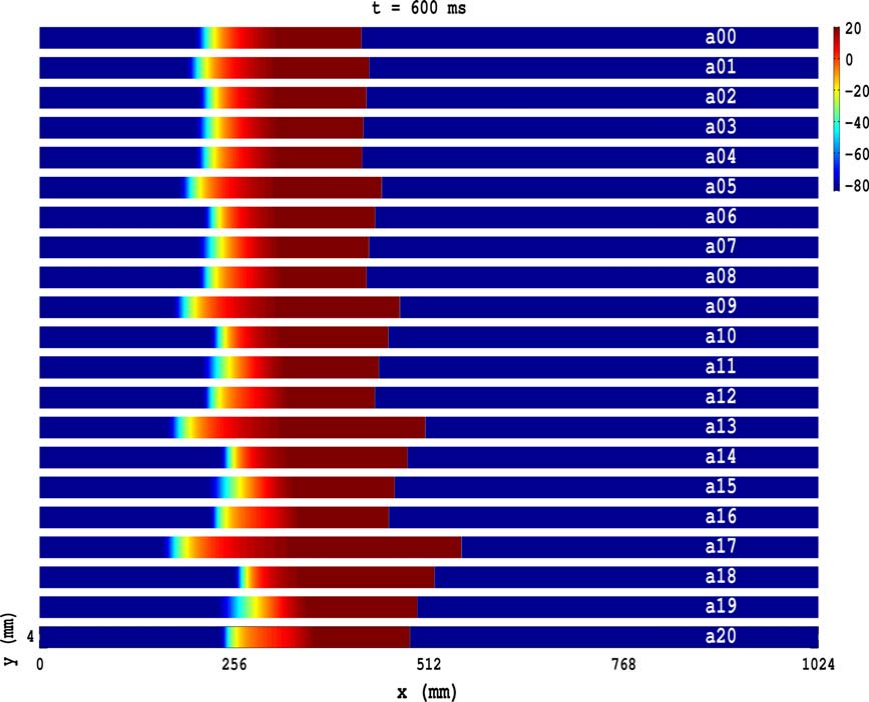
**Pseudocolor plots of the transmembrane potential *V*_*m*_ for the TP06 model illustrating plane-wave propagation in a cable-type domain, with PD along the axial-direction of the cable, and the parameter sets given in Table 1**. The Video S1 comprises 21 animations that show the spatiotemporal evolution of these plane waves.

**Figure 2:**
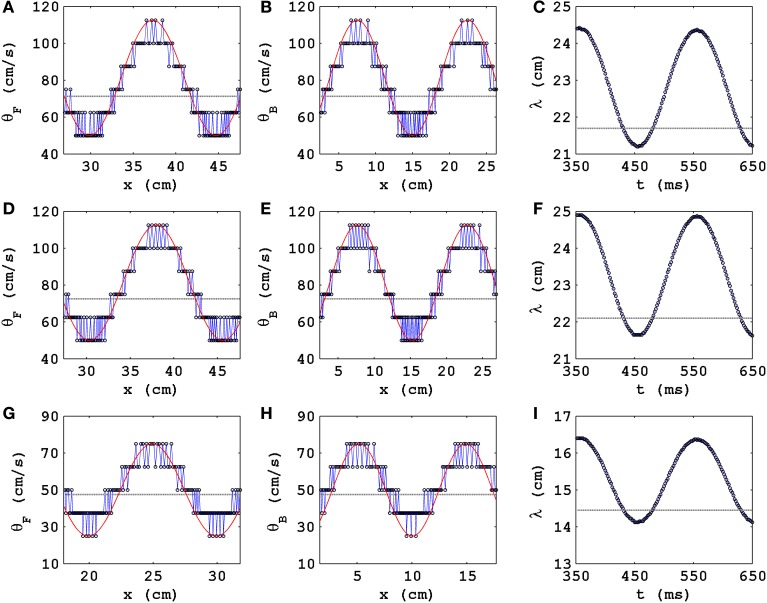
**The spatial modulation of θ and the temporal modulation of λ of a plane wave propagating in a cable-type domain with PD**. Plots versus distance *x* along the cable of the conduction velocities with *D* = 0.00154 cm^2^/ms, δ*t* = 0.02 ms, and δ*x* = 0.25 mm **(A)** θ_*F*_, of the wave front, and **(B)** θ_*B*_, of the wave back; **(C)** plot versus time *t* of the wavelength λ. The exact analogs of **(A–C)** are shown in **(D–F)**, for *D* = 0.00154 cm^2^/ms, δ*t* = 0.01 ms, and δ*x* = 0.25 mm, and in **(G–I)**, for *D* = 0.00077 cm^2^/ms, δ*t* = 0.02 ms, and δ*x* = 0.25 mm. We use the representative PD parameter values *A*_*x*_ = 0.3 and *f*_*x*_ = 5.0 Hz for the TP06 model. Open circles show the values from our calculation; the red lines show smooth sinusoidal envelopes; in the absence of PD, θ ≃ 70.6 cm/s, ≃ 71.7 cm/s, and ≃ 47 cm/s, respectively, for above three parameter sets [gray, dashed lines in **(A,B)**]; in **(C)** the gray, dashed line shows the value of λ that we obtain in the absence of PD.

In Figure 1(a00–a20) we show, at time *t* = 600 ms, when PD is applied along the axial direction of the cable, pseudocolor plots of the transmembrane potential *V*_*m*_ for the TP06 model with PD along the axial direction of the cable, and the parameter sets given in Table 1. The Video S1 comprises 21 animations that show the spatiotemporal evolution of the plane waves in Figure 1(a00–a20); these animations and Figure 1(a00–a20) show that the conduction velocity θ is modulated in space and the wavelength λ is modulated in time because of the PD. Figure 2 illustrates these modulations via plots of θ_*F*_ and θ_*B*_ versus *x* for the conduction velocities of the wave front (Figure 2A) and the wave back (Figure 2B), respectively; here the subscripts *F* and *B* stand for wave front and wave back, respectively; and Figure 2C shows the corresponding plot for λ versus time *t*; in these plots we use the representative PD parameter values *A*_*x*_ = 0.3 and *f*_*x*_ = 5.0 Hz for the TP06 model. We calculate the conduction velocities θ_*F*_(*x*) and θ_*B*_(*x*), in the cable-type domain with PD, by recording the positions of the wave front and the wave back at times *t* and *t* + Δ*t*, with Δ*t* = 2 ms; the wave-front and wave-back conduction velocities, at the point *x* at time *t*, are θ_*F*_(*x*) = Δ_*F*_*x*/Δ*t* and θ_*B*_(*x*) = Δ_*B*_*x*/Δ*t*, where Δ_*F*_*x* and Δ_*B*_*x* are, respectively, the distances traveled by the wave front and wave back in the time interval Δ*t*. We locate the position of the wave front by finding the value of *x* at which *V*_*m*_ ≃ 0 mV; we define the position of the wave back as the point, behind the wave front, at which a secondary action potential can just be initiated by an additional stimulus (this turns out to occur at a value of *V*_*m*_ that is ≃ 75% of the repolarization phase of the action potential). We obtain the wavelength λ(*t*) by measuring the distance between the wave front and the wave back at time *t*.

In Figures 2A,B, the open circles show the values of θ_*F*_(*x*) and θ_*B*_(*x*), respectively, that we obtain by the method described above; the red lines show smooth sinusoidal envelopes, with amplitude ≃ 31.2 cm/s and spatial period ≃ 14.5 cm, that give the average modulations of these conduction velocities with *x*. Note that, in the absence of PD, θ ≃ 70.6 cm/s (this is shown via a gray, dashed line in Figures 2A,B); therefore, the electrical wave can travel ≃ 70.6/*f* cm in 1/*f* s; hence, for a given PD frequency *f*, the spatial period of oscillation of θ_*F*_(*x*) and θ_*B*_(*x*) is ≃ 70.6/*f* cm; the representative plots of Figures 2A,B, in which *f* = 5 Hz and the period is ≃ 70.6/5 = 14.12 cm, are consistent with this estimate.

Figure 2C shows that λ is a periodic function of *t* with a period τ; we expect that τ = 1/*f*, where *f* is the PD frequency; the illustrative plot in Figure 2C, with *f* = 5 Hz, is consistent with this expectation because τ ≃ 202 ms; the gray, dashed line shows the value of λ that we obtain in the absence of PD.

It is useful to study how θ and λ of a plane wave behave, in the presence of PD, when we change the values of the time step and the diffusion coefficients. We find that, in the presence of PD, θ and λ continue to oscillate, as in Figures 2D–I, as functions of *x* and *t*, respectively; the mean values of θ and λ, about which these oscillations occur, are close to their values without PD, which depend on the diffusion coefficients and marginally on the time step: In Figures 2D,E we show, for *D* = 0.00154 cm^2^/ms, δ*t* = 0.01 ms, and δ*x* = 0.25 mm, the analogs of Figures 2A–C; and in Figures 2G–I we give their counterparts for *D* = 0.00077 cm^2^/ms, δ*t* = 0.01 ms, and δ*x* = 0.25 mm.

The TNNP04-model analogs of the TP06-model Figure 1(a00–a20) are given in Figure S4(a00–a11) in the Supplementary Material S1. Our results for the TNNP04 model are similar to those for the TP06 model.

### 3.2. Spiral-wave dynamics in a homogeneous domain

We move now to systematic studies of spiral-wave dynamics in a 2D, square simulation domain with side *L* = 256 mm, in the presence of PD, for both TP06 and TNNP04 models.

In the absence of PD, two methods are used to initiate spiral waves in simulations (Pertsov et al., 1993; Bernus et al., 2002; Ten Tusscher et al., 2004; Shajahan et al., 2009) and experiments (Davidenko et al., 1992; Pertsov et al., 1993), namely, (1) the S1, S2 cross-field protocol and (2) the S1, S2 parallel-field protocol. We describe in the Supplementary Material S1 the precise S1, S2 cross-field protocol that we use to obtain spiral waves in our simulation domain. We use three types of spiral-wave initial configurations for our subsequent studies; we refer to these as *IC*1, *IC*2, and *IC*3 initial conditions (see Table 2 for parameter values). In Figures S5A–C, in the Supplementary Material S1, we show the time evolution of pseudocolor plots of *V*_*m*_ for the TP06 model with the *IC*1 initial configuration; similar plots are shown in Figures S5D–E and (F,I), respectively, for the *IC*2 and *IC*3 initial configurations. The TNNP04-model analogs of Figures S5A–I are given in Figures S6A–I, in the Supplementary Material S1.

**Figure 3:**
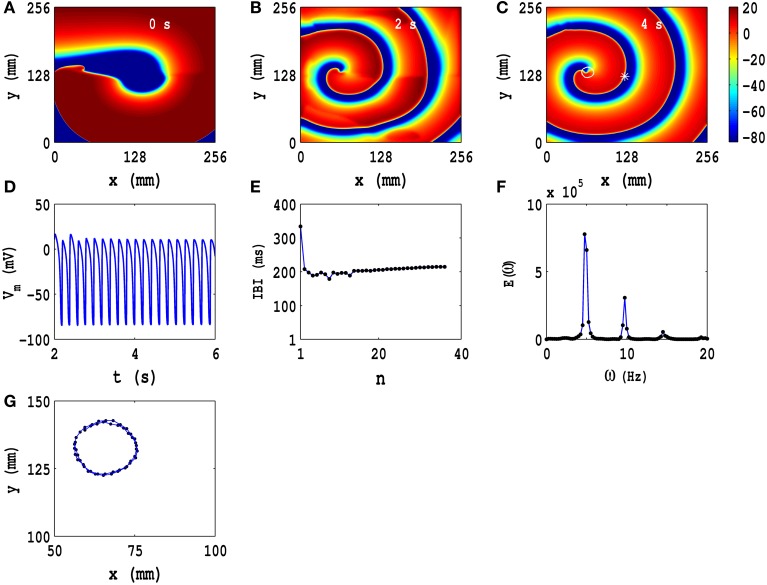
**Spatiotemporal evolution of *V*_*m*_ for the initial condition *IC*1 for the TP06 model in the absence of PD. (A–C)** Pseudocolor plots of *V*_*m*_ at times *t* = 0 s, *t* = 2 s, and *t* = 4 s, respectively, showing the evolution toward a state with a rotating spiral (RS); the animation **(A)** in Video S2 shows the spatiotemporal evolution of *V*_*m*_ for this case. **(D)** The local time series of *V*_*m*_(*x, y, t*), from the representative point (*x* = 125 mm, *y* = 125 mm) (the asterisk in **(C)**) for 2 s ≤ *t* ≤ 6 s; **(E)** a plot of the IBI, which we obtain from this time series, of length 4 × 10^5^ iterations; **(F)** the power spectrum *E*(ω), obtained from the local time series of **(D)**, with discrete peaks at the fundamental frequency ω_*f*_ ≃ 4.75 Hz and its harmonics. The spiral-tip trajectory traces a roughly circular path, with radius *l*_*c*_ ≃ 20 mm, which is shown, for 3.6 s ≤ *t* ≤ 4 s, by the white line that has been superimposed on the pseudocolor plot of *V*_*m*_ in **(C)**; a magnified view of this path is shown in **(G)**.

In Figures 3A–C, we show pseudocolor plots of *V*_*m*_ at times *t* = 0 s, *t* = 2 s, and *t* = 4 s, respectively, for the initial condition *IC*1 in the TP06 model, in the absence of PD; this initial configuration evolves to a state with a rotating spiral (RS) in the medium; the animation (a) in Video S2 shows the spatiotemporal evolution of *V*_*m*_ for this case. The local time series of *V*_*m*_(*x, y, t*), from the representative point (*x* = 125 mm, *y* = 125 mm) (the asterisk in Figure 3C), is shown in Figure 3D for 2 s ≤ *t* ≤ 6 s; a plot of the IBI is given in Figure 3E, which shows that, after initial transients (roughly the first 10 beats), the spiral wave rotates periodically with an average rotation period *T* ≃ 210 ms. In Figure 3F, we plot the power spectrum *E*(ω), which we have obtained from the local time series of *V*_*m*_ mentioned above; discrete peaks in *E*(ω) appear at the fundamental frequency ω_*f*_ ≃ 4.75 Hz and its harmonics. The periodic nature of the local time series of *V*_*m*_, the flattening of the IBI, and the discrete peaks in *E*(ω) show that the temporal evolution of the spiral wave is periodic; therefore, the spiral-tip trajectory traces a roughly circular path with radius *l*_*c*_ ≃ 20 mm; this circular path is shown, for 3.6 s ≤ *t* ≤ 4 s, by the white line that has been superimposed on the pseudocolor plot of *V*_*m*_ in Figure 3C; an expanded version of this path is shown in Figure 3G. The Figures S7, S8 in the Supplementary Material S1 show the analogs of Figure 3 for initial conditions *IC*2 and *IC*3, respectively; and the animations (b) and (c) in Video S2 show the spatiotemporal evolution of *V*_*m*_ for these cases. These animations, the pseudocolor plots of *V*_*m*_, the representative local time series of *V*_*m*_, the plots of the IBI, and the power spectra show that the initial conditions *IC*2 and *IC*3 lead, respectively, to spatiotemporal chaos and spiral turbulence (ST), with a single spiral meandering chaotically, and broken spirals, respectively, in the simulation domain.

Figures S9A–G, S10A–G, and S11A–F (Supplementary Material S1) show, respectively, the TNNP04 analogs of the TP06 Figures 3A–G (for *IC*1), Figures S7A–G in the Supplementary Material S1 (for *IC*2), and Figures S8A–F in the Supplementary Material S1 (for *IC*3); the spatiotemporal evolution of *V*_*m*_ for these three initial conditions for the TNNP04 model are given in animations (d), (e), and (f) in Video S2. From these animations and the Figures S9A–G, S10A–G, S11A–F (Supplementary Material S1) we conclude that the spatiotemporal evolution of *V*_*m*_ in the TNNP04 model, without PD, is similar to, but not identically the same as, that in the TP06 model for the initial conditions *IC*1, *IC*2, and *IC*3. One difference is that, in the TNNP04 model, we have a *Z*-type, spiral-tip trajectory in Figures S10C,G (Supplementary Material S1), whereas, for the same initial condition, we have an open spiral-tip trajectory (Figures S7C,G in the Supplementary Material S1) in the TP06 model. This shows that spiral-wave dynamics in these two models, without PD, depends sensitively on the ionic details of these models *and* on the initial conditions.

### 3.3. Spiral waves with PD

We present systematic studies of spiral-wave dynamics here by using *IC*1, *IC*2, and *IC*3 initial configurations in the presence of PD. In the TP06 model, these initial configurations lead, respectively, to (a) an RS state with a roughly circular spiral-tip trajectory, (b) a single meandering spiral with turbulence (we refer to this as SMST henceforth), and (c) multiple-spiral turbulence (MST) with broken spiral waves in the absence of PD, as we have described above. For the TNNP04 model the analogs of these states are (a) RSC, a state with a rotating spiral whose tip trajectory is roughly circular, (b) RSZ, a state with a rotating spiral whose tip trajectory is roughly *Z*-type, and (c) an MST state.

We first consider the time evolution of *IC*1 for the TP06 model in the presence of PD, for which we deform the medium periodically along both *x* and *y* directions, with amplitudes and frequencies in the ranges 0.1 ≤ *A*_*x*_ = *A*_*y*_ ≤ 0.5 and 1.0 Hz ≤ *f*_*x*_ = *f*_*y*_ ≤ 7.0 Hz, respectively.

**Figure 4:**
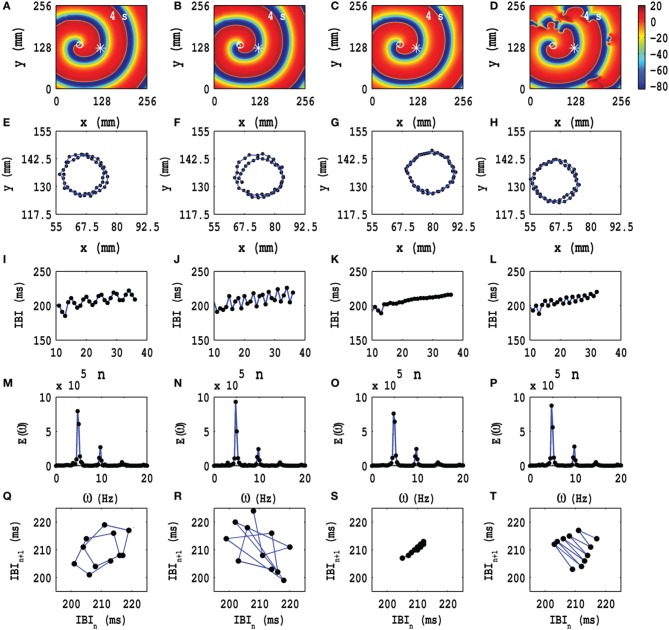
**Time evolution of the RS state in the TP06 model in the presence of PD with a fixed amplitude**. Pseudocolor plots of *V*_*m*_ at time *t* = 4 s for **(A)** *A*_*x*_ = *A*_*y*_ = 0.1, *f*_*x*_ = *f*_*y*_ = 1.0 Hz, **(B)** *A*_*x*_ = *A*_*y*_ = 0.1, *f*_*x*_ = *f*_*y*_ = 3.0 Hz, **(C)** *A*_*x*_ = *A*_*y*_ = 0.1, *f*_*x*_ = *f*_*y*_ = 5.0 Hz, and **(D)** *A*_*x*_ = *A*_*y*_ = 0.1, *f*_*x*_ = *f*_*y*_ = 7.0 Hz, respectively; the animations (a1), (b1), (c1), and (d1) in Video S3 show the spatiotemporal evolution of *V*_*m*_ for these cases in the time interval 0 s ≤ *t* ≤ 4 s. **(E–H)** Spiral-tip trajectories, which follow from these spatiotemporal evolutions, for 3.6 s ≤ *t* ≤4 s [in **(H)** we give the tip trajectory for the main, central spiral **(D)**]. We obtain the local time series of *V*_*m*_(*x, y, t*), from the representative point (*x* = 125 mm, *y* = 125 mm) [the asterisks in **(A–D)**], and therefrom the plots of the IBI **(I–L)** and the power spectra **(M–P)**. The discrete peaks in *E*(ω) appear at the following frequencies: **(M)** ω_1_ = 4.75 Hz, ω_2_ = 9.5 Hz, ω_2_ = 14.25 Hz, **(N)** ω_1_ = 4.75 Hz, ω_2_ = 9.5 Hz, ω_2_ = 14.25 Hz, and small peaks at ω_1_ = 3 Hz, ω_2_ = 7.75 Hz, ω_3_ = 11 Hz, ω_4_ = 12.5 Hz, ω_5_ = 15.75 Hz, **(O)** ω_1_ = 4.75 Hz, ω_2_ = 9.5 Hz, ω_2_ = 14.25 Hz, and **(P)** ω_1_ = 4.75 Hz, ω_2_ = 9.5 Hz, ω_2_ = 14.25 Hz. In **(I–L)** we see that the IBI shows a slight upward trend; this implies that, although the temporal evolution is nearly periodic, there is a slight drift, toward lower frequencies, in the rotation rate of the dominant spiral; note also the mild oscillations in the IBI in **(I)** a 5-cycle, **(J)** a 3-cycle, and **(L)** a 2-cycle, but not in **(K)** a 1-cycle; the natures of these oscillations and their cycle lengths are confirmed by the Poincaré-type return maps, shown in **(Q–T)**, respectively; in these return maps, successive points are connected by lines.

In Figures 4A–D we show pseudocolor plots of *V*_*m*_ at time *t* = 4 s for (a) *A*_*x*_ = *A*_*y*_ = 0.1, *f*_*x*_ = *f*_*y*_ = 1.0 Hz, (b) *A*_*x*_ = *A*_*y*_ = 0.1, *f*_*x*_ = *f*_*y*_ = 3.0 Hz, (c) *A*_*x*_ = *A*_*y*_ = 0.1, *f*_*x*_ = *f*_*y*_ = 5.0 Hz, and (d) *A*_*x*_ = *A*_*y*_ = 0.1, *f*_*x*_ = *f*_*y*_ = 7.0 Hz, respectively. The RS state, which we obtain in the absence of PD, does not evolve into an MST state in cases (a), (b) and (c); however, in case (d) the spiral arm splits into multiple spirals to yield an MST state with mild spatiotemporal chaos, in so far as the dominant spiral does not break down but continues to evolve somewhat like a mother rotor (Samie and Jalife, 2001; Chen et al., 2003; Wu et al., 2004; Ideker and Rogers, 2006); the animations (a1), (b1), (c1), and (d1) in Video S3 show the spatiotemporal evolution of *V*_*m*_ for these cases in the time interval 0 s ≤ *t* ≤ 4 s; this video uses 10 frames per second (fps) and each pseudocolor plot of *V*_*m*_ is separated from its predecessor by 8 ms. The spiral-tip trajectories, which follow from this spatiotemporal evolution, are shown in Figures 3E–H for 3.6 s ≤ *t* ≤4 s (in Figure 4H we give the tip trajectory for the main, central spiral in Figures 4A–D); these tip trajectories are nearly circular with radii *l*_*c*_ ≃ 18 mm, but, as we show below, the temporal evolution of *V*_*m*_ is different in these cases. To examine this evolution, we obtain the local time series of *V*_*m*_(*x, y, t*), from the representative point (*x* = 125 mm, *y* = 125 mm) (the asterisks in Figures 4A–D), and therefrom the plots of the IBI shown in Figures 4I–L and the power spectra of Figures 4M–P. Discrete peaks in *E*(ω) appear at the fundamental frequency ω_*f*_ ≃ 4.75 Hz and a few other frequencies (see the caption of Figure 4). From Figures 4I–L we see that the IBI displays a slight upward trend; this implies that, although the temporal evolution is nearly periodic, there is a gentle drift, toward lower frequencies, in the rotation rate of the dominant spiral. Furthermore, there are small oscillations in the IBI in Figure 4I (a 5-cycle), (j)(a 3-cycle), and (l)(a 2-cycle), but not in Figure 4K (a 1-cycle); the natures of these oscillations and their cycle lengths are confirmed by the Poincaré-type return maps, shown in Figures 4Q–T and corresponding to the IBI plots in Figures 4I–L, respectively; in these return maps, successive points are connected by lines.

**Figure 5:**
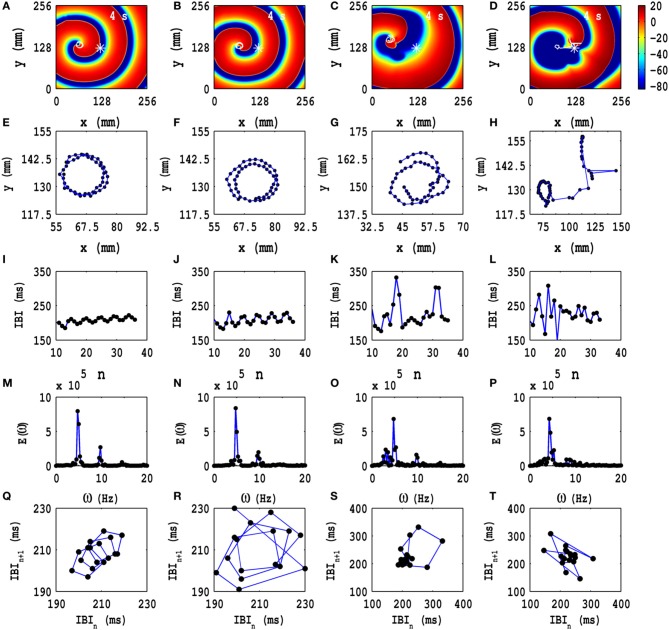
**Time evolution of the RS state in the TP06 model in the presence of PD with a fixed frequency**. Pseudocolor plots of *V*_*m*_ at time *t* = 4 s for **(A)** *A*_*x*_ = *A*_*y*_ = 0.1, *f*_*x*_ = *f*_*y*_ = 1.0 Hz, **(B)** *A*_*x*_ = *A*_*y*_ = 0.2, *f*_*x*_ = *f*_*y*_ = 1.0 Hz, **(C)** *A*_*x*_ = *A*_*y*_ = 0.3, *f*_*x*_ = *f*_*y*_ = 1.0 Hz, and **(D)** *A*_*x*_ = *A*_*y*_ = 0.4, *f*_*x*_ = *f*_*y*_ = 1.0 Hz. The animations (a1–a4) in Video S3 show the spatiotemporal evolution of these spiral waves for the interval 0 s ≤ *t* ≤ 4 s. **(E–H)** Spiral-tip trajectories, which follow from these spatiotemporal evolutions, for 3.6 s ≤ *t* ≤4 s. We obtain the local time series of *V*_*m*_(*x, y, t*), from the representative point (*x* = 125 mm, *y* = 125 mm) (the asterisks in Figures 4A–D) and therefrom the plots of the IBI shown in **(I–L)**, the power spectra *E*(ω) in **(M–P)**, and the Poincaré-type return maps, which we obtain from the IBI plots and which show **(Q)** a 5-cycle, **(R)** a 5-cycle, **(S)** chaotic behavior, and **(T)** chaotic evolution; discrete peaks in *E*(ω) appear at the following frequencies: **(M)** ω_1_ = 4.75 Hz, ω_2_ = 9.5 Hz, ω_2_ = 14.25 Hz, **(N)** ω_1_ = 4.75 Hz, ω_2_ = 9.5 Hz, ω_2_ = 14.25 Hz and small peaks at ω_1_ = 3.75 Hz, ω_2_ = 8.5 Hz, ω_3_ = 13.25 Hz, ω_4_ = 15 Hz, ω_5_ = 17.75 Hz, **(O)** ω_1_ = 4.75 Hz, ω_2_ = 9.5 Hz, and **(P)** ω_1_ = 4.5 Hz, ω_2_ = 9.5 Hz.

Similarly, we study the dependence of spiral-wave dynamics on the amplitudes *A*_*x*_ and *A*_*y*_ of the PD, with the frequencies *f*_*x*_ = *f*_*y*_ held at a fixed value. In Figure 5 we show the pseudocolor plots of *V*_*m*_ at time *t* = 4 s for (a) *A*_*x*_ = *A*_*y*_ = 0.1, *f*_*x*_ = *f*_*y*_ = 1.0 Hz, (b) *A*_*x*_ = *A*_*y*_ = 0.2, *f*_*x*_ = *f*_*y*_ = 1.0 Hz, (c) *A*_*x*_ = *A*_*y*_ = 0.3, *f*_*x*_ = *f*_*y*_ = 1.0 Hz, and (d) *A*_*x*_ = *A*_*y*_ = 0.4, *f*_*x*_ = *f*_*y*_ = 1.0 Hz. The spiral wave does not split into multiple spirals for these representative values of the amplitudes and frequencies. The animations (a1), (a2), (a3), and (a4) in Video S3 show the spatiotemporal evolution of these spiral waves for the interval 0 s ≤ *t* ≤ 4 s. To examine this evolution, we obtain the local time series of *V*_*m*_(*x, y, t*), from the representative point (*x* = 125 mm, *y* = 125 mm) (the asterisks in Figures 5A–D) and the corresponding tip trajectories of spiral waves, in the time interval 3.6 s ≤ *t* ≤ 4 s (blue lines with black points in Figures 5E,H, respectively); these tip trajectories trace nearly circular paths, with radii *l*_*c*_ ≃ 18 mm in Figures 5E,F; they are of the meandering type in Figures 5G,H, with linear extents *l*_*c*_ ≃ 24 mm and *l*_*c*_ ≃ 75 mm, respectively. From the local time series of *V*_*m*_ mentioned above, we obtain the plots of the IBI shown in Figures 4I–L and the power spectra of Figures 4M–P; discrete peaks in *E*(ω) appear at the fundamental frequency ω_*f*_ ≃ 4.75 Hz and at the frequencies listed in the caption of Figure 5; these peaks indicate that, in Figures 5M,N, we also have some high-order cycles; the broad-band power spectra in Figures 5O,P provide evidence for spiral turbulence with a meandering spiral (SMST). In Figures 5Q–T, we show Poincaré-type return maps which we obtain from the IBI plots in Figures 5I–L; in these maps successive points are connected by lines. These plots give additional evidence for five cycles in Figures 5I,J,M,N and of chaotic behavior in Figures 5K,L,O,P. The lines in Figures 5Q,R move from the bottom-left corner to the top-right corner; this suggests a low-frequency modulation of the spiral-wave dynamics because of the PD; this is associated with the upward trend in the IBI plots of Figures 5I,J.

**Figure 6:**
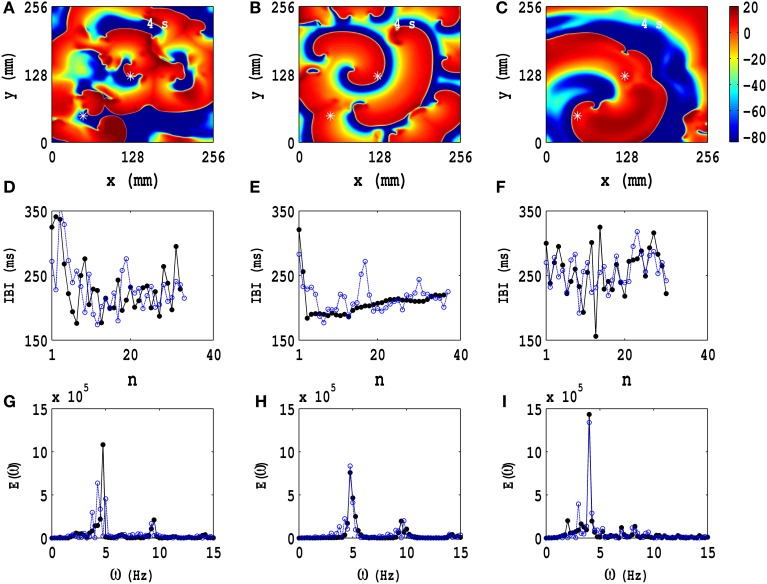
**Temporal evolution of representative ST states in the TP06 model with PD along both spatial directions**. Three ST states, which we obtain with the initial condition *IC*1 and PD, are shown via pseudocolor plots of the transmembrane potential *V*_*m*_ with **(A)** *A*_*x*_ = *A*_*y*_ = 0.3, *f*_*x*_ = *f*_*y*_ = 3.0 Hz, **(B)** *A*_*x*_ = *A*_*y*_ = 0.3, *f*_*x*_ = *f*_*y*_ = 5.0 Hz, and **(C)** *A*_*x*_ = *A*_*y*_ = 0.4, *f*_*x*_ = *f*_*y*_ = 7.0 Hz; the animations (b3), (c3), and (d4) in Video S3 show, respectively, the spatiotemporal evolution of *V*_*m*_ for these cases in the time interval 0 s ≤ *t* ≤ 4 s. We obtain the local time series of *V*_*m*_(*x, y, t*), from the representative points (*x* = 125 mm, *y* = 125 mm) and (*x* = 50 mm, *y* = 50 mm), shown by asterisks in **(A–C)**; from these local time series, we obtain the plots of the IBI **(D–F)** and the power spectra **(G–I)**, with open-blue and black-filled circles for the time series from (*x* = 125 mm, *y* = 125 mm) and (*x* = 50 mm, *y* = 50 mm), respectively. These pseudocolor plots and animations of *V*_*m*_ and the plots of the IBI and power spectra show that we have, roughly speaking, three types of ST states with **(A)** multiple spirals, **(B)** a stable spiral core with broken spiral arms, and **(C)** a single dominant meandering spiral; the second case **(B)** displays a coexistence of a quasiperiodic and an ST state because of the dominant spiral at the center and the broken spirals generated from its arm.

We focus next on the types of ST states that we obtain, with PD applied along both *x* and *y* axes, when we start with the *IC*1 initial condition. In Figure 6 we show three representative ST states; Figures 6A–C show, respectively, pseudocolor plots of the transmembrane potential *V*_*m*_ for PD with (a) *A*_*x*_ = *A*_*y*_ = 0.3, *f*_*x*_ = *f*_*y*_ = 3.0 Hz, (b) *A*_*x*_ = *A*_*y*_ = 0.3, *f*_*x*_ = *f*_*y*_ = 5.0 Hz, and (c) *A*_*x*_ = *A*_*y*_ = 0.4, *f*_*x*_ = *f*_*y*_ = 7.0 Hz; and the animations (b3), (c3), and (d4) in Video S3 show, respectively, the spatiotemporal evolution of *V*_*m*_ for these cases in the time interval 0 s ≤ *t* ≤ 4 s. To examine this evolution, we obtain the local time series of *V*_*m*_(*x, y, t*), from the representative points (*x* = 125 mm, *y* = 125 mm) and (*x* = 50 mm, *y* = 50 mm), both of which are indicated by asterisks in Figures 6A–C; from these local time series, we obtain the plots of the IBI shown in Figures 6D–F and the power spectra of Figures 6G–I, with open-blue and black-filled circles for the time series from (*x* = 125 mm, *y* = 125 mm) and (*x* = 50 mm, *y* = 50 mm), respectively. These pseudocolor plots and animations of *V*_*m*_ and the plots of the IBI and power spectra show that we have, roughly speaking, three types of ST states with (a) multiple spirals (Figure 6A), (b) a stable spiral core with broken spiral arms (Figure 6B), and (c) a single, dominant, meandering spiral (Figure 6C); the second case (b) displays a coexistence of a quasiperiodic and an ST state because of the dominant spiral at the center and the broken spirals generated from its arm. Such coexistence behaviors have been observed in both computational (Xie et al., 2001; Fenton et al., 2002; Cherry and Fenton, 2008) and experimental studies (Nash et al., 2006; Massé et al., 2007), which include *in vivo* experiments.

We also obtain quiescent (Q) states with no spirals because of the absorption of spiral waves at the boundaries as shown in the pseudocolor plots of *V*_*m*_ in Figure S12 in the Supplementary Material S1 and in Video S3.

To illustrate the rich variety of spatiotemporal patterns, we summarize our results for the TP06 model, with the initial condition *IC*1, by presenting a selection of pseudocolor plots of *V*_*m*_ in Figure S13(a1–d5) in the Supplementary Material S1 (for parameter sets see Table 1). The animations in Video S3 show the spatiotemporal evolution of *V*_*m*_ for these cases in the time interval 0 s ≤ *t* ≤ 4 s. To examine this evolution, we obtain the local time series of *V*_*m*_(*x, y, t*), from the representative points (*x* = 125 mm, *y* = 125 mm); these are shown in Figure S14 (Supplementary Material S1); from these local time series, we obtain the plots of the IBI (Figure S15 in the Supplementary Material S1) and the power spectra (Figure S16 in the Supplementary Material S1).

The counterparts of Figures S13–S16 in the Supplementary Material S1, for initial conditions *IC*2 for *IC*3, are given, respectively, in Figures S17–S24 in the Supplementary Material S1.

For the initial conditions *IC*2 and *IC*3 the analogs of the animations in Video S3 are given, respectively, in Videos S4, S5. For *IC*2, with PD along both axes and different values of the amplitude and the frequency, we examine the time series of *V*_*m*_(*x, y, t*), from a representative point in the simulation domain (Figure S18 in the Supplementary Material S1), the plots of the IBI (Figure S19 in the Supplementary Material S1) and the power spectrum *E*(ω) (Figure S20 in the Supplementary Material S1), and the spatiotemporal evolution of *V*_*m*_ (given by the animations in Video S4) and conclude therefrom that, in this case, we obtain either (a) a Q state with no spirals [see animations (a4), (a5), (b4), (b5), (d4) and (d5) in Video S4] or (b) an MST state with broken spiral waves (see the remaining animations in Video S4). A similar analysis, for *IC*3 and PD along both *x* and *y* axes, based on time series of *V*_*m*_ (Figure S22 in the Supplementary Material S1), plots of the IBI (Figure S23 in the Supplementary Material S1), the power spectrum (Figure S24 in the Supplementary Material S1), and the spatiotemporal evolution of *V*_*m*_ (the animations in Video S5) suggests that here we can have (a) a Q state with no spirals [see animations (b3) (b4), (b5), (c4), (d4), and (d5) in Video S5], (b) an SMST state [see animations (a1) and (a5) in Video S5], or (c) an MST state with broken spiral waves (see the rest of the animations in Video S5).

The TNNP04 model with PD also exhibits a rich variety of spatiotemporal patterns with spiral waves like the TP06 model as we discuss in the Supplementary Material S1 (see Figures S25–S36). These figures and the associated Videos S6–S8 show that spiral-wave dynamics with PD in the TNNP04 model is quantitatively different from, but qualitatively similar to, that in the TP06 model. These differences arise because of the differences in the calcium-ion dynamics in these models; such dynamics can play an important role in the spatiotemporal evolution of spiral waves (Weiss et al., 2005; Ter Keurs and Boyden, 2007).

We have discussed spiral-wave dynamics in TP06 and TNNP04 models in the presence of PD along both *x* and *y* directions, with initial conditions of types *IC*1, *IC*2, and *IC*3. We have also carried out systematic simulations of spiral-wave dynamics in both these models, with PD along only one (say *x*) direction. Here too our results are, in the main, qualitatively similar to those we have presented above. Of course, there is anisotropic diffusion if the PD is only along one direction. However, Q, RS, and ST states appear; an overview of their spatiotemporal evolution is given in Figures S37–S42 in the Supplementary Material S1.

### 3.4. Suppression of spiral waves

One of the principal goals of our extensive numerical studies of spiral-wave dynamics in the TP06 and TNNP04 models with PD is to understand its role in enhancing or suppressing spiral-wave turbulence; this is an important step in developing an effective, low-amplitude suppression technique for the elimination of turbulence with single or multiple spirals. So far, various low-amplitude suppression algorithms have been developed to eliminate spiral waves in monodomain mathematical models of cardiac tissue; in these algorithms the control pulses are applied in several ways. These include the following: (a) periodic stimulation at a point (Zhang et al., 2003; Yuan et al., 2005); (b) a line stimulus that must be applied to one of the boundaries (Tang et al., 2008; Miguel et al., 2009); (c) an array of low-voltage control pulses, which must be swept over the simulation domain (Sinha and Sridhar, 2007; Sridhar and Sinha, 2008); or (d) the mesh-based, low-amplitude suppression scheme we describe (Sinha et al., 2001; Pandit et al., 2002). We have provided an overview of such low-amplitude suppression schemes, in the absence of PD, in earlier studies (Shajahan et al., 2009; Nayak, 2013); the most successful of these is based on a mesh-based suppression algorithm; this suppression scheme (Shajahan et al., 2009; Majumder et al., 2011a; Nayak et al., 2013) can suppress spiral waves of electrical activation even in the presence of conduction, ionic, and fibroblast heterogeneities (Shajahan et al., 2009; Majumder et al., 2011a; Nayak et al., 2013). We now investigate the efficacy of this mesh-based suppression scheme for both TP06 and TNNP04 models in the presence of PD.

In this mesh-based suppression scheme, we apply a current pulse of amplitude 75 pA/pF for 0.2 s over a mesh that divides our square simulation domain with *L* = 256 mm into 64 square cells of side *l* = 32 mm each; this pulse makes the links of the mesh refractory and, thereby, effectively imposes Neumann boundary conditions for any block inside the mesh; therefore, spiral waves inside a block are absorbed on the links of the mesh that bound the block. We have also extended this mesh-based scheme to one that uses control pulses on a set of parallel lines; in this line-based scheme, we apply a current pulse of amplitude 125 pA/pF for 0.6 s over a set of parallel lines separated from each other by *l* = 32 mm. As we show in the Supplementary Material S1 (see Figures S43, S44), both these schemes succeed in suppressing spiral-wave turbulence in the TP06 and TNNP04 models without PD; the line-based scheme uses a higher amplitude for the control pulse and a longer duration of application than the mesh-based one because the former has fewer control-pulse segments than the latter. Furthermore, we show (see Figures S45–S48, in the Supplementary Material S1) that the line-based scheme works with PD only if the PD is applied along one spatial direction.

**Figure 7:**
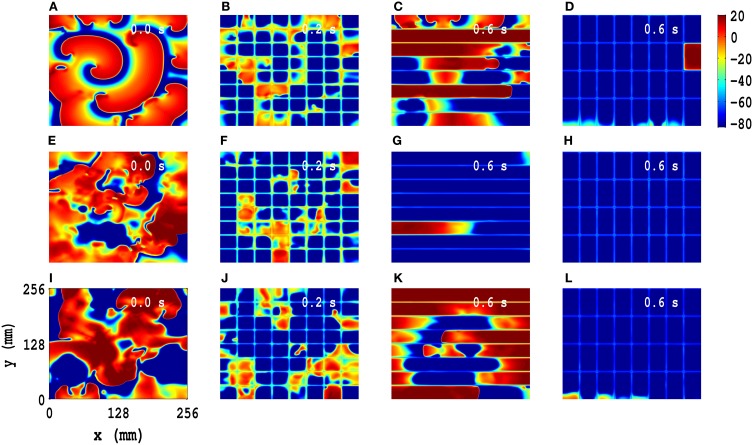
**Comparison of spiral-wave suppression by low-amplitude pulses on square, line, and rectangular control meshes in the TP06 model, with PD along both *x* and *y* directions**. We impose PD along both *x* and *y* directions with the illustrative amplitudes *A*_*x*_ = *A*_*y*_ = 0.3 and frequencies *f*_*x*_ = *f*_*y*_ = 5 Hz for the initial configurations *IC*1, *IC*2, and *IC*3 (pseudocolor plots of *V*_*m*_ in **(A,E,I)**, respectively). We apply the following control pulses: amplitude 75 pA/pF for *t* = 0.2 s over a square mesh **(B,F,J)**, with each square block of side *l* = 32 mm; amplitude 125 pA/pF for *t* = 0.6 s over a line mesh **(C,G,K)**, with inter-line spacing *l* = 32 mm; amplitude 125 pA/pF for *t* = 0.6 s over a rectangular mesh **(D,H,L)**, with block sides *l*_*x*_ = 32 mm and *l*_*y*_ = 64 mm. These pseudocolor plots of *V*_*m*_ and the associated animations in Video S9 show that these spiral states, with *IC*1, *IC*2, and *IC*3 initial conditions, are suppressed by both square- and rectangular-mesh control but not line-mesh control.

A minor modification of our line-based suppression scheme suppresses spiral-wave turbulence: we use a rectangular-mesh-based control scheme, in which we add a few control lines perpendicular to the parallel lines of the line-based suppression scheme. We present a comparison of spiral-wave suppression by low-amplitude pulses on square, line, and rectangular suppression meshes in the TP06 model, with PD along both *x* and *y* directions: We impose PD along both *x* and *y* directions with the illustrative amplitudes *A*_*x*_ = *A*_*y*_ = 0.3 and frequencies *f*_*x*_ = *f*_*y*_ = 5 Hz for the initial configurations *IC*1, *IC*2, and *IC*3 (pseudocolor plots of *V*_*m*_ in Figures 7A,E,I, respectively). We apply the following control pulses: (1) pulses with amplitude 75 pA/pF for *t* = 0.2 s over a square mesh (Figures 7B,F,J), with each square block of side *l* = 32 mm; (2) pulses with amplitude 125 pA/pF for *t* = 0.6 s over a line mesh (Figures 7C,G,K), with inter-line spacing *l* = 32 mm; (3) pulses with amplitude 125 pA/pF for *t* = 0.6 s over a rectangular mesh (Figures 7D,H,L), with block sides *l*_*x*_ = 32 mm and *l*_*y*_ = 64 mm. These pseudocolor plots of *V*_*m*_ and the associated animations in Video S9 show that such spiral-wave states, with *IC*1, *IC*2, and *IC*3 initial conditions, are suppressed by both square- and rectangular-mesh suppression but not by line-mesh suppression. Our rectangular-mesh suppression scheme is a significant improvement over the square-mesh one because it uses fewer control lines than the latter. The results of similar studies for the TNNP04 model are given in Figure S49 (Supplementary Material S1).

## 4. Discussion and conclusion

We have carried out detailed and systematic numerical studies of the effects of periodic deformation (PD) on spiral-wave dynamics in ionically realistic mathematical models for cardiac tissue by introducing PD in the recently developed TP06 and TNNP04 mathematical models for human ventricular tissue (Ten Tusscher et al., 2004; Ten Tusscher and Panfilov, 2006), in which we include deformation as in Zhang et al. (2004, 2006) and Chen et al. (2008). We also investigate, in 2D simulations with PD, the efficacies of square-, rectangular-, and line-mesh-based, low-amplitude suppression techniques to eliminate spiral-wave turbulence in these models for cardiac tissue (Sinha et al., 2001; Pandit et al., 2002; Shajahan et al., 2009; Majumder et al., 2011a).

We have first considered simulations in cable-type domains, which are ideally suited for the calculation of θ and λ. We find that PD leads to a periodic, spatial modulation of θ and a temporally periodic modulation of λ; the degrees of these modulations depend on the amplitude *A*_*x*_ and frequency *f*_*x*_ of the PD. To the best of our knowledge, such modulations have not been quantified in any earlier study, although a few studies (Zhang et al., 2006; Chen et al., 2008) have suggested, in the context of spiral waves, that such modulations can arise because of a Doppler-type effect (Fenton et al., 2002).

We have considered three types of initial spiral-wave configurations, *IC*1, *IC*2, and *IC*3, for both the TP06 and TNNP04 models. In the TP06 model, these configurations evolve, respectively, to (a) an RSC state, (b) an SMST state, and (c) an MST state, in the absence of PD; in the TNNP04 model they evolve, respectively, to (a) an RSC state, (b) an RSZ state, and (c) an MST state, in the absence of PD. We have used such initial conditions because various experimental and computational studies (Damle et al., 1992; Davidenko et al., 1992; Pertsov et al., 1993; Gray et al., 1995; Gray and Jalife, 1996; Beaumont et al., 1998; Gray et al., 1998; Witkowski et al., 1998; Qu et al., 2000a; Fenton et al., 2002; Ten Tusscher and Panfilov, 2006; Shajahan et al., 2009) have suggested that spiral-wave dynamics in cardiac tissue can lead to (a) a stable rotor (Davidenko et al., 1992; Pertsov et al., 1993; Beaumont et al., 1998), as in our RSC state, (b) a single, meandering rotor whose time series is chaotic (Gray et al., 1995; Gray and Jalife, 1996; Qu et al., 2000a), as in our SMST state, and (c) multiple rotors, which yield a state with spatiotemporal chaos (Damle et al., 1992; Gray et al., 1998; Witkowski et al., 1998; Qu et al., 2000a), as in our MST state. Thus, our initial conditions, *IC*1, *IC*2, and *IC*3, lead to the three major types of spiral-wave evolutions, and slight variants thereof (e.g., RSZ), which have been seen in earlier studies and whose evolution we study now with PD. This shows that spiral-wave dynamics in these two models, without PD, depends sensitively on the ionic details of these models and on the initial conditions. A rich variety of spiral-wave behaviors result when we add PD to the TP06 and TNNP04 models, which are quantitatively different from, but qualitatively similar to, that in the TP06 model. These differences arise because of the differences in the calcium-ion dynamics in these models; such dynamics can play an important role in the spatiotemporal evolution of spiral waves (Weiss et al., 2005; Ter Keurs and Boyden, 2007). Our principal findings here can be summarized as follows: In the presence of PD, an RS state may show (a) periodic behavior with high-order cycles in time, (b) temporally quasiperiodic (QP) evolution, (c) a state with spiral-wave turbulence, or (d) a quiescent state Q. For an ST state, which can be of SMST or MST types, PD can either leave the system in an ST state or make it evolve to a Q state, in which all spirals either annihilate each other or are absorbed at the boundaries of the simulation domain. Precisely which one of these states is obtained depends sensitively on our initial conditions and on the PD parameters *A*_*x*_, *A*_*y*_, *f*_*x*_, and *f*_*y*_. This sensitive dependence on parameters is a hallmark of extended dynamical systems that show spatiotemporal chaos (Shajahan et al., 2007, 2009). Thus, our study systematizes the effects of PD on spiral-wave dynamics and turbulence in two, biophysically realistic mathematical models for cardiac tissue; and it complements earlier studies of spiral-wave dynamics, in such models, that have concentrated on the dependence of such dynamics on ion-channel and electrophysiological properties (Karma, 1994; Qu et al., 1999, 2000a,b) and on conduction (Xie et al., 1998; Ten Tusscher and Panfilov, 2003; Shajahan et al., 2007, 2009) and ionic inhomogeneities (Shajahan et al., 2009; Majumder et al., 2011a,b). By using the biophysically realistic TP06 and TNNP04 models for cardiac tissue, our study generalizes the work of Zhang et al. (2004, 2006) and Chen et al. (2008) on spiral-wave instabilities in a simple, two-variable model for cardiac tissue, which is subject to PD.

Moreover, our studies have used three types of spiral-wave initial configurations to examine, via extensive and systematic numerical calculations, the transitions between different states of our system as the amplitude and frequency of the PD are varied. Our work extends significantly earlier studies of PD (Zhang et al., 2004, 2006; Chen et al., 2008) and mechanical deformation (Panfilov et al., 2005, 2007; Weise et al., 2011). Zhang et al. (2004) have studied the instability of a spiral wave of electrical activation by introducing, in a simple, two-variable, FitzHugh-Nagumo-type model (Aliev and Panfilov, 1996) for cardiac tissue, the possibility of periodic, temporal oscillations in the diffusion constant. Their study shows that the resulting deformation can lead to a transition from a stable, RS state to an ST state with multiple spirals. In another study Zhang et al. (2006) have shown that such an ST state can be driven into a quiescent state with no spirals, if the oscillation frequency of the PD is chosen to be close to the characteristic frequency of the spiral wave in the RS state of the system. Chen et al. (2008) have studied the effects of PD in the two-variable, Bär model (Bär and Eiswirth, 1993) on spiral-wave dynamics by varying the parameter ϵ, which sets the time scale of the slow variable in this model, and the amplitude *A* and frequency *f* of the PD; their study shows that the RS-ST transition can be effected by changing ϵ, *A*, and *f* suitably. Panfilov et al. (2007) have shown that mechanical deformation can either (a) induce a spiral wave to drift or (b) break up spiral waves and thus lead to complex spatiotemporal patterns in the three-variable Fenton–Karma model (Fenton and Karma, 1998) for cardiac tissue; the model that (Panfilov et al., 2007) use for deformation is different from, and more realistic than, the one used in Zhang et al. (2004, 2006) and Chen et al. (2008) in so far as it includes a stretch-activated current, which accounts for the mechano-electrical feedback in cardiac tissue, whose stress tensor controls the deformation; their study shows that rotating spirals become unstable both because of the stretch-activated current and the deformation of the tissue. In a related study, which also includes stretch-activated currents, Weise et al. (2011) have shown that such deformation can lead to pacemaker activity in a discrete version of the two-variable, Aliev-Panfilov, reaction-diffusion model (Aliev and Panfilov, 1996). Note that the studies in Zhang et al. (2004, 2006), Panfilov et al. (2007), Chen et al. (2008), and Weise et al. (2011) have used only a particular type of spiral-wave configuration in their two-variable models, with either PD or mechanical deformation. Moreover, Zhang et al. (2004, 2006), Panfilov et al. (2005, 2007), Chen et al. (2008), and Weise et al. (2011) have focused on simple, two-variable, mathematical models for cardiac tissue. Thus, these studies cannot address spiral-wave dynamics in such tissue at the detailed ionic level we consider in our work by using state-of-the-art, ionically realistic mathematical models for ventricular tissue. Furthermore, the authors of Zhang et al. (2004, 2006) and Chen et al. (2008) study the effects of PD on spiral-wave dynamics for a limited set of initial conditions. For example, in Zhang et al. (2004), the authors have studied the behavior of a single rotating spiral (RS) in the presence of PD; the authors of Zhang et al. (2006) have used a broken-spiral state as an initial configuration to study the elimination of spirals from the system in the presence of PD; in Chen et al. (2008), the authors have studied an RS initial configuration and its spatiotemporal evolution with PD. The authors of Panfilov et al. (2005, 2007) and Weise et al. (2011) have used an RS initial configuration to examine the effect of mechanical deformation on this RS state; they have not investigated the transitions between different spiral-wave states. None of these studies have carried out the detailed numerical investigations of spiral-wave dynamics that we present in our work, which considers a variety of initial conditions.

Furthermore, we have shown that square- and line-mesh-based, low-amplitude suppression schemes eliminate spiral-wave turbulence in both the TP06 and TNNP04 models in the absence of PD; this line-based suppression scheme is a significant improvement over the square-mesh suppression scheme of Sinha et al. (2001), Pandit et al. (2002), Shajahan et al. (2009), and Majumder et al. (2011a) because it has fewer control lines. However, we have found that the line-based scheme works with PD only if the PD is applied along one spatial direction. We have then shown that a minor modification of our line-based suppression scheme can suppress spiral-wave turbulence: in particular, we introduce a rectangular-mesh-based suppression scheme, in which we add a few control lines perpendicular to the parallel lines of the line-based suppression scheme; this rectangular-mesh scheme is also a significant improvement because it uses fewer control lines than the one based on a square mesh. We would like to emphasize here that no spiral-wave suppression scheme has been tried in the presence of PD hitherto; our study is the first to address this issue.

The formation of patterns in reaction-diffusion type system with various types of flows have been investigated in Biktashev et al. (1998), Leconte et al. (2003), Kuptsov et al. (2005), and Ermakova et al. (2009); in particular, break up of spiral excitation waves has been observed in a moving excitable medium as suggested in Biktashev et al. (1998); these studies have shown that linear shear flow can cause spiral-wave breakup in an excitable medium. Recent studies in Yoshida (2010) and Yashin et al. (2012a,b) have investigated pattern formation in a gel medium that can be oscillated mechanically; the effect of these mechanical oscillations on the underlying spatiotemporal chemical oscillations, because of a Belousov–Zhabotinsky (BZ) reaction, can be studied in such systems. Our work here presents the cardiac-tissue analogs of such chemical-oscillation studies.

Our study has used methods of non-linear-dynamics to evaluate the effects of periodic deformation on waves of excitation, both plane waves and spiral waves, and has obtained therefrom important physiological implications. In particular, we have shown, for the first time, how spiral-wave turbulence can be suppressed even in the presence of such deformation. This has potential applications in defibrillation because spiral-wave turbulence in our mathematical models is the analog of ventricular fibrillation (Davidenko et al., 1992; Pertsov et al., 1993; Gray et al., 1998; Jalife et al., 1998). Furthermore, our study has immediate implications for experiments on cell cultures with cardiac cells.

Various stretching devices have been developed to control the contraction and expansion of a cell (Barbee et al., 1994; Huang et al., 2010; Wang et al., 2010) and a layer of cells in culture (Lee et al., 1996; Sotoudeh et al., 1998; Waters et al., 2001; Winter et al., 2002; Rana et al., 2008). In these devices, both uniaxial and biaxial (Pang, 2009) stretching methods can be used to deform substrates; moreover, in some of these devices, the stretching can be applied in a cyclic manner at different frequencies. This stretching-induced deformation of the substrate leads, in turn, to the deformation of a layer of culture cells that are attached to the substrate. Examples of such studies include the following: The study in Barbee et al. (1994) has measured the strain that develops in cultured vascular smooth muscle cells when they are deformed by the stretching of a substrate to which they adhere. The authors of Lee et al. (1996) have used a device, which applies homogeneous, equibiaxial strains of 0–10% to a cell-culture substrate, to verify quantitatively the transmission of substrate deformation to a 2D sheet of cultured cardiac cells. The studies in Sotoudeh et al. (1998) have used endothelial cells in tissue culture, on a silicon elastic membrane, and have designed an apparatus that allows for the control of the magnitude and frequency of the dynamical stretching that is applied uniformly to these cells to produce equibiaxial dynamical stretches, with area changes ranging from 0% to 55% and frequencies ranging from 0 to 2 Hz. The authors of Waters et al. (2001) have developed a system for the imposition of cyclic biaxial strain to stretch cultured pulmonary epithelial cells; similar techniques have been used in Winter et al. (2002) to study the effects of strain in cell cultures and *in vitro* experiments. The authors of Rana et al. (2008) have studied the response to such stretching in cultured neonatal rat atrial cardiomyocytes by using a device that can impose homogeneous equibiaxial deformation. Other recent studies include those of Huang et al. (2010) and Wang et al. (2010), which have studied the mechanical activities of living cell, fiber, and tissue by applying both equiaxial and uniaxial deformation, and recording the dynamics of the response of these systems by using high-resolution imaging techniques; the former experiment has used fibroblasts and the latter endothelial cells in culture. We suggest that such experimental studies of the responses of cell cultures to an applied stress can be easily generalized to study the types of problems we have concentrated on here. In particular, by imposing a periodic deformation on cardiac tissue or cell cultures, experiments should be able to verify the predictions we have made, on the basis of our *in silico* studies, about the modulations of θ and λ in the presence of PD and the effects of PD on spiral-wave dynamics, which we have discussed in detail in the previous Section.

We end with some limitations of our model. The first limitation is that our model does not include stretch-activated currents (Panfilov et al., 2005, 2007; Weise et al., 2011) explicitly; but *V*_*m*_ depends on PD and all ionic currents depend on *V*_*m*_, so PD affects all such currents implicitly. Next, the PD in our model affects the electrical activation of our medium but it is not, in turn, affected by this activation; by contrast, the model for mechanical deformation used in Panfilov et al. (2005, 2007) and Weise et al. (2011) allows for electrical feedback to affect such deformation; our model does not include soft-tissue mechanics, which can be incorporated in mathematical models for cardiac tissue by including stress and strain tensors, from elasticity theory (Nash and Hunter, 2000; Nash and Panfilov, 2004; Keldermann et al., 2009), as in the studies of Panfilov et al. (2005, 2007) and Weise et al. (2011); however, these studies use only a two-variable model for cardiac tissue and not the ionically realistic TP06 or TNNP04 models that we employ. Moreover, because of the absence of detailed ion-channel dynamics, the simple, two-variable models for cardiac tissue, which have been used in the studies of Panfilov et al. (2005, 2007) and Weise et al. (2011), do not account for the effects of deformation on ion-channel activity and the intracellular calcium concentration as suggested in Cherubini et al. (2008), Pathmanathan and Whiteley (2009), and Ambrosi et al. (2011). In spite of the simplicity of our model for PD, our study captures various features of spiral-wave dynamics that have been observed in models that include stretch-activated currents (Panfilov et al., 2007); in particular, our model displays spiral-wave breakup because of PD. Thus, this result of ours is robust. The only qualitative effect that our study misses is deformation-induced pacemaker activity, for which it has been argued (Kohl et al., 1999; Panfilov et al., 2005; Weise et al., 2011) that stretch-activated currents are essential. To the best of our knowledge, our elucidation of the effects of PD on spiral-wave dynamics in mathematical models for cardiac tissue, although simple in its modeling of PD, is the first study that explores the effects of PD on spiral-wave dynamics in ionically realistic mathematical models for ventricular tissue. A complete study of a realistic model for deformation, with stretch-activated currents, and such ionically realistic mathematical models lies beyond the scope of the present paper. While this paper was being prepared for publication, a new study appeared on a human-ventricular mathematical model with mechanical deformation (Weise and Panfilov, 2013). The authors of this study have used the same type of deformation that they have employed in their previous investigations of mechanical deformation (Weise et al., 2011); they have studied spiral-wave dynamics in the context of the drifting of a spiral wave as a function of *G*_*s*_ (here, *G*_*s*_ is the maximum conductance of the stretch-activated current). In their numerical studies they have found that, in a constantly stretched medium, there is an increase of the core size and period of a spiral wave, but no change in its rotational dynamics; in contrast, in the dynamically stretched medium, they observe spiral drift. We have found such behaviors, to some extent, in our periodically deformed medium; e.g., the initial condition *IC*1, which leads to a stable rotating spiral with a circular tip trajectory, in the absence of PD, can lead to a variety of behaviors, which can include slow spiral drift in the presence of PD. Our model does not include mechano-electrical feedback in a realistic way, as we have described above. Therefore, we have not attempted to study how different mechanical stimuli, other than the PD we consider, initiate or affect spiral-waves in our model; studies of other mechanical stimuli lie beyond the scope of our paper. In Weise et al. (2011) it has been noted that both electrical and mechanical stimuli can cause the formation of a pacemaker in cardiac tissue; and mechanical stimuli can translate the mechanical energy into an electrical stimulus, as argued in Janse et al. (2003) and Cooper et al. (2006). We use a monodomain description for cardiac tissue; and we do not use an anatomically realistic simulation domain (Panfilov and Keener, 1995; Trayanova and Tice, 2009), muscle-fiber orientation, and transmural heterogeneity (Majumder et al., 2011b, 2012); the inclusion of these features lies beyond the scope of this study. We note, however, that recent studies (Potse et al., 2006) have compared potentials resulting from normal depolarization and repolarization in a bidomain model with those of a monodomain model; these studies have shown that the differences between results obtained from a monodomain model and those obtained from a bidomain model are extremely small.

### Conflict of interest statement

The authors declare that the research was conducted in the absence of any commercial or financial relationships that could be construed as a potential conflict of interest.

## Supplementary material

Video S1 **Spatiotemporal evolution of plane waves in cable-type domains, with PD along the axial-direction of the cables, for the TP06 model, shown via pseudocolor plots of the transmembrane potential V_m_; the time evolution is shown for 0 s ≤ t ≤ 2 s; we use 10 frames per second (fps); in real time each frame is separated from the succeeding frame by 8 ms**.

Video S2 **Spiral-wave dynamics in the TP06 and TNNP04 models in the absence of PD; the time evolution of pseudocolor plots of the transmembrane potential V_m_ is shown for 0 s ≤ t ≤ 4 s; we use 10 frames per second (fps); in real time each frame is separated from the succeeding frame by 8 ms**.

Video S3 **Spiral-wave dynamics for the TP06 model in the presence of PD along both x and y directions with an initial condition of type IC1; the time evolution of pseudocolor plots of the transmembrane potential V_m_ is shown for 0 s ≤ t ≤ 4 s; we use 10 frames per second (fps); in real time each frame is separated from the succeeding frame by 8 ms**.

Video S4 **Spiral-wave dynamics for the TP06 model in the presence of PD along both x and y directions with an initial condition of type IC2; the time evolution of pseudocolor plots of the transmembrane potential V_m_ is shown for 0 s ≤ t ≤ 4 s; we use 10 frames per second (fps); in real time each frame is separated from the succeeding frame by 8 ms**.

Video S5 **Spiral-wave dynamics for the TP06 model in the presence of PD along both x and y directions with an initial condition of type IC3; the time evolution of pseudocolor plots of the transmembrane potential V_m_ is shown for 0 s ≤ t ≤ 4 s; we use 10 frames per second (fps); in real time each frame is separated from the succeeding frame by 8 ms**.

Video S6 **Spiral-wave dynamics for the TNNP04 model in the presence of PD along both x and y directions with an initial condition of type IC1; the time evolution of pseudocolor plots of the transmembrane potential V_m_ is shown for 0 s ≤ t ≤ 4,s; we use 10 frames per second (fps); in real time each frame is separated from the succeeding frame by 8 ms**.

Video S7 **Spiral-wave dynamics for the TNNP04 model in the presence of PD along both x and y directions with an initial condition of type IC2; the time evolution of pseudocolor plots of the transmembrane potential V_m_ is shown for 0 s ≤ t ≤ 4 s; we use 10 frames per second (fps); in real time each frame is separated from the succeeding frame by 8 ms**.

Video S8 **Spiral-wave dynamics for the TNNP04 model in the presence of PD along both x and y directions with an initial condition of type IC3; the time evolution of pseudocolor plots of the transmembrane potential V_m_ is shown for 0 s ≤ t ≤ 4 s; we use 10 frames per second (fps); in real time each frame is separated from the succeeding frame by 8 ms**.

Video S9 **Comparison of spiral-wave suppression by low-amplitude pulses on square, line, and rectangular control meshes in the TP06 model, with PD along both x and y directions, for the time interval 0 s ≤ t ≤ 1 s; we depict pseudocolor plots of V_m_ and use 10 frames per second (fps); in real time each frame is separated from the succeeding frame by 8 ms**.

## References

[B1] Aliev R. R. Panfilov A. V. (1996). A simple two-variable model of cardiac excitation. Chaos Solitons Fractals 7, 293–301 10.1016/0960-0779(95)00089-5

[B2] Ambrosi D. Arioli G. Nobile F. Quarteroni A. (2011). Electromechanical coupling in cardiac dynamics: the active strain approach. SIAM J. Appl. Math. 71, 605–621 10.1137/100788379

[B3] Bär M. Eiswirth M. (1993). Turbulence due to spiral breakup in a continuous excitable medium. Phys. Rev. E 48, R1635 10.1103/PhysRevE.48.R1635 9960866

[B4] Barbee K. A. Macarak E. J. Thibault L. E. (1994). Strain measurements in cultured vascular smooth muscle cells subjected to mechanical deformation. Annal. Biomed. Eng. 22, 14–22 10.1007/BF02368218 8060022

[B5] Barkley D. Kness M. Tuckerman L. S. (1990). Spiral-wave dynamics in a simple model of excitable media: the transition from simple to compound rotation. Phys. Rev. A 42, 2489–2492 10.1103/PhysRevA.42.2489 9904313

[B6] Beaumont J. Davidenko N. Davidenko J. M. Jalife J. (1998). Spiral waves in two-dimensional models of ventricular muscle: formation of a stationary core. Biophys. J. 75, 1–14 10.1016/S0006-3495(98)77490-9 9649363 PMC1299675

[B7] Bernus O. Wilders R. Zemlin C. W. Verschelde H. Panfilov A. V. (2002). A computationally efficient electrophysiological model of human ventricular cells. Am. J. Physiol. Heart Circ. Physiol. 51, H2296–H2308 10.1152/ajpheart.00731.2001 12003840

[B8] Biktashev V. Holden A. Tsyganov M. Brindley J. Hill N. (1998). Excitation wave breaking in excitable media with linear shear flow. Phys. Rev. Lett. 81, 2815 10.1103/PhysRevLett.81.281523345691

[B9] Chen J.-X. Xu J.-R. Yuan X.-P. Ying H.-P. (2008). Influences of periodic mechanical deformation on spiral breakup in excitable media. J. Phys. Chem. B 113, 849–853 10.1021/jp806811j 19113887

[B10] Chen P.-S. Wu T.-J. Ting C.-T. Karagueuzian H. S. Garfinkel A. Lin S.-F. (2003). A tale of two fibrillations. Circulation 108, 2298–2303 10.1161/01.CIR.0000094404.26004.07 14609997

[B11] Chen S.-A. Hsieh M.-H. Tai C.-T. Tsai C.-F. Prakash V. Yu W.-C. (1999). Initiation of atrial fibrillation by ectopic beats originating from the pulmonary veins electrophysiological characteristics, pharmacological responses, and effects of radiofrequency ablation. Circulation 100, 1879–1886 10.1161/01.CIR.100.18.1879 10545432

[B12] Cherry E. Fenton F. (2008). Visualization of spiral and scroll waves in simulated and experimental cardiac tissue. New J. Phys. 10, 125016 10.1088/1367-2630/10/12/125016

[B13] Cherubini C. Filippi S. Nardinocchi P. Teresi L. (2008). An electromechanical model of cardiac tissue: constitutive issues and electrophysiological effects. Prog. Biophys. Mol. Biol. 97, 562–573 10.1016/j.pbiomolbio.2008.02.001 18353430

[B14] Clayton R. Panfilov A. (2008). A guide to modelling cardiac electrical activity in anatomically detailed ventricles. Prog. Biophys. Mol. Biol. 96, 19–43 10.1016/j.pbiomolbio.2007.07.004 17825362

[B15] Cooper P. J. Epstein A. MacLeod I. A. Schaaf S. Sheldon J. Boulin C. (2006). Soft tissue impact characterisation kit (stick) for *ex situ* investigation of heart rhythm responses to acute mechanical stimulation. Prog. Biophys. Mol. Biol. 90, 444–468 10.1016/j.pbiomolbio.2005.07.004 16125216

[B16] Damle R. S. Kanaan N. M. Robinson N. S. Ge Y.-Z. Goldberger J. J. Kadish A. H. (1992). Spatial and temporal linking of epicardial activation directions during ventricular fibrillation in dogs. Evidence for underlying organization. Circulation 86, 1547–1558 10.1161/01.CIR.86.5.1547 1423968

[B17] Davidenko J. M. Kent P. F. Chialvo D. R. Michaels D. C. Jalife J. (1990). Sustained vortex-like waves in normal isolated ventricular muscle. Proc. Natl. Acad. Sci. U.S.A. 87, 8785–8789 10.1073/pnas.87.22.8785 2247448 PMC55044

[B18] Davidenko J. M. Pertsov A. V. Salomonsz R. Baxter W. Jalife J. (1992). Stationary and drifting spiral waves of excitation in isolated cardiac muscle. Nature 355, 349–351 10.1038/355349a0 1731248

[B19] De Bakker J. Van Capelle F. Janse M. J. Tasseron S. Vermeulen J. T. De Jonge N. (1993). Slow conduction in the infarcted human heart.‘zigzag’course of activation. Circulation 88, 915–926 10.1161/01.CIR.88.3.915 8353918

[B20] Ermakova E. A. Shnol E. E. Panteleev M. A. Butylin A. A. Volpert V. Ataullakhanov F. I. (2009). On propagation of excitation waves in moving media: the fitzhugh-nagumo model. PLoS ONE 4:e4454 10.1371/journal.pone.0004454 19212435 PMC2636873

[B21] Fenton F. Karma A. (1998). Vortex dynamics in three-dimensional continuous myocardium with fiber rotation: filament instability and fibrillation. Chaos 8, 20–47 10.1063/1.166311 12779708

[B22] Fenton F. H. Cherry E. M. Glass L. (2008). Cardiac arrhythmia. Scholarpedia 3, 1665 10.4249/scholarpedia.1665

[B23] Fenton F. H. Cherry E. M. Hastings H. M. Evans S. J. (2002). Multiple mechanisms of spiral wave breakup in a model of cardiac electrical activity. Chaos 12, 852–892 10.1063/1.1504242 12779613

[B24] Fogoros R. N. (2011). Ventricular fibrillation. Heart Dis. Available online at: http://heartdisease.about.com/od/palpitationsarrhythmias/a/Ventricular-Fibrillation.htm

[B25] Garfinkel A. Kim Y.-H. Voroshilovsky O. Qu Z. Kil J. R. Lee M.-H. (2000). Preventing ventricular fibrillation by flattening cardiac restitution. Proc. Natl. Acad. Sci. U.S.A. 97, 6061–6066 10.1073/pnas.090492697 10811880 PMC18558

[B26] Gray R. Jalife J. (1996). Self-organized drifting spiral waves as a mechanism for ventricular fibrillation. Circulation 94, I–48

[B27] Gray R. A. Jalife J. Panfilov A. V. Baxter W. T. Cabo C. Davidenko J. M. (1995). Mechanisms of cardiac fibrillation. Science 270, 1222–1225 10.1126/science.270.5239.1222 7502055

[B28] Gray R. A. Pertsov A. M. Jalife J. (1998). Spatial and temporal organization during cardiac fibrillation. Nature 392, 75–78 10.1038/33537 9510249

[B29] Gray R. A. Wikswo J. P. Otani N. F. (2009). Origin choice and petal loss in the flower garden of spiral wave tip trajectories. Chaos 19, 033118 10.1063/1.3204256 19791998 PMC2748696

[B30] Huang L. Mathieu P. S. Helmke B. P. (2010). A stretching device for high-resolution live-cell imaging. Annal. Biomed. Eng. 38, 1728–1740 10.1007/s10439-010-9968-7 20195762 PMC3468334

[B31] Ideker R. E. Rogers J. M. (2006). Human ventricular fibrillation wandering wavelets, mother rotors, or both? Circulation 114, 530–532 10.1161/CIRCULATIONAHA.106.644765 16894047

[B32] Ikeda T. Uchida T. Hough D. Lee J. J. Fishbein M. C. Mandel W. J. (1996). Mechanism of spontaneous termination of functional reentry in isolated canine right atrium evidence for the presence of an excitable but nonexcited core. Circulation 94, 1962–1973 10.1161/01.CIR.94.8.1962 8873675

[B33] Jalife J. Gray R. A. Morley G. E. Davidenko J. M. (1998). Self-organization and the dynamical nature of ventricular fibrillation. Chaos 8, 79–93 10.1063/1.166289 12779712

[B34] Janse M. J. Coronel R. Wilms-Schopman F. J. de Groot J. R. (2003). Mechanical effects on arrhythmogenesis: from pipette to patient. Prog. Biophys. Mol. Biol. 82, 187–195 10.1016/S0079-6107(03)00015-4 12732278

[B35] Karma A. (1994). Electrical alternans and spiral wave breakup in cardiac tissue. Chaos 4, 461–472 10.1063/1.166024 12780121

[B36] Keldermann R. Nash M. Panfilov A. (2009). Modeling cardiac mechano-electrical feedback using reaction-diffusion-mechanics systems. Physica D 238, 1000–1007 10.1016/j.physd.2008.08.017

[B37] Keldermann R. H. Nash M. P. Gelderblom H. Wang V. Y. Panfilov A. V. (2010). Electromechanical wavebreak in a model of the human left ventricle. Am. J. Physiol. Cell Physiol. 299, H134 10.1152/ajpheart.00862.2009 20400690

[B38] Kohl P. Hunter P. Noble D. (1999). Stretch-induced changes in heart rate and rhythm: clinical observations, experiments and mathematical models. Prog. Biophys. Mol. Biol. 71, 91–138 10.1016/S0079-6107(98)00038-8 10070213

[B39] Koller M. L. Riccio M. L. Gilmour R. F Jr. (1998). Dynamic restitution of action potential duration during electrical alternans and ventricular fibrillation. Am. J. Physiol. Heart Circ. Physiol. 275, H1635–H1642 9815071 10.1152/ajpheart.1998.275.5.H1635

[B40] Kuijpers N. H. Potse M. van Dam P. M. ten Eikelder H. M. Verheule S. Prinzen F. W. (2011). Mechanoelectrical coupling enhances initiation and affects perpetuation of atrial fibrillation during acute atrial dilation. Heart Rhythm 8, 429–436 10.1016/j.hrthm.2010.11.020 21075218

[B41] Kuptsov P. V. Satnoianu R. A. Daniels P. G. (2005). Pattern formation in a two-dimensional reaction-diffusion channel with poiseuille flow. Phys. Rev. Ser. E 72, 036216 10.1103/PhysRevE.72.036216 16241557

[B42] Leconte M. Martin J. Rakotomalala N. Salin D. (2003). Pattern of reaction diffusion fronts in laminar flows. Phys. Rev. Lett. 90, 128302 10.1103/PhysRevLett.90.128302 12688909

[B43] Lee A. Delhaas T. Waldman L. K. Mackenna D. A. Villarreal F. J. McCulloch A. D. (1996). An equibiaxial strain system for cultured cells. Am. J. Physiol. Cell Physiol. 271, C1400–C1408 8897847 10.1152/ajpcell.1996.271.4.C1400

[B44] Lim Z. Y. Maskara B. Aguel F. Emokpae R. Tung L. (2006). Spiral wave attachment to millimeter-sized obstacles. Circulation 114, 2113–2121 10.1161/CIRCULATIONAHA.105.598631 17088465

[B45] Majumder R. Nayak A. R. Pandit R. (2012). Nonequilibrium arrhythmic states and transitions in a mathematical model for diffuse fibrosis in human cardiac tissue. PLoS ONE 7:e45040 10.1371/journal.pone.0045040 23071505 PMC3466321

[B46] Majumder R. Nayak A. R. Pandit R. (2011a). An overview of spiral-and scroll-wave dynamics in mathematical models for cardiac tissue, in Heart Rate and Rhythm, eds Tripathi O. N. Ravens U. Sanguinetti M. C. (Berlin; Heidelberg: Springer), 269–282

[B47] Majumder R. Nayak A. R. Pandit R. Majumder R. Nayak A. Pandit R. (2011b). Scroll-wave dynamics in human cardiac tissue: lessons from a mathematical model with inhomogeneities and fiber architecture. PLoS ONE 6:e18052 10.1371/journal.pone.0018052 21483682 PMC3071724

[B48] Massé S. Downar E. Chauhan V. Sevaptsidis E. Nanthakumar K. (2007). Ventricular fibrillation in myopathic human hearts: mechanistic insights from *in vivo* global endocardial and epicardial mapping. Am. J. Physiol. Heart Circ. Physiol. 292, H2589–H2597 10.1152/ajpheart.01336.2006 17259437

[B49] McCulloch A. D. Smaill B. H. Hunter P. J. (1987). Left ventricular epicardial deformation in isolated arrested dog heart. Am. J. Physiol. Heart Circ. Physiol. 252, H233–H241 3812713 10.1152/ajpheart.1987.252.1.H233

[B50] Miguel A. de la Rubia F. J. Ivanov P. C. (2009). Spiral wave annihilation by low-frequency planar fronts in a model of excitable media. EPL (Europhys. Lett.) 86, 18005 10.1209/0295-5075/86/18005

[B51] Nash M. P. Hunter P. J. (2000). Computational mechanics of the heart. J. Elast. Phys. Sci. Solids 61, 113–141 10.1023/A:1011084330767

[B52] Nash M. P. Mourad A. Clayton R. H. Sutton P. M. Bradley C. P. Hayward M. (2006). Evidence for multiple mechanisms in human ventricular fibrillation. Circulation 114, 536–542 10.1161/CIRCULATIONAHA.105.602870 16880326

[B53] Nash M. P. Panfilov A. V. (2004). Electromechanical model of excitable tissue to study reentrant cardiac arrhythmias. Prog. Biophys. Mol. Biol. 85, 501–522 10.1016/j.pbiomolbio.2004.01.016 15142759

[B54] Nayak A. R. (2013). Spiral-Wave Dynamics in Ionically Realistic Mathematical Models for Human Ventricular Tissue. Ph.D. thesis, Indian Institute of Science

[B55] Nayak A. R. Shajahan T. Panfilov A. Pandit R. (2013). Spiral-wave dynamics in a mathematical model of human ventricular tissue with myocytes and fibroblasts. PLoS ONE 8:9 10.1371/journal.pone.0072950 24023798 PMC3762734

[B56] Noble D. (2002). Modelling the heart: insights, failures and progress. Bioessays 24, 1155–1163 10.1002/bies.10186 12447980

[B57] Otani N. F. (2002). A primary mechanism for spiral wave meandering. Chaos 12, 829–842 10.1063/1.1503921 12779611

[B58] Pandit R. Pande A. Sinha S. Sen A. (2002). Spiral turbulence and spatiotemporal chaos: characterization and control in two excitable media. Physica A 306, 211–219 10.1016/S0378-4371(02)00499-5

[B59] Panfilov A. Keldermann R. Nash M. (2007). Drift and breakup of spiral waves in reaction–diffusion–mechanics systems. Proc. Natl. Acad. Sci. U.S.A. 104, 7922–7926 10.1073/pnas.0701895104 17468396 PMC1876548

[B60] Panfilov A. v. Keener J. (1995). Re-entry in an anatomical model of the heart. Chaos Solitons Fractals 5, 681–689 10.1016/0960-0779(93)E0050-L

[B61] Panfilov A. V. Keldermann R. Nash M. (2005). Self-organized pacemakers in a coupled reaction-diffusion-mechanics system. Phys. Rev. Lett. 95, 258104 10.1103/PhysRevLett.95.258104 16384515

[B62] Pang Q. (2009). Design and Development of a Biostretch Apparatus for Tissue Engineering. Ph.D. thesis, University of Toronto 10.1115/1.3005154 20524751

[B63] Pathmanathan P. Whiteley J. P. (2009). A numerical method for cardiac mechanoelectric simulations. Annal. Biomed. Eng. 37, 860–873 10.1007/s10439-009-9663-8 19263223

[B64] Pertsov A. M. Davidenko J. M. Salomonsz R. Baxter W. T. Jalife J. (1993). Spiral waves of excitation underlie reentrant activity in isolated cardiac muscle. Circ. Res. 72, 631–650 10.1161/01.RES.72.3.631 8431989

[B65] Potse M. Dubé B. Richer J. Vinet A. Gulrajani R. M. (2006). A comparison of monodomain and bidomain reaction-diffusion models for action potential propagation in the human heart. IEEE Trans. Biomed. Eng. 53, 2425–2435 10.1109/TBME.2006.880875 17153199

[B66] Qu Z. Weiss J. N. Garfinkel A. (1999). Cardiac electrical restitution properties and stability of reentrant spiral waves: a simulation study. Am. J. Physi. Heart Circ. Physiol. 276, H269–H283 9887041 10.1152/ajpheart.1999.276.1.H269

[B67] Qu Z. Weiss J. N. Garfinkel A. (2000a). From local to global spatiotemporal chaos in a cardiac tissue model. Phys. Rev. E 61, 727 10.1103/PhysRevE.61.727 11046316

[B68] Qu Z. Xie F. Garfinkel A. Weiss J. N. (2000b). Origins of spiral wave meander and breakup in a two-dimensional cardiac tissue model. Annal. Biomed. Eng. 28, 755–771 10.1114/1.1289474 11016413

[B69] Rana O. R. Zobel C. Saygili E. Brixius K. Gramley F. Schimpf T. (2008). A simple device to apply equibiaxial strain to cells cultured on flexible membranes. Am. J. Physiol. Heart Circ. Physiol. 294, H532–H540 10.1152/ajpheart.00649.2007 17965285

[B70] Roger V. L. Go A. S. Lloyd-Jones D. M. Adams R. J. Berry J. D. Brown T. M. (2011). Heart disease and stroke statistics–2011 update a report from the american heart association. Circulation 123, e18–e209 10.1161/CIR.0b013e3182009701 21160056 PMC4418670

[B71] Roger V. L. Go A. S. Lloyd-Jones D. M. Benjamin E. J. Berry J. D. Borden W. B. (2012). Heart disease and stroke statistics–2012 update a report from the american heart association. Circulation 125, e2–e220 10.1161/CIR.0b013e31823ac046 22179539 PMC4440543

[B72] Samie F. H. Jalife J. (2001). Mechanisms underlying ventricular tachycardia and its transition to ventricular fibrillation in the structurally normal heart. Cardiovasc. Res. 50, 242–250 10.1016/S0008-6363(00)00289-3 11334828

[B73] Shajahan T. Nayak A. R. Pandit R. (2009). Spiral-wave turbulence and its control in the presence of inhomogeneities in four mathematical models of cardiac tissue. PLoS ONE 4:e4738 10.1371/journal.pone.0004738 19270753 PMC2650787

[B74] Shajahan T. Sinha S. Pandit R. (2007). Spiral-wave dynamics depend sensitively on inhomogeneities in mathematical models of ventricular tissue. Phys. Rev. E 75, 011929 10.1103/PhysRevE.75.011929 17358206

[B75] Sinha S. Pande A. Pandit R. (2001). Defibrillation via the elimination of spiral turbulence in a model for ventricular fibrillation. Phys. Rev. Lett. 86, 3678–3681 10.1103/PhysRevLett.86.3678 11328052

[B76] Sinha S. Sridhar S. (2007). Controlling spatiotemporal chaos and spiral turbulence in excitable media, in Handbook of Chaos Control, 2nd Edn., eds Schöll E. Schuster H. G. (Weinheim: Wiley Online Library), 703–718 10.1002/9783527622313.ch32

[B77] Sotoudeh M. Jalali S. Usami S. Shyy J. Y. Chien S. (1998). A strain device imposing dynamic and uniform equi-biaxial strain to cultured cells. Annal. Biomed. Eng. 26, 181–189 10.1114/1.48 9525759

[B78] Sridhar S. Sinha S. (2008). Controlling spatiotemporal chaos in excitable media using an array of control points. EPL (Europhys. Lett.) 81, 50002 10.1209/0295-5075/81/50002

[B79] Tang G. Deng M. Hu B. Hu G. (2008). Active and passive control of spiral turbulence in excitable media. Phys. Rev. E 77, 046217 10.1103/PhysRevE.77.046217 18517720

[B80] Ten Tusscher K. Noble D. Noble P. Panfilov A. (2004). A model for human ventricular tissue. Am. J. Physiol. Heart Circ. Physiol. 286, H1573–H1589 10.1152/ajpheart.00794.2003 14656705

[B81] Ten Tusscher K. Panfilov A. (2003). Influence of nonexcitable cells on spiral breakup in two-dimensional and three-dimensional excitable media. Phys. Rev. E 68, 062902 10.1103/PhysRevE.68.062902 14754247

[B82] Ten Tusscher K. Panfilov A. (2006). Alternans and spiral breakup in a human ventricular. Am. J. Physiol. Heart Circ. Physiol. 291, H1088–H1100 10.1152/ajpheart.00109.2006 16565318

[B83] Ter Keurs H. E. Boyden P. A. (2007). Calcium and arrhythmogenesis. Physiol. Rev. 87, 457–506 10.1152/physrev.00011.2006 17429038 PMC4332537

[B84] Thompson S. A. Copeland C. R. Reich D. H. Tung L. (2011). Mechanical coupling between myofibroblasts and cardiomyocytes slows electric conduction in fibrotic cell monolayers. Circulation 123, 2083–2093 10.1161/CIRCULATIONAHA.110.015057 21537003 PMC3176459

[B85] Trayanova N. A. Tice B. M. (2009). Integrative computational models of cardiac arrhythmias–simulating the structurally realistic heart. Drug Discov. Today Dis. Models 6, 85–91 10.1016/j.ddmod.2009.08.001 20628585 PMC2901563

[B86] Valderrábano M. Kim Y.-H. Yashima M. Wu T.-J. Karagueuzian H. S. Chen P.-S. (2000). Obstacle-induced transition from ventricular fibrillation to tachycardia in isolated swine right ventriclesinsights into the transition dynamics and implications for the critical mass. J. Am. Coll. Cardiol. 36, 2000–2008 10.1016/S0735-1097(00)00941-4 11092677

[B87] Wang D. Xie Y. Yuan B. Xu J. Gong P. Jiang X. (2010). A stretching device for imaging real-time molecular dynamics of live cells adhering to elastic membranes on inverted microscopes during the entire process of the stretch. Integr. Biol. 2, 288–293 10.1039/b920644b 20532321

[B88] Waters C. M. Glucksberg M. R. Lautenschlager E. P. Lee C.-W. Van Matre R. M. Warp R. J. (2001). A system to impose prescribed homogenous strains on cultured cells. J. Appl. Physiol. 91, 1600–1610 11568141 10.1152/jappl.2001.91.4.1600

[B89] Weise L. D. Nash M. P. Panfilov A. V. (2011). A discrete model to study reaction-diffusion-mechanics systems. PLoS ONE 6:e21934 10.1371/journal.pone.0021934 21804911 PMC3133613

[B90] Weise L. D. Panfilov A. V. (2013). A discrete electromechanical model for human cardiac tissue: effects of stretch-activated currents and stretch conditions on restitution properties and spiral wave dynamics. PLoS ONE 8:e59317 10.1371/annotation/9ceadf50-eb8f-4051-9e41-772884d47385 23527160 PMC3602082

[B91] Weiss J. N. Qu Z. Chen P.-S. Lin S.-F. Karagueuzian H. S. Hayashi H. (2005). The dynamics of cardiac fibrillation. Circulation 112, 1232–1240 10.1161/CIRCULATIONAHA.104.529545 16116073

[B92] Winter L. C. Gilbert J. A. Elder S. H. Bumgardner J. D. (2002). A device for imposing cyclic strain to cells growing on implant alloys. Annal. Biomed. Eng. 30, 1242–1250 10.1114/1.1529195 12540200

[B93] Witkowski F. X. Leon L. J. Penkoske P. A. Giles W. R. Spano M. L. Ditto W. L. (1998). Spatiotemporal evolution of ventricular fibrillation. Nature 392, 78–82 10.1038/32170 9510250

[B94] Wu T.-J. Lin S.-F. Baher A. Qu Z. Garfinkel A. Weiss J. N. (2004). Mother rotors and the mechanisms of d600-induced type 2 ventricular fibrillation. Circulation 110, 2110–2118 10.1161/01.CIR.0000143834.51102.91 15466637

[B95] Xie F. Qu Z. Garfinkel A. (1998). Dynamics of reentry around a circular obstacle in cardiac tissue. Phys. Rev. E 58, 6355 10.1103/PhysRevE.58.6355

[B96] Xie F. Qu Z. Weiss J. N. Garfinkel A. (2001). Coexistence of multiple spiral waves with independent frequencies in a heterogeneous excitable medium. Phys. Rev. E 63, 031905 10.1103/PhysRevE.63.031905 11308676

[B97] Yashin V. V. Kuksenok O. Dayal P. Balazs A. C. (2012a). Mechano-chemical oscillations and waves in reactive gels. Rep. Prog. Phys. 75, 066601 10.1088/0034-4885/75/6/066601 22790650

[B98] Yashin V. V. Suzuki S. Yoshida R. Balazs A. C. (2012b). Controlling the dynamic behavior of heterogeneous self-oscillating gels. J. Mater. Chem. 22, 13625–13636 10.1039/c2jm32065g

[B99] Yoshida R. (2010). Self-oscillating gels driven by the belousov–zhabotinsky reaction as novel smart materials. Adv. Mater. 22, 3463–3483 10.1002/adma.200904075 20503208

[B100] Yuan G. Wang G. Chen S. (2005). Control of spiral waves and spatiotemporal chaos by periodic perturbation near the boundary. EPL (Europhys. Lett.) 72, 908 10.1209/epl/i2004-10553-2

[B101] Zhang H. Hu B. Hu G. (2003). Suppression of spiral waves and spatiotemporal chaos by generating target waves in excitable media. Phys. Rev. E 68, 026134 10.1103/PhysRevE.68.026134 14525076

[B102] Zhang H. Li B.-W. Sheng Z.-M. Cao Z. Hu G. (2006). The effect of mechanical deformation on spiral turbulence. EPL (Europhys. Lett.) 76, 1109 10.1209/epl/i2006-10391-2

[B103] Zhang H. Ruan X.-S. Hu B. Ouyang Q. (2004). Spiral breakup due to mechanical deformation in excitable media. Phys. Rev. E 70, 016212 10.1103/PhysRevE.70.016212 15324157

[B104] Zimmermann M. Kalusche D. (2001). Fluctuation in autonomic tone is a major determinant of sustained atrial arrhythmias in patients with focal ectopy originating from the pulmonary veins. J. Cardiovasc. Electrophysiol. 12, 285–291 10.1046/j.1540-8167.2001.00285.x 11294170

[B105] Zipes D. P. Wellens H. J. (1998). Sudden cardiac death. Circulation 98, 2334–2351 10.1161/01.CIR.98.21.2334 9826323

