# Supplementary material for: Spiral-wave dynamics in ionically realistic mathematical models for human ventricular tissue: the effects of periodic deformation
Source: Front Physiol. 2014 Jun 10;5:207. doi: 10.3389/fphys.2014.00207 (PMC4050366; doi:10.3389/fphys.2014.00207)
Supplement: Supplementary file 2 [file DataSheet1.PDF]

Supplementary Material S1

Spiral-wave Dynamics in Ionically Realistic Mathematical Models for Human Ventricular Tissue: The Effects of Periodic Deformation

Alok Ranjan Nayak[1,2] **and Rahul Pandit**[1,3,*]
1 Centre for Condensed Matter Theory,
Department of Physics, Indian Institute of Science,
Bangalore 560012, India.
2 Robert Bosch Centre for Cyber Physical Systems,
Indian Institute of Science,
Bangalore 560012, India.
3 Jawaharlal Nehru Centre for Advanced Scientific Research,
Bangalore 560064, India.
∗ *E-mail: rahul@physics.iisc.ernet.in*

In this Supplementary Material S1, We list those parameters that differ from their counterparts in the original TNNP04 and TP06 models presented in Refs. [1, 2]; we also give the values of initialized gating variables and ion concentrations, which we use. Furthermore, we present additional material and figures from our simulations in this Supplementary Material S1.

I. The TNNP04 Model

Parameters

G_{pCa}: Maximal plateau Ca^{2+} current (I_{pCa}) conductance =0.825 nS/pF;

Initialized gating variables and ion concentrations

m: Activation gate for I_{Na} = 0;
h: Fast inactivation gate for I_{Na} = 0.75;
j: Slow inactivation gate for I_{Na} = 0.75;
d: Activation gate for I_{CaL} = 0;
f: Inactivation gate for I_{CaL} = 1;
f_{Ca}: Intracellular calcium-dependent inactivation gate for I_{CaL} = 1;
r: Activation gate for I_{to} = 0;
s: Inactivation gate for I_{to} = 1;
x_s: Activation gate for I_{Ks} = 0;
x_{r1}: Activation gate for I_{Kr} = 0;
x_{r2}: Inactivation gate for I_{Kr} = 1;
g: Calcium-dependent inactivation gate for I_{rel} = 1;
Na_i: Intracellular Na^+ concentration =11.6 mM;
Ca_i: Intracellular Ca^+ concentration =0.0002 mM;
K_i: Intracellular K^+ concentration =138.3 mM;
Ca_{sr}: Free Ca^{2+} concentration in the sarcoplasmic reticulum (SR) =0.2 mM.

II. The TP06 Model

Parameters

V_C: Cytoplasmic volume =16404 μm^3;
V_{SR}: Sarcoplasmic reticulum volume =1094 μm^3;
V_{SS}: Diadic subspace volume =54.68 μm^3. V_{rel}: Maximal I_{rel} conductance =0.102 mM/ms;

k_4: I to O and RI to I I_{rel} transition rate = 0.005 ms^{-1};

Initialized gating variables and ion concentrations

m: Activation gate for I_{Na} = 0;
h: Fast inactivation gate for I_{Na} = 0.75;
j: Slow inactivation gate for I_{Na} = 0.75;
d: Activation gate for I_{CaL} = 0;
f: Slow inactivation gate for I_{CaL} = 1;
f_2: Fast inactivation gate for I_{CaL} = 1;
f_{Cass}: Diadic subspace free calcium-dependent inactivation gate for I_{CaL} = 1;
r: Activation gate for I_{to} = 0;
s: Inactivation gate for I_{to} = 1;
x_s: Activation gate for I_{Ks} = 0;
x_{r1}: Activation gate for I_{Kr} = 0;
x_{r2}: Inactivation gate for I_{Kr} = 1;
$\bar{R}$: Proportion of close I_{rel} channel = 1;
O: Proportion of open I_{rel} channel = 0;
Na_i: Intracellular Na^+ concentration =7.67 mM;
Ca_i: Intracellular Ca^+ concentration =0.00007 mM;
K_i: Intracellular K^+ concentration =138.3 mM;
Ca_{sr}: Free Ca^{2+} concentration in the sarcoplasmic reticulum (SR) =1.3 mM;
Ca_{ss}: Free Ca^{2+} concentration in the diadic subspace (SS) =0.00007 mM.

III. Results

a. A Single Myocyte Cell

It is important to check the compatibility of the models that we consider in our study with the standard version of the original models [1,2]. One way to check for such compatibility is by comparing the AP and its morphological properties with those in our studies. We find that our studies, at single-cell level, are consistent with those in the original models as we discuss below.

We calculate morphological properties of the AP, such as, the action-potential duration APD, the resting-membrane potential V_{rest}, the maximum upstroke velocity dV/dt_{max}, the maximum value of V_m, during the action potential, namely, V_{max}, the value of V_m at the position of the notch, i.e., V_{notch}, and the maximum value of V_m, in the plateau region of the action potential, i.e., $V_{plateau}$. We apply a series of 50 current pulses of amplitude 52 pA/pF for 1 ms before an application of the final stimulus to measure properties that characterize the AP morphology; these initial 50 stimuli bring the system into the statistically steady state (so that we can avoid the effects of transients). We list the values of the AP-morphological properties in Table S1, for both the TP06 and TNNP model. These values, which we have obtained, are consistent with the results for the original TP06 and TNNP04 models [1,2]. Furthermore, to check the consistency of the models that we have used, we give plots of AP that we obtain from our studies and the original models formulated in Refs. [1,2]. In Fig. S1(a) and (b) we show, respectively, the plots of the APs for the TP06 and TNNP04 model for epicardial cells; the thick, blue lines show the APs that we have obtained from our computer code; and the thin, red lines show the APs obtained from the original TP06 and TNNP04 code (see the webpage of ten Tusscher at http://www-binf.bio.uu.nl/khwjtuss/SourceCodes/).

Model	$APD_{90\%}$ (ms)	V_{max} (mV)	$\dot{V}_{max}$ (mV/ms)	V_{notch} (mV)	$V_{plateau}$ (mV)	V_{rest} (mV)
TP06	≃ 306	≃ 40.4	≃ 304	≃ 22.4	≃ 26.3	≃ -85.9
TNNP04	≃ 278	≃ 35.5	≃ 301	≃ 13.6	≃ 22.1	≃ -86.8

Table S1. Action potential (AP) morphological values for a single, epicardial, ventricular cell in the TP06 and TNNP04 mathematical models (see text).

b. Numerical Stability

We set the diffusion coefficients $D_x = D_y = D = 0.00154$ cm^2/ms [1, 2] for both the TP06 and the TNNP04 models and examine the oscillation-amplitude and the frequency ranges $0 \lesssim A_x = A_y \lesssim 0.5$, and 0 Hz $\lesssim f \lesssim 7.0$ Hz, respectively; the deformation amplitudes we use are comparable to those in other computational studies [3–5]. Reference [6] suggests that we must have $D\delta t/(\delta x^2) < 1/2d$ for numerical stability, where d is the dimension of the simulation domain. For the largest amplitude we use for the PD, the minimum and maximum values of our time-dependent diffusion coefficient are, respectively, $0.00154 \times (1 + 0.5)^{-2} \simeq 0.00068$ cm^2/ms and $0.00154 \times (1 - 0.5)^{-2} \simeq 0.00616$ cm^2/ms between which our time-dependent diffusion coefficient oscillates. Therefore, for the time and space steps we have used in our calculations, the maximum and minimum values of $D\delta t/(\delta x)^2$ are, respectively, 0.197 and 0.022; for our 2D domain, the quantity $1/2d = 0.25$, i.e., we have numerical stability because $D\delta t/(\delta x)^2 < 1/2d$ for all values of our time-dependent diffusion coefficient. We have also checked that the changes of CV, which occur when we change either our time step or space step, are not significant so our simulation results for CV are not numerical artifacts. We can test, in one more way, that our results are free from numerical artifacts by checking the spatiotemporal evolution of an expanding wave front that emerges from a point stimulus [6]; if this wave front deviates substantially from a circular wave, then spiral-wave dynamics in the simulation may be represented inaccurately. Therefore, we have carried out a set of simulations by applying a stimulus at a point at the center of the simulation domain in the presence of PD. We apply a stimulus of current density 450 pA/pF for 9 ms at the center of our square simulation domain of side $L_x = L_y = 256$ mm. Our numerical results show that fronts of the expanding wave do not deviate substantially from circles, even when we include PD along both x and y directions, with amplitudes and frequencies in the ranges $0 \le A_x = A_y \le 0.5$ and 1.0 Hz $\le f_x = f_y \le 7.0$ Hz, respectively. The spatiotemporal evolution of such expanding waves is shown for three representative cases of PD, with amplitude and frequency (a) $A_x = A_y = 0.1$, $f_x = f_y = 5$ Hz, (b) $A_x = A_y = 0.3$, $f_x = f_y = 5$ Hz, and (c) $A_x = A_y = 0.5$, $f_x = f_y = 5$ Hz, respectively, in Fig. S3 by pseudocolor plots of V_m.

c. Wave Dynamics in a 2D Simulation Domain

1. Spiral-wave Initiation

In the absence of periodic deformation (PD), two methods are used to initiate spiral waves in simulations [1, 7–9] and experiments [7, 10], namely, (1) the S1-S2 cross-field protocol and (2) the S1-S2 parallel-field protocol. In the cross-field method, a super-threshold stimulus S2 is applied at the boundary that is perpendicular to the S1 stimulus, whereas, in the parallel-field method, S2 is applied parallel to the refractory tail of the S1 stimulus, but not over the entire length of the domain. Our simulation does not show spiral-wave formation if we use the S1-S2 cross-field protocol; this protocol leads to an initial spiral hook near the edge of the simulation domain; and this hook is absorbed by the boundary before it can develop into a spiral wave. However, the parallel-field protocol does lead to the formation of a spiral wave in the medium; in Refs. [9, 11] we have shown that the location of the center of the spiral depends sensitively on the time of initiation of the S2 stimulus and on its spatial extent. We have found that the following modification of the cross-field protocol yields spiral waves easily. In this modified protocol, the precise position and the application time of the S2 stimulus does not have to be controlled as carefully as in the parallel-field protocol; the principal requirement is that the S2 pulse must be applied before the wave back of the S1 stimulus travels across the full simulation domain. In particular, to initiate a spiral wave in our square simulation domain with sides $L_x = L_y = 256$ mm, we first apply an S1 stimulus, of strength 150 pA/pF, for 3 ms; this injects a plane wave at the left boundary of this domain; we then apply an S2 stimulus, of the same strength as the S1 stimulus and for the same duration, to the bottom half of the domain (i.e., 0 mm $\le y \le$ 125 mm).

2. Spiral-wave Dynamics in a Homogeneous Domain

The procedure that we describe above yields the fully developed spiral waves shown in Fig. S5, for the TP06 model; the analogs of Fig. S5, for the TNNP04 model, is shown in Fig. S6. We use three types of spiral-wave initial configurations for our subsequent studies; we refer to these as $IC1$, $IC2$, and $IC3$ initial conditions (see Table 1, in the main paper, for parameter values).

In Figs. 3(a)-(c) in the main paper, we show pseudocolor plots of V_m at times $t = 0$ s, $t = 2$ s, and $t = 4$ s, respectively, for the initial condition *IC1* in the TP06 model, in the absence of PD; this initial

configuration evolves to a state with a rotating spiral (RS) in the medium; the animation (a) in Video S2 shows the spatiotemporal evolution of V_m for this case. The local time series of $V_m(x,y,t)$, from the representative point ($x = 125$ mm, $y = 125$ mm) (the asterisk in Fig. 3(c) in the main paper), is shown in Fig. 3(d) (main paper) for 2 s $\leq t \leq$ 6 s; a plot of the inter beat interval (ibi) is given in Fig. 3(e) (main paper), which shows that, after initial transients (roughly the first 10 beats), the spiral wave rotates periodically with an average rotation period $T \simeq 210$ ms. In Fig. 3(f) (main paper), we plot the power spectrum $E(\omega)$, which we have obtained from the local time series of V_m mentioned above; discrete peaks in $E(\omega)$ appear at the fundamental frequency $\omega_f \simeq 4.75$ Hz and its harmonics. The periodic nature of the local time series of V_m, the flattening of the ibi, and the discrete peaks in $E(\omega)$ show that the temporal evolution of the spiral wave is periodic; therefore, the spiral-tip trajectory traces a roughly circular path with radius $l_c \simeq 20$ mm; this circular path is shown, for 3.6 s $\leq t \leq$ 4 s, by the white line that has been superimposed on the pseudocolor plot of V_m in Fig. 3(c) (main paper); an expanded version of this path is shown in Fig. 3(g) (main paper).

In Figs. S7(a)-(g) we show, for the initial condition *IC2* in the absence of PD, the exact analogs of Figs. 3(a)-(g) (see main paper); and the animation (b) in Video S2 shows the spatiotemporal evolution of V_m for this case. This animation, the pseudocolor plots of V_m (Figs. S7(a)-(c)), the representative local time series of V_m (Fig. S7(d)), the plot of the ibi (Fig. S7(e)), the power spectrum $E(\omega)$ (Fig. S7(f)), and the spiral-tip trajectory (the white curve in Fig. S7(c) and the blue one in Fig. S7(g)) show that the initial condition *IC2* leads to spatiotemporal chaos and spiral turbulence (ST), with a single spiral meandering chaotically in the simulation domain.

In Figs. S8(a)-(f) we show, for the initial condition *IC3* in the absence of PD, the exact analogs of Figs. 3(a)-(f)(see main paper); and the animation (c) in Video S2 shows the spatiotemporal evolution of V_m for this case. This animation, the pseudocolor plots of V_m (Figs. S8(a)-(c)), the representative local time series of V_m (Fig. S8(d)), the plot of the ibi (Fig. S8(e)), and the power spectrum $E(\omega)$ (Fig. S8(f)) show that the initial condition *IC3* leads to spatiotemporal chaos and spiral turbulence (ST) with broken spirals in the simulation domain; the life-span of a given spiral-wave segment is small so we do not track the trajectories of spiral tips in this case.

Figures S9(a)-(g), Figs. S10(a)-(g), and Figs. S11(a)-(f) show, respectively, the TNNP04 analogs of the TP06 Figs. 3(a)-(g) in the main paper (for *IC1*), Figs. S7(a)-(g) (for *IC2*), and Figs. S8(a)-(f) (for *IC3*); the spatiotemporal evolution of V_m for these three initial conditions for the TNNP04 model are given in animations (d), (e), and (f) in Video S2. From these animations and the Figs. S9(a)-(g), S10(a)-(g), and S11(a)-(f) we conclude that the spatiotemporal evolution of V_m in the TNNP04 model, without PD, is similar to, but not identically the same as, that in the TP06 model for the initial conditions *IC1*, *IC2*, and *IC3*. One difference is that, in the TNNP04 model, we have a Z-type, spiral-tip trajectory in Figs. S10(c) and (g), whereas, for the same initial condition, we have an open spiral-tip trajectory (Figs. S7(c) and (g)) in the TP06 model.

3. Spiral-wave Dynamics with PD

We have presented our principal results, with PD and the initial condition $IC1$ for the TP06 model, in the main paper. Here we present the additional results with PD and all the initial conditions we use for both TP06 and TNNP04 models.

For the TP06 model and the initial condition *IC1*, we obtain quiescent (Q) states with no spirals because of the absorption of spiral waves at the boundaries of the simulation domain. Typically, this occurs because a single spiral wave drifts towards the boundaries, as we illustrate by the pseudocolor plots of V_m in Figs. S12(a)-(c), for $t = 0.4$ s, and Figs. S12(d), (e), and (f), for $t = 0.8$ s, $t = 1.2$ s, and $t = 1.6$ s, for three representative cases with the following PDs: (a) $A_x = A_y = 0.4$, $f_x = f_y = 3.0$ Hz (Figs. S12(a) and (d)), (b) $A_x = A_y = 0.5$, $f_x = f_y = 1.0$ Hz (Figs. S12(b) and (e)), and (c) $A_x = A_y = 0.5$, $f_x = f_y = 3.0$ Hz (Figs. S12(c) and (f)); the pseudocolor plots of V_m near the final, spiral-absorption states are shown in Figs. S12(d)-(f). The spatiotemporal evolution of V_m is shown in the animations (b4), (a5), and (b5) in Video S3 for the time interval 0 s $\leq t \leq$ 4 s.

The TNNP04 model with PD also exhibits a rich variety of spatiotemporal patterns with spiral waves like the TP06 model. The initial conditions we use are shown via pseudocolor plots of V_m in Fig. S6. In particular, for initial conditions of types *IC1*, *IC2*, and *IC3* (Fig. S6(c), (f), and (i), respectively), the TNNP04 analogs of the pseudocolor plots of V_m (see, Fig. S13, and Fig. S17 and Fig. S21, here, for the TP06 model) are given in Fig. S25, Fig. S29, and Fig. S33, respectively, and the Videos S6, S7, and S8. The time series of $V_m(x,y,t)$, from a representative point in the simulation domain, and the associated

plots of the ibi and the power spectra are given in Figs. S26, S27, and S28, for *IC1*, Figs. S30, S31, and S32, for *IC2*, and Figs. S34, S35, and S36, for *IC3*. These figures and videos show that spiral-wave dynamics with PD in the TNNP04 model is quantitatively different from, but qualitatively similar to, that in the TP06 model. In particular, the TNNP04 model shows several quiescent states Q, e.g, (i) for *IC1*, in Fig. S25(b5) and the animation (b5) in Video S6, (ii) for *IC2*, in Figs. S29(d4) and (d5) and the animations (d4) and (d5) in Video S7, and (iii) for *IC3*, in Figs. S33(a1), (b2), (b3), (c4) and (c5) and the animations (a1), (b2), (b3), (c4), and (c5) in Video S8; we obtain states with RS, which are associated with cyclic motions of various orders, in Figs. S27(a2), (a3), (c1), (c3), (c4), (d1), (d2), (d3) and (d4), for *IC1*, Figs. S31(a1), (a2), (a3), (b1), (c1), (c2) and (d1), for *IC2*; finally, we observe a variety of states with spiral-wave turbulence in Fig. S27(c5) and the animation (c5) in Video S6, for *IC1*, Figs. S31(b5), (c4) and (c5) and the animations (b5), (c4) and (c5) in Video S7, for *IC2*, and Figs. S35(a2), (a3), (a4), (a5), (b1), (b4), (b5), (c1), (c2), (c3), (d1), (d2), (d3), (d4) and (d5) and the animations (a2), (a3), (a4), (a5), (b1), (b4), (b5), (c1), (c2), (c3), (d1), (d2), (d3), (d4) and (d5) in Video S8, for *IC3*.

4. Suppression of Spiral Waves

We have discussed in detail, in the main paper, our scheme for the suppression of spiral waves in mathematical models for cardiac tissue. In particular, we have compared square- and line-mesh suppression techniques, with and without PD, in the "Results" Section of the main paper. Here we present additional details of our study of spiral-wave suppression with square- and line-mesh suppression techniques.

In Fig. S43 we illustrate spiral-wave suppression, via low-amplitude control pulses, in the TP06 model, in the absence of PD, by presenting pseudocolor plots of V_m. The spiral state, at time t = 0 ms (Fig. S43(a) with the $IC1$ initial condition), evolves, in the absence of the control, to an RS state (Fig. S43(b)) at time t = 0.2 s; this state is suppressed, by both square- and line-mesh suppression methods, as shown in Fig. S43(c) and Fig. S43(d), at t = 0.2 ms and t = 0.6 ms, respectively. Similar plots for the $IC2$ and $IC3$ initial conditions, Figs. S43(e)-(h) and Figs. S43(i)-(l), respectively, illustrate square- and line-mesh suppression of states with spiral turbulence. The results of similar studies for the TNNP04 model are given in Fig. S44.

In Fig. S45 we illustrate spiral-wave suppression via pseudocolor plots of V_m by low-amplitude pulses in the TP06 model with PD along the x direction and the illustrative amplitude A_x = 0.3 and frequency f_x = 5 Hz; for this case Figs. S45 (a)-(l) are the analogs of Figs. S43(a)-(l), respectively. With the initial condition *IC1*, the spiral in Fig. S45(a), at t = 0 ms, evolves, in the absence of control, to an MST state (Fig. S45(b)) at t = 0.2 s; however, this MST can be suppressed by both square- and line-mesh suppression techniques as shown in Fig. S45(c) and Fig. S45(d) at t = 0.2 ms and 0.6 ms, respectively. For the *IC2* and *IC3* initial configurations, the analogs of these states are shown in Figs. S45(e)-(h) and Figs. S45(i)-(l), respectively; clearly, both our suppression schemes are successful in eliminating spiral turbulence with PD along one direction. The results of similar studies for the TNNP04 model are given in Fig. S46.

We turn now to spiral-wave suppression in the TP06 model, with PD along both x and y directions and the amplitudes A_x = A_y = 0.3 and frequencies f_x = f_y = 5 Hz; for this case Figs. S47 (a)-(l) are the analogs of Figs. S45 (a)-(l), respectively. With the initial condition *IC1*, the spiral in Fig. S47(a), at t = 0 ms, evolves, in the absence of the control, to the MST state in Fig. S47(b) at t = 0.2 s; this MST can be suppressed by the square-mesh technique but not by the line-mesh technique as we show in Fig. S47(c) and Fig. S47(d) at t = 0.2 ms and 0.6 ms, respectively. For the initial conditions *IC2* and *IC3* the analogs of these states are shown, respectively, in Figs. S47(e)-(h) and Figs. S47(i)-(l). Thus, with PD along both directions, spiral turbulence can be suppressed by our square-mesh suppression but not the line-mesh method for $IC1$ and $IC3$ initial conditions. The results of similar studies for the TNNP04 model are given in Fig. S48.

We present a comparison of spiral-wave suppression by low-amplitude pulses on square, line, and rectangular suppression meshes in the TP06 model, with PD along both x and y directions in Fig. 7 in the main paper; the analog plot of Fig. 7 for the TNNP04 model is present in Fig. S49.

References

1. ten Tusscher KHWJ, Noble D, Noble PJ, *et al.* (2004) A model for human ventricular tissue. *Am J Physiol Heart Circ Physiol* 286: H1573.

2. ten Tusscher KH, Panfilov AV (2006) Alternans and spiral breakup in a human ventricular tissue model. *Am J Physiol Heart Circ Physiol* 291: H1088.

3. Zhang H, Ruan X, Hu B, Ouyang Q (2004) Spiral breakup due to mechanical deformation in excitable media. *Phys Rev E* 70: 016212.

4. Zhang H, Li BW, Sheng ZM, *et al.* (2006) The effect of mechanical deformation on spiral turbulence. *Europhys Lett* 76: 1109.

5. Weise LD, Nash MP, Panfilov AV (2011) A discrete model to study reaction-diffusion-mechanics systems. *PLoS One* 6(7): e21934.

6. Clayton RH, Panfilov AV (2008) A guide to modelling cardiac electrical activity in anatomically detailed ventricles. *Prog Biophys Mol Biol* 96: 19.

7. Pertsov AM, Davidenko JM, Salomonsz R, *et al.* (1993) Spiral waves of excitation underlie reentrant activity in isolated cardiac muscle. *Circ Res* 72: 631.

8. Bernus O, Wilders R, Zemlin CW, *et al.* (2002) A computationally efficient electrophysiological model of human ventricular cells. *Am J Physiol Heart Circ Physiol* 282: H2296.

9. Shajahan TK, Nayak AR, Pandit R (2009) Spiral-wave turbulence and its control in the presence of inhomogeneities in four mathematical models of cardiac tissue. *PLoS One* 4(3): e4738.

10. Davidenko JM, Perstov AV, Salomonsz R, *et al.* (1992) Stationary and drifting spiral waves of excitation in isolated cardiac muscle. *Nature* 355: 349.

11. Majumder R, Nayak AR, Pandit R (2011) An overview of spiral- and scroll-wave dynamics in mathematical models for cardiac tissue. *Heart Rate and Rhythm: Molecular Basis, Pharmacological Modulation and Clinical Implications*, Chapter 14, pp 269.

IV. Figures

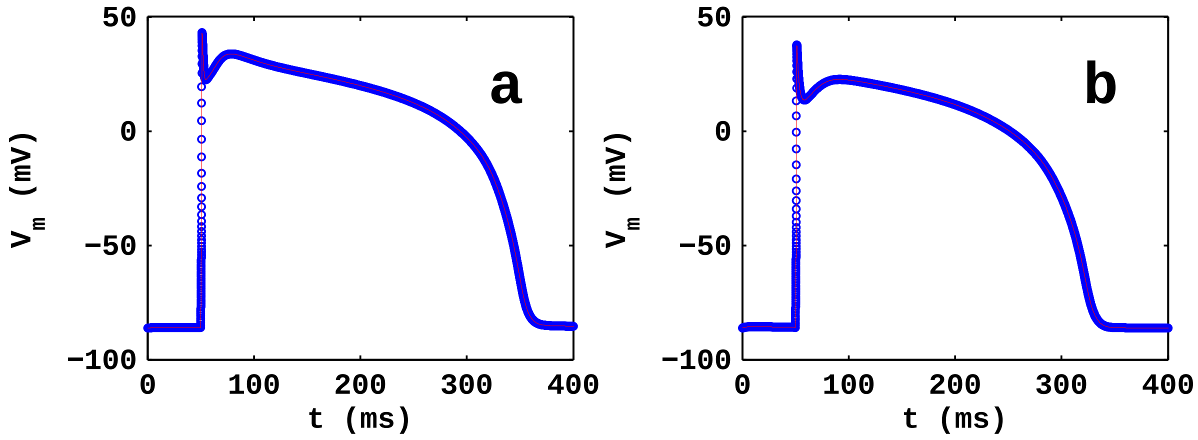

Figure S1. Action potentials (APs) obtained from our source code (written in the *C* language) and original source code (obtained from the web page of ten Tusscher webpage and written in the *C* + + language) for (a) the TP06 model and (b) the TNNP04 model. The blue, open circles and red, thin lines represent, respectively, the plots obtained from the original *C* + + code and our *C* source code (see text).

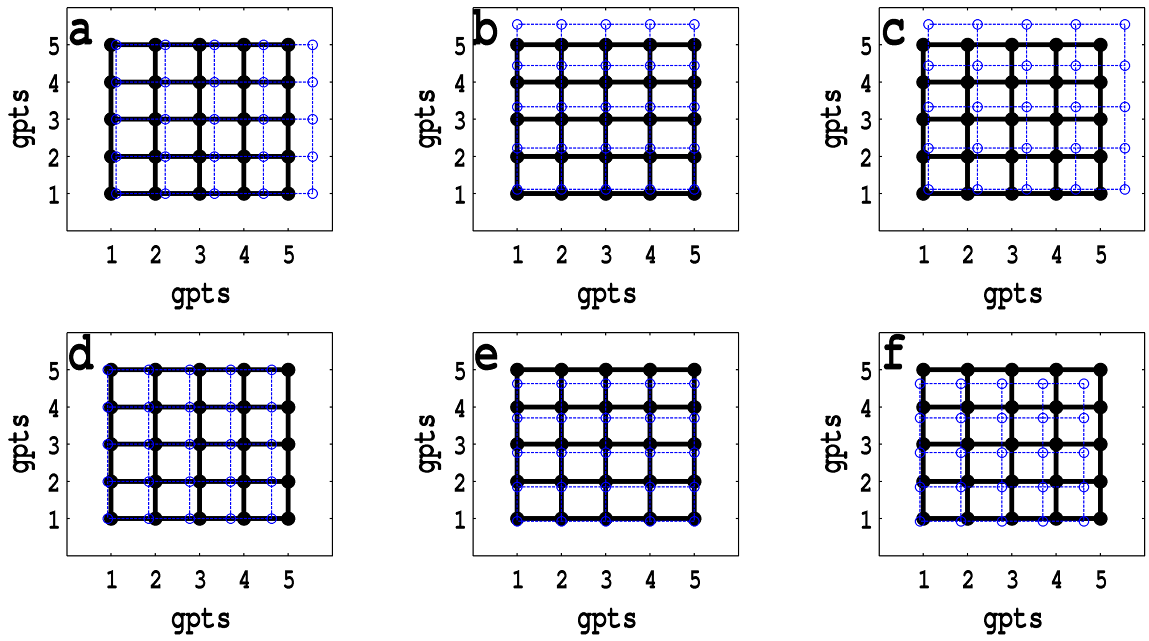

Figure S2. Schematic diagrams for illustrative periodic deformations of a small part of the simulation domain, with 5 × 5 grid points; in these diagrams, blue, open circles and blue, dashed lines show, at a particular instant of time, the deformed simulation domain superimposed on the undeformed one, which is represented by black, solid circles and black, full lines. The case of expansion, with deformations along only x (with amplitude $A_x = 0.3$ and frequency $f_x = 3$ Hz), only y (with amplitude $A_y = 0.3$ and frequency $f_y = 3$ Hz), or both x and y directions (with amplitudes $A_x = A_y = 0.3$ and frequencies $f_x = f_y = 3$ Hz) are shown, respectively, in (a), (b) and (c), at time $t = 20$ ms; the corresponding plots for contraction, at time $t = 180$ ms, are shown in (d), (e), and (f).

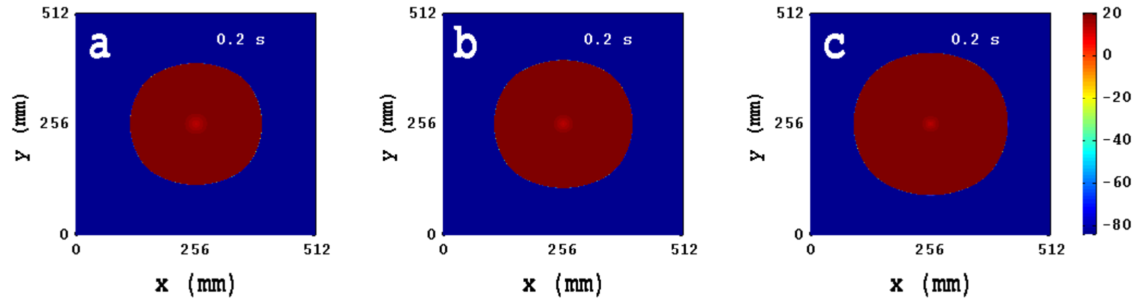

Figure S3. Pseudocolor plots of the transmembrane potential V_m for the TP06 model, in a square domain, with PD along along both x and y directions and an initial condition of type $IC1$; (a) $A_x = A_y = 0.1$, $f_x = f_y = 5$ Hz, (b) $A_x = A_y = 0.3$, $f_x = f_y = 5$ Hz, and (c) $A_x = A_y = 0.5$, $f_x = f_y = 5$ Hz. These plots show that the wave fronts do not deviate substantially from circles when we include PD along both x and y directions.

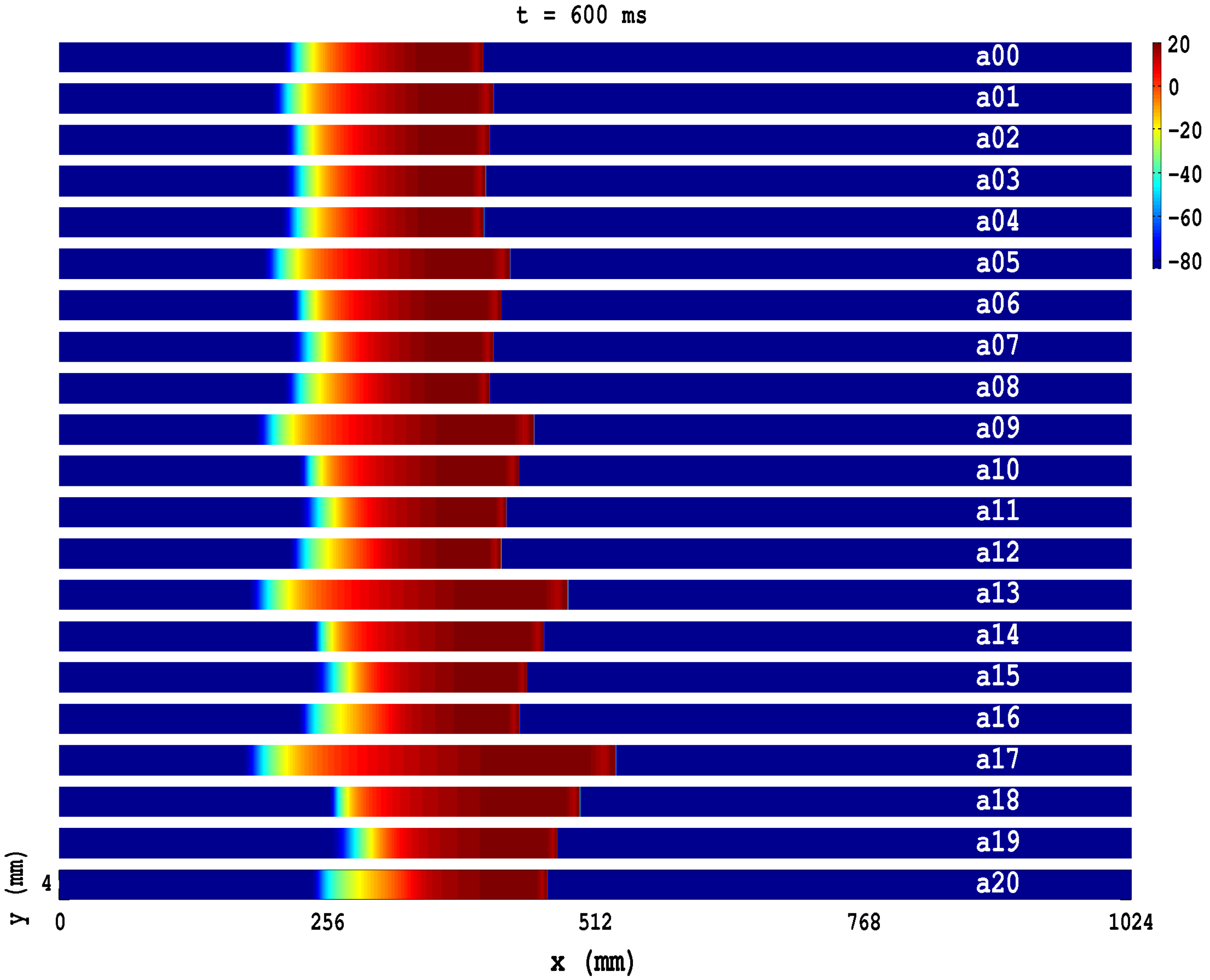

Figure S4. Pseudocolor plots of the transmembrane potential V_m for the TNNP04 model illustrating plane-wave propagation in a cable-type domain, with PD along the axial-direction of the cable, and the following parameter sets: (a00) no PD; (a01) $A_x = 0.1$, $f_x = 1.0$ Hz; (a02) $A_x = 0.2$, $f_x = 1.0$ Hz; (a03) $A_x = 0.3$, $f_x = 1.0$ Hz; (a04) $A_x = 0.4$, $f_x = 1.0$ Hz; (a05) $A_x = 0.5$, $f_x = 1.0$ Hz; (a06) $A_x = 0.1$, $f_x = 3.0$ Hz; (a07) $A_x = 0.2$, $f_x = 3.0$ Hz; (a08) $A_x = 0.3$, $f_x = 3.0$ Hz; (a09) $A_x = 0.4$, $f_x = 3.0$ Hz; (a10) $A_x = 0.5$, $f_x = 3.0$ Hz; (a11) $A_x = 0.1$, $f_x = 5.0$ Hz; (a12) $A_x = 0.2$, $f_x = 5.0$ Hz; (a13) $A_x = 0.3$, $f_x = 5.0$ Hz; (a14) $A_x = 0.4$, $f_x = 5.0$ Hz; (a15) $A_x = 0.5$, $f_x = 5.0$ Hz; (a16) $A_x = 0.1$, $f_x = 7.0$ Hz; (a17) $A_x = 0.2$, $f_x = 7.0$ Hz; (a18) $A_x = 0.3$, $f_x = 7.0$ Hz; (a19) $A_x = 0.4$, $f_x = 7.0$ Hz; and (a20) $A_x = 0.5$, $f_x = 7.0$ Hz.

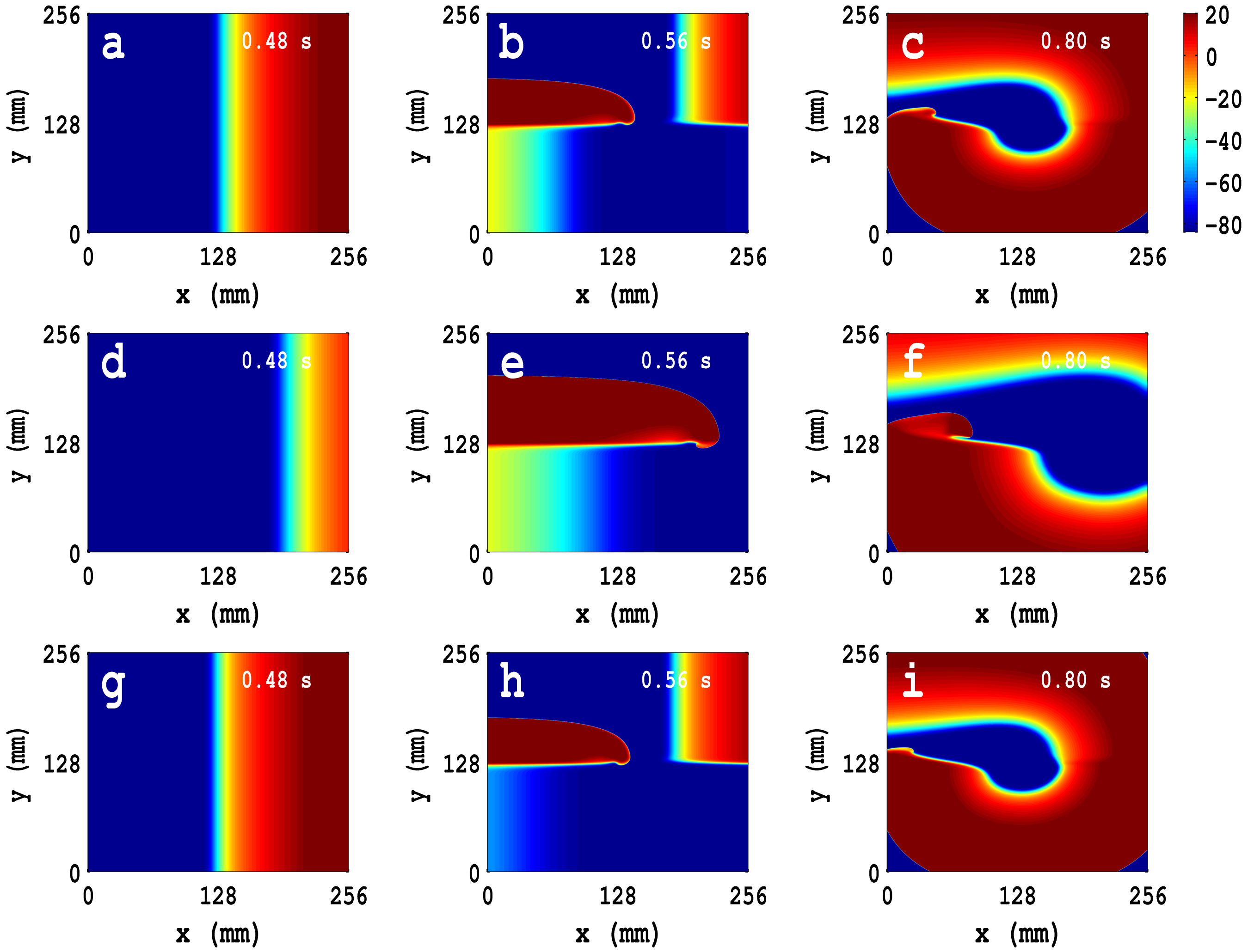

Figure S5. Initiation of spiral waves in the TP06 model by the S1-S2 cross field protocol: Pseudocolor plots of the transmembrane potential V_m showing the time evolution of spiral waves for initial conditions (see Table. 1 in the main paper) *IC1* ((a)-(c)), *IC2* ((d)-(f)), and *IC3* ((g)-(i)).

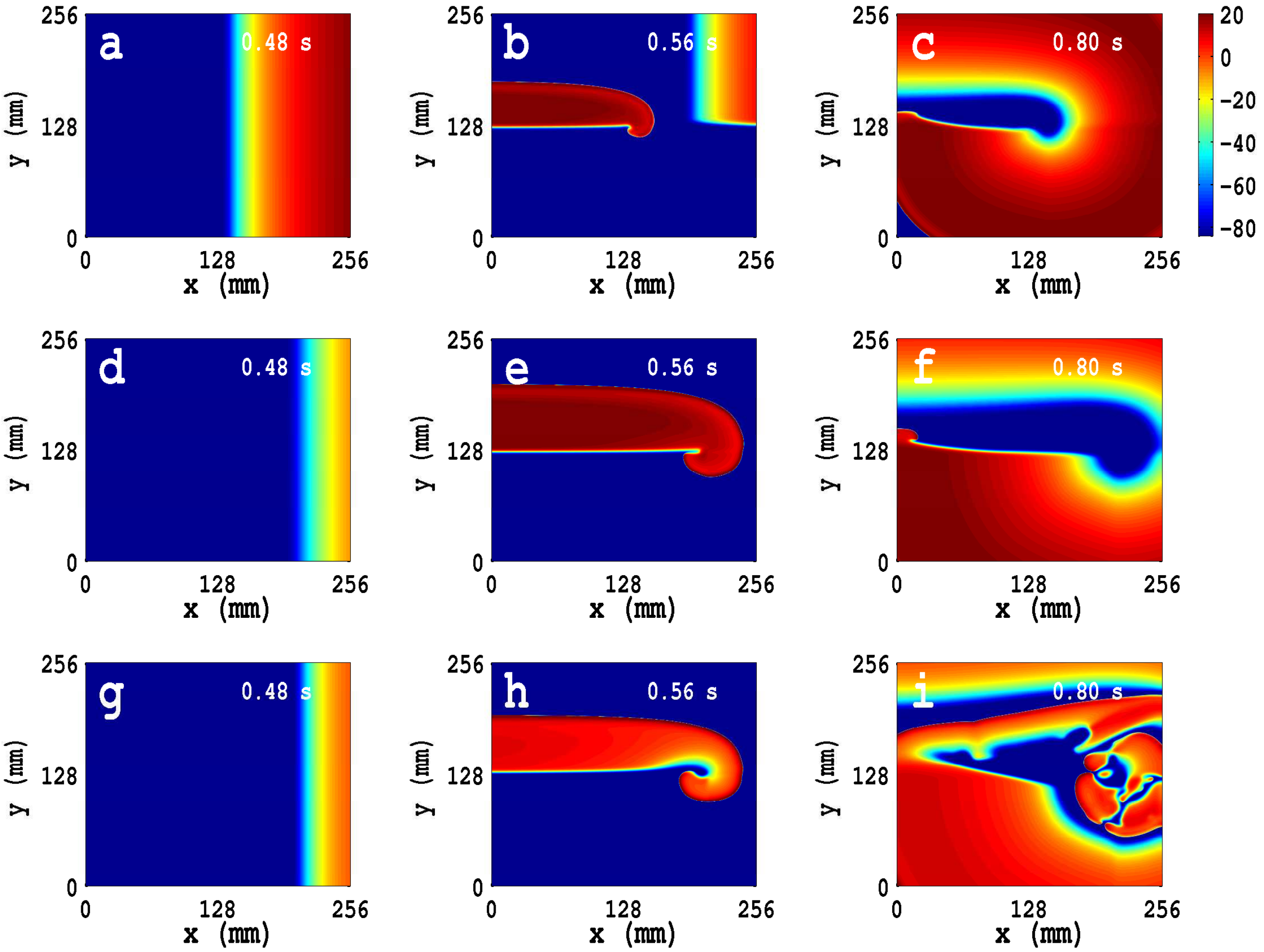

Figure S6. Initiation of spiral waves in the TNNP04 model by the S1-S2 cross field protocol: Pseudocolor plots of the transmembrane potential V_m showing the time evolution of spiral waves for initial conditions *IC1* ((a)-(c)), *IC2* ((d)-(f)), and *IC3* ((g)-(i)).

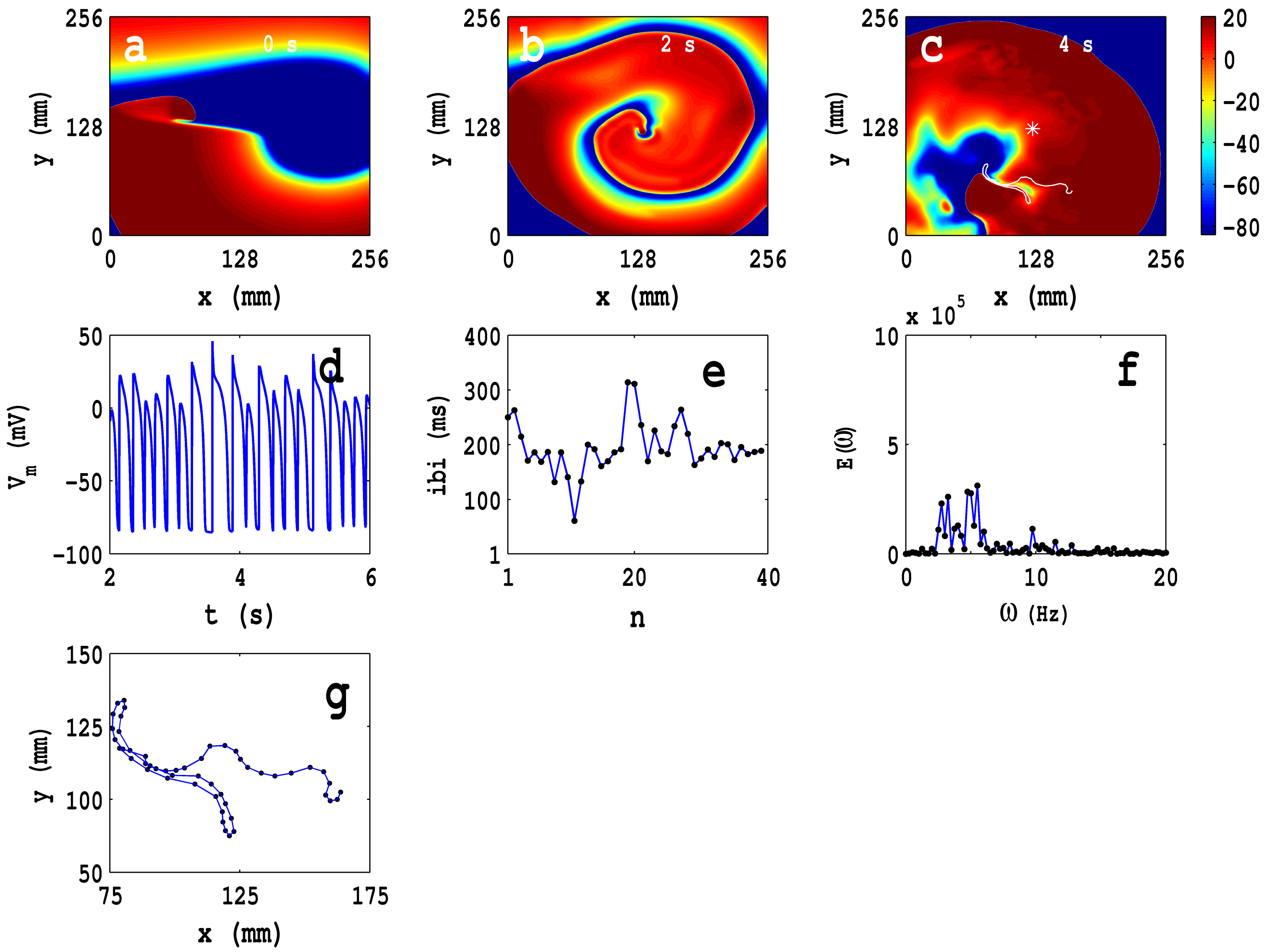

Figure S7. Spatiotemporal evolution of V_m for the initial condition *IC2* for the TP06 model in the absence of PD: (a)-(g) show, for the initial condition *IC2* in the absence of PD ; and the animation (b) in Video S2 shows the spatiotemporal evolution of V_m for this case. This animation, the pseudocolor plots of V_m ((a)-(c)), the representative local time series of V_m (d), the plot of the ibi (e), the power spectrum $E(\omega)$ (f), and the spiral-tip trajectory (the white curve (c) and the blue one in (g)) show that the initial condition *IC2* leads to spatiotemporal chaos and spiral turbulence (ST) with a single spiral meandering chaotically in the simulation domain.

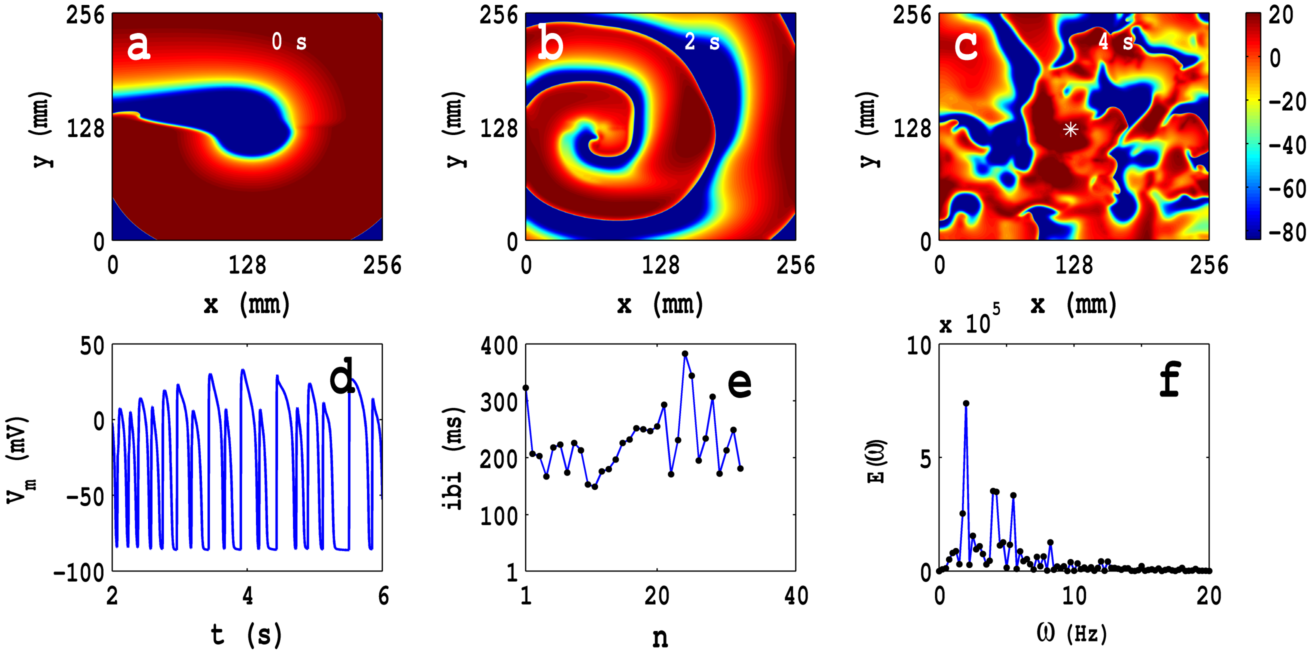

Figure S8. Spatiotemporal evolution of V_m for the initial condition *IC3* for the TP06 model in the absence of PD: (a)-(f) show, for the initial condition *IC3* in the absence of PD, the exact analogs of Figs. S7(a)-(f); and the animation (c) in Video S2 shows the spatiotemporal evolution of V_m for this case. This animation, the pseudocolor plots of V_m ((a)-(c)), the representative local time series of V_m (d), the plot of the ibi (e), and the power spectrum $E(\omega)$ (f) show that the initial condition *IC3* leads to spatiotemporal chaos and spiral turbulence (ST) with broken spirals in the simulation domain; the life-span of a given spiral-wave segment is small so we do not track the trajectories of spiral tips in this case.

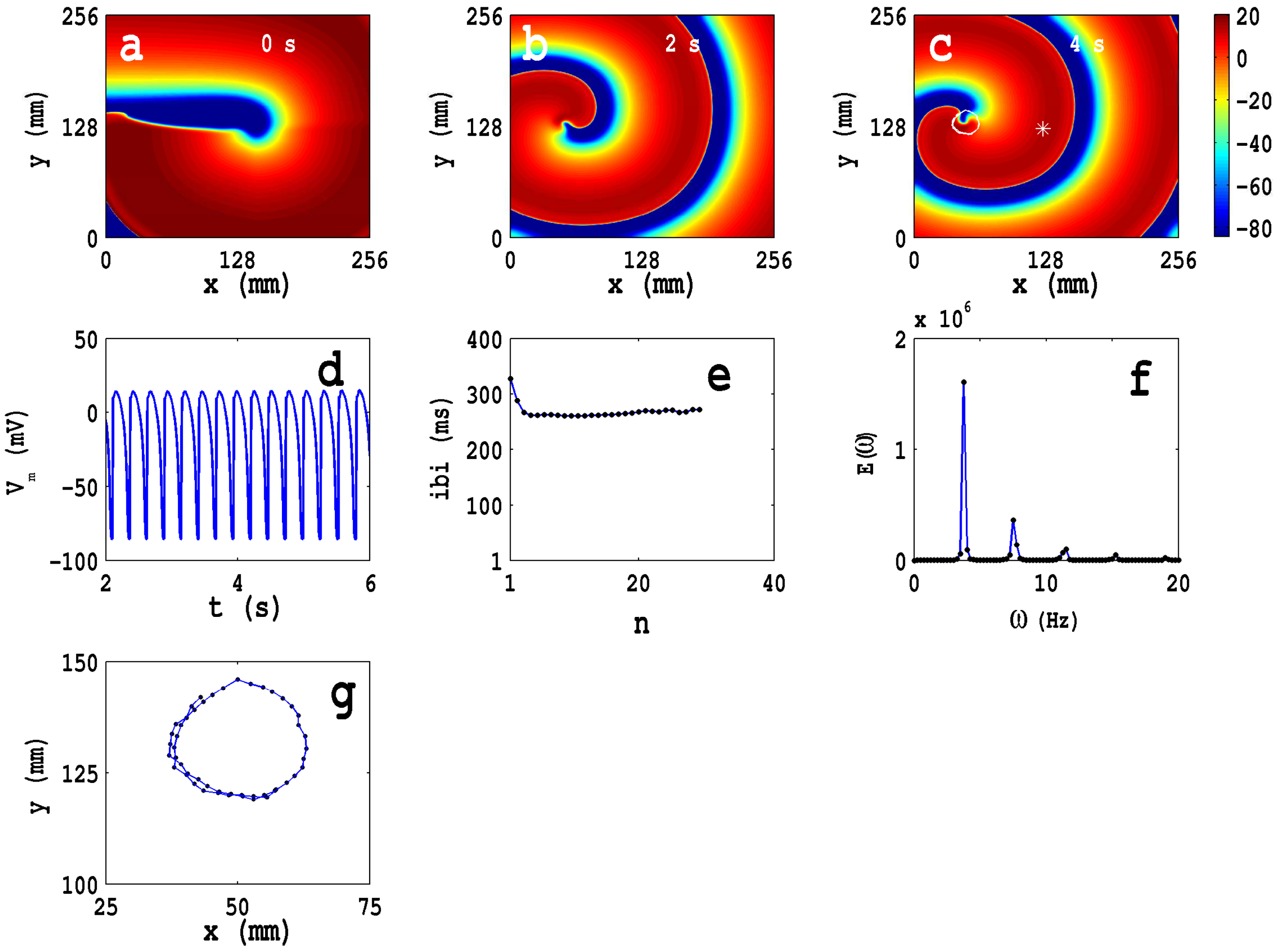

Figure S9. Spatiotemporal evolution of V_m for the initial condition *IC1* for the TNNP04 model in the absence of PD: (a)-(c)Pseudocolor plots of V_m at times $t = 0$ s, $t = 2$ s, and $t = 4$ s, respectively, showing the evolution towards a state with a rotating spiral (RS); the animation (d) in Video S2 shows the spatiotemporal evolution of V_m for this case. (d) The local time series of $V_m(x, y, t)$, from the representative point ($x = 125$ mm, $y = 125$ mm) (the asterisk in (c)) for 2 s $\leq t \leq$ 6 s; (e) a plot of the inter beat interval (ibi), which we obtain from this time series, of length 4×10^5 iterations; (f) the power spectrum $E(\omega)$, obtained from the local time series of (d), with discrete peaks at the fundamental frequency $\omega_f \simeq 3.75$ Hz and its harmonics. The spiral-tip trajectory traces a roughly circular path, with radius $l_c \simeq 25$ mm, which is shown, for 3.6 s $\leq t \leq$ 4 s, by the white line that has been superimposed on the pseudocolor plot of V_m in (c); an expanded version of this path is shown in (g).

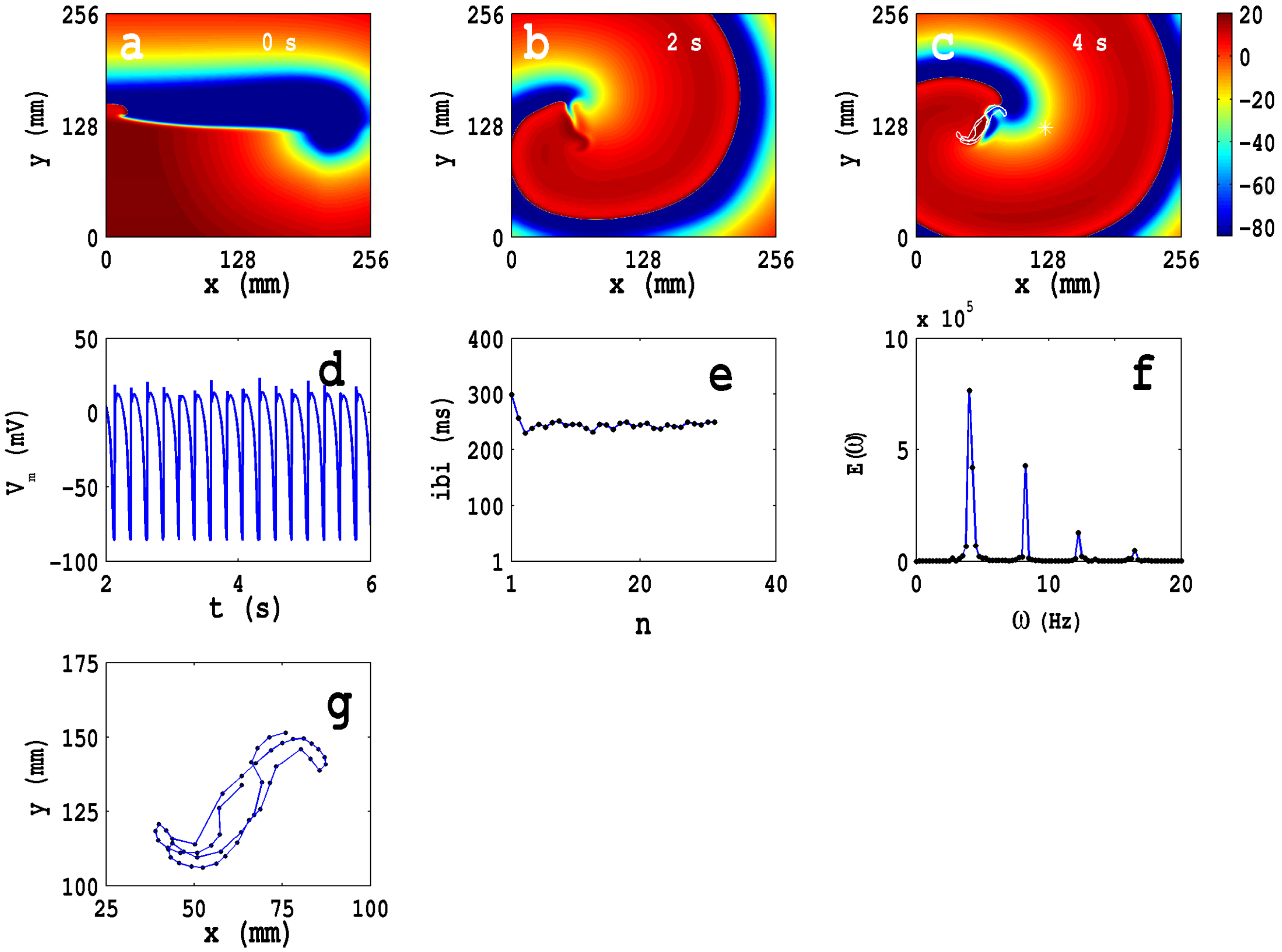

Figure S10. Spatiotemporal evolution of V_m for the initial condition *IC2* for the TNNP04 model in the absence of PD: (a)-(g) show, for the initial condition *IC2* in the absence of PD, the exact analogs of Figs. S9(a)-(g); and the animation (e) in Video S2 shows the spatiotemporal evolution of V_m for this case. (d) The local time series of $V_m(x, y, t)$, from the representative point (x = 125 mm, y = 125 mm) (the asterisk in (c)) for 2 s ≤ t ≤ 6 s; (e) a plot of the inter beat interval (ibi), which we obtain from this time series, of length 4×10^5 iterations; (f) the power spectrum $E(\omega)$, obtained from the local time series of (d), with two principal frequencies, ω_1 = 4 Hz and ω_2 = 8.25 Hz. The spiral-tip trajectory traces a Z-type path, with linear extents $l_c \simeq 43$ mm, which is shown, for 3.6 s ≤ t ≤ 4 s, by the white line that has been superimposed on the pseudocolor plot of V_m in (c); an expanded version of this path is shown in (g). The local time series, the oscillating ibi, and more than one principal frequency in the power spectrum show that the initial condition *IC2* leads to a quasiperiodic temporal evolution.

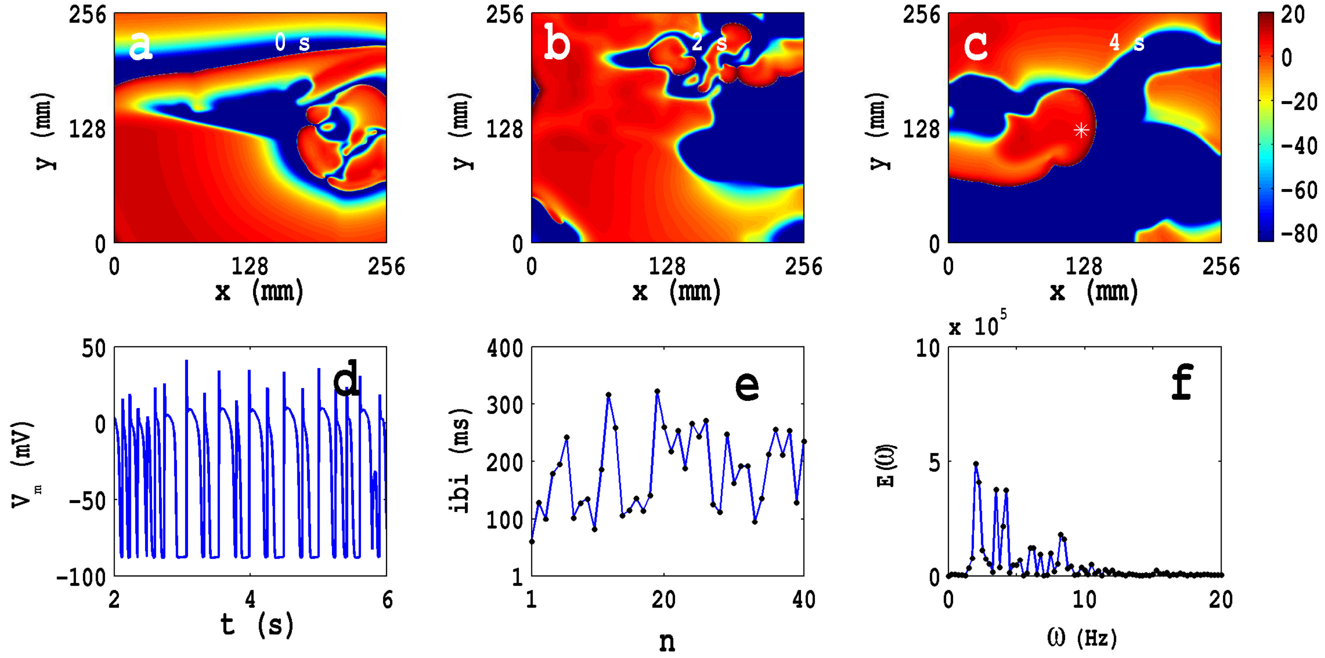

Figure S11. Spatiotemporal evolution of V_m for the initial condition *IC3* for the TNNP04 model in the absence of PD: (a)-(f) show, for the initial condition *IC3* in the absence of PD, the exact analogs of Figs. S9(a)-(f); and the animation (f) in Video S2 shows the spatiotemporal evolution of V_m for this case. This animation, the pseudocolor plots of V_m ((a)-(c)), the representative local time series of V_m (d), the plot of the ibi (e), and the power spectrum $E(\omega)$ (f) show that the initial condition *IC3* leads to spatiotemporal chaos and spiral turbulence (ST) with broken spirals in the simulation domain.

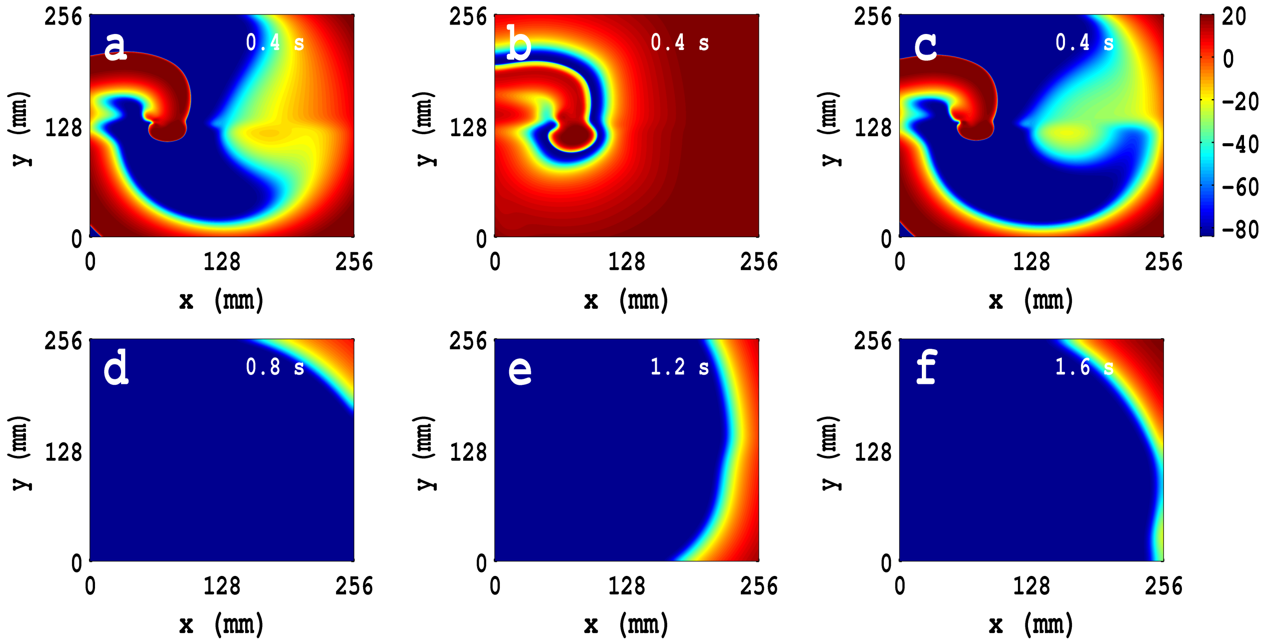

Figure S12. Pseudocolor plots of the transmembrane potential V_m for the TP06 model, with PD, illustrating spiral-wave absorption: Pseudocolor plots of V_m, for (a)-(c) $t = 0.4$ s, and (d), (e), and (f), for $t = 0.8$ s, $t = 1.2$ s, and $t = 1.6$ s, respectively, for three representative cases of spiral-wave absorption with the following PDs: (a) and (d) $A_x = A_y = 0.4$, $f_x = f_y = 3.0$ Hz, (b) and (e) $A_x = A_y = 0.5$, $f_x = f_y = 1.0$ Hz, and (c) and (f) $A_x = A_y = 0.5$, $f_x = f_y = 3.0$ Hz; the spatiotemporal evolution of V_m for these cases is shown, respectively, in the animations (b4), (a5), and (b5) in Video S3 for the time interval $0 \text{ s} \leq t \leq 4$ s.

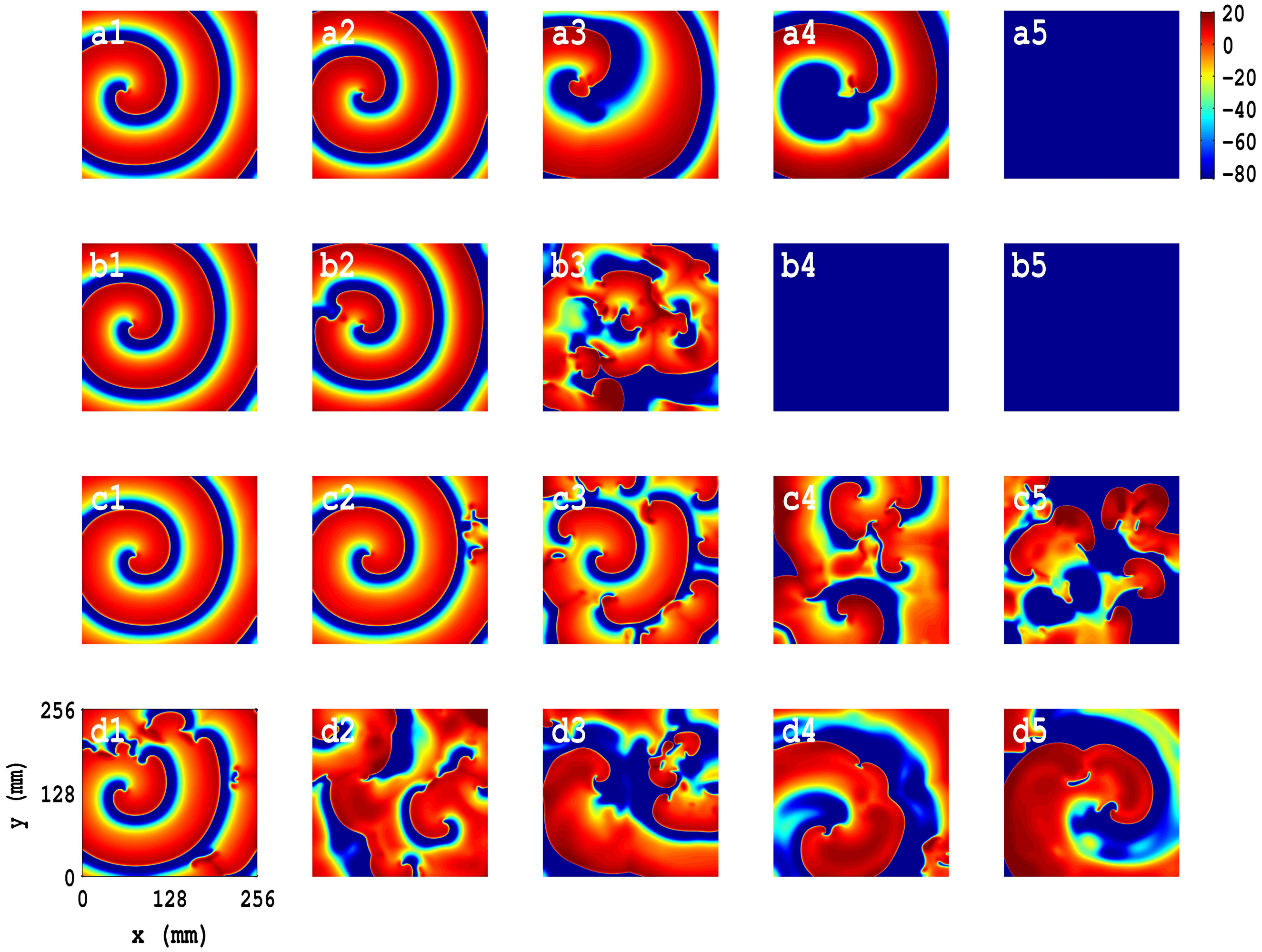

Figure S13. Illustrations of the rich variety of spatiotemporal patterns for the TP06 model, with PD and the initial condition $IC1$: Representative pseudocolor plots of V_m with the initial condition $IC1$ (Fig. S5(c)) and the parameter sets given in Table. 1 (main paper). The animations in Video S3 show the spatiotemporal evolution of V_m for these cases in the time interval $0\ \text{s} \leq t \leq 4\ \text{s}$. To examine this evolution, we obtain the local time series of $V_m(x, y, t)$, from the representative points $(x = 125\ \text{mm}, y = 125\ \text{mm})$; these are shown in Fig. S14; from these local time series, we obtain the plots of the ibi (Fig. S15) and the power spectra (Fig. S16).

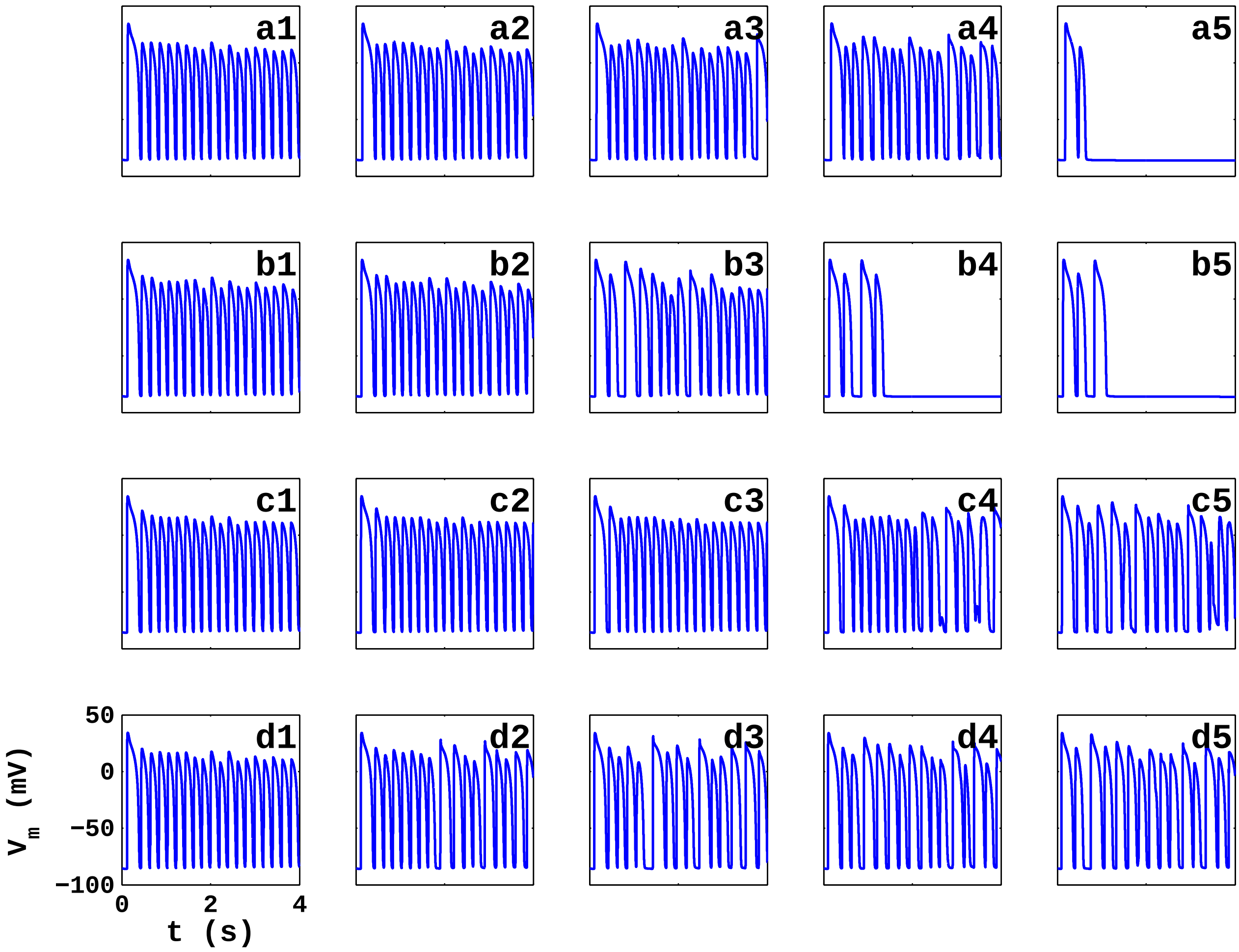

Figure S14. Plots of the local time series of $V_m(x, y, t)$, recorded from the representative points ($x = 125$ mm, $y = 125$ mm), in the TP06 model with the initial condition *IC1* and PD along both spatial directions; the spatiotemporal patterns of V_m are shown in Fig. S13.

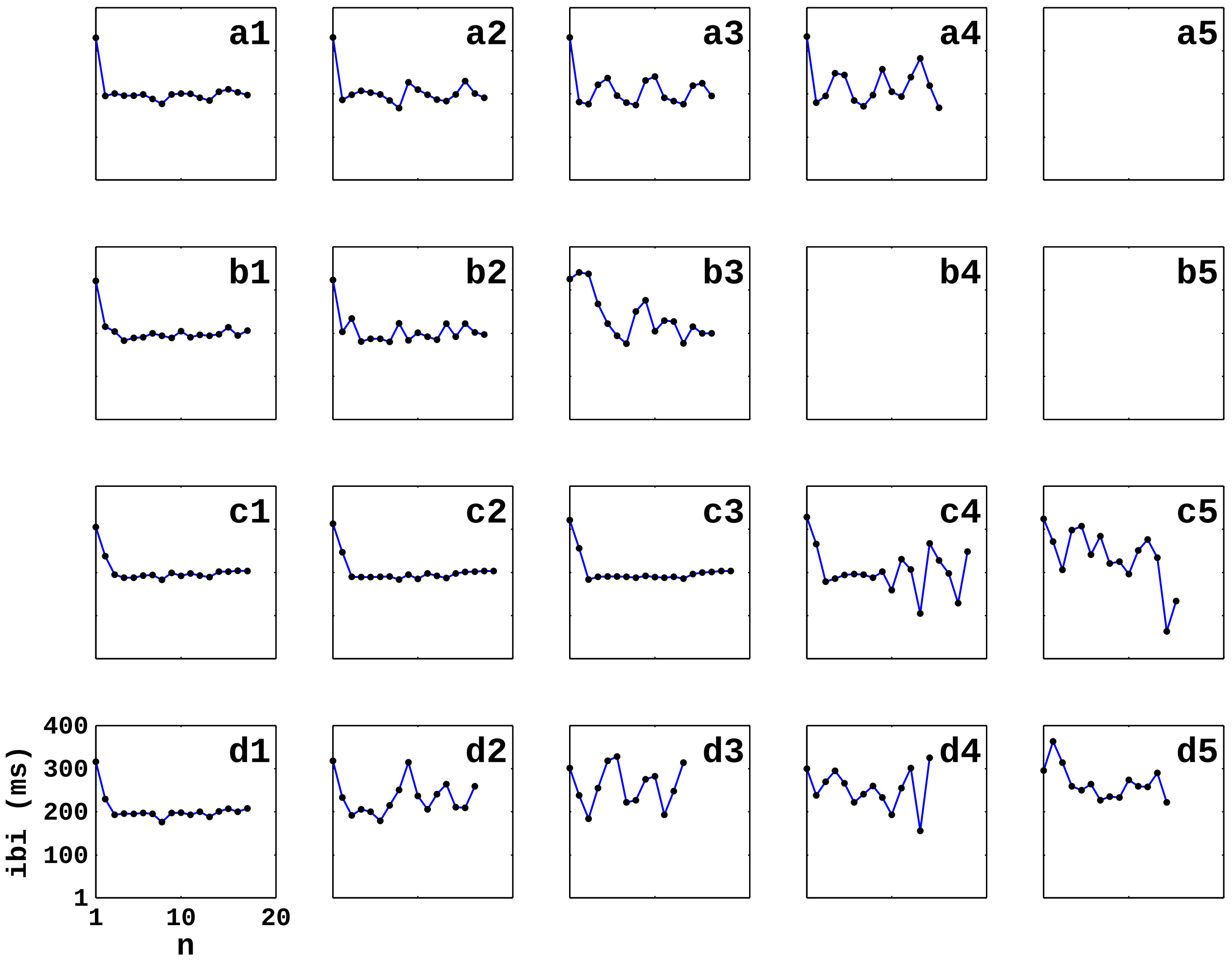

Figure S15. Plots of the ibi versus the beat number n that we obtain from the time series shown in Fig. S14.

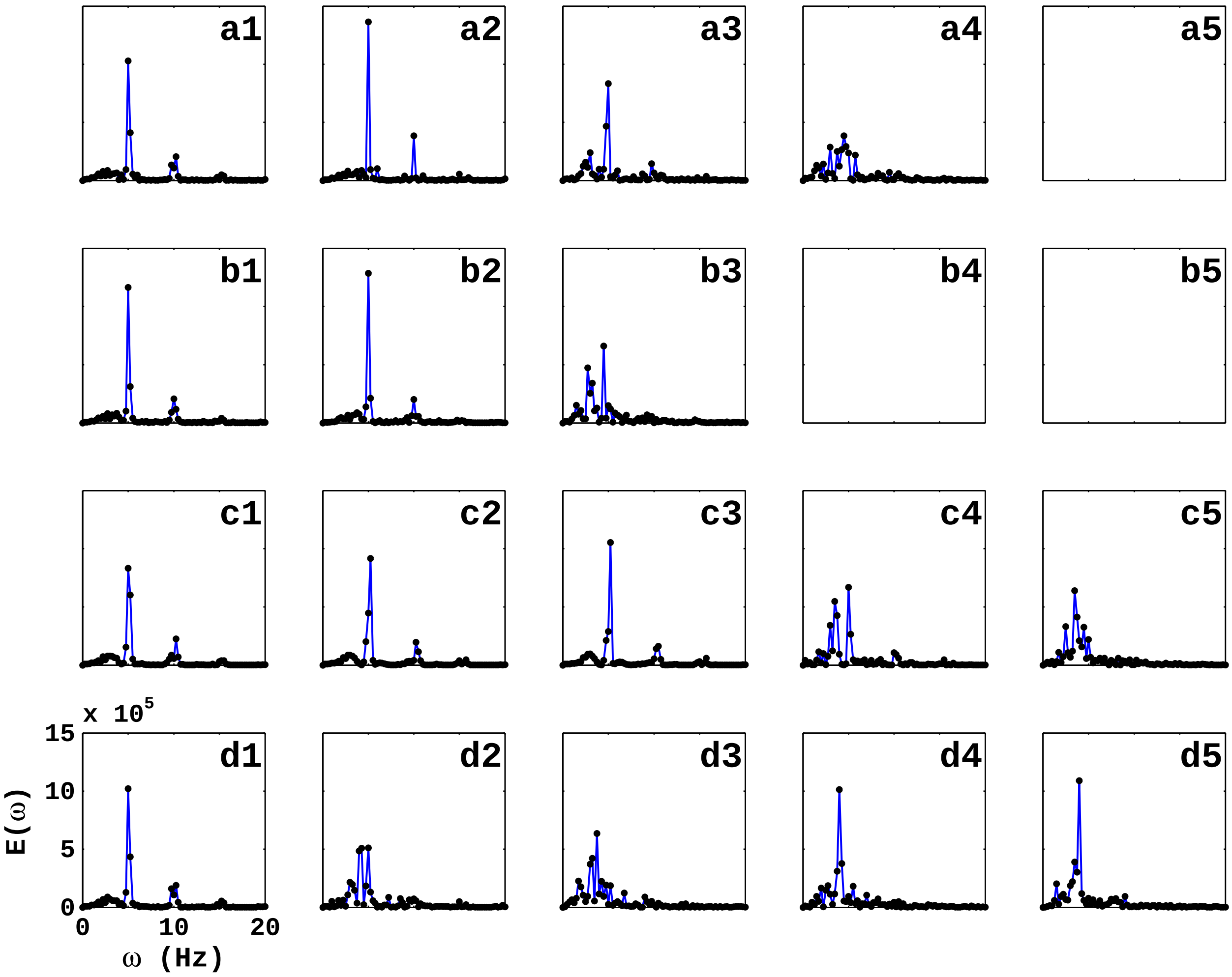

Figure S16. Plots of the power spectrum $E(\omega)$ of V_m obtained from a time series of length 2×10^5 for the data points in Fig. S14.

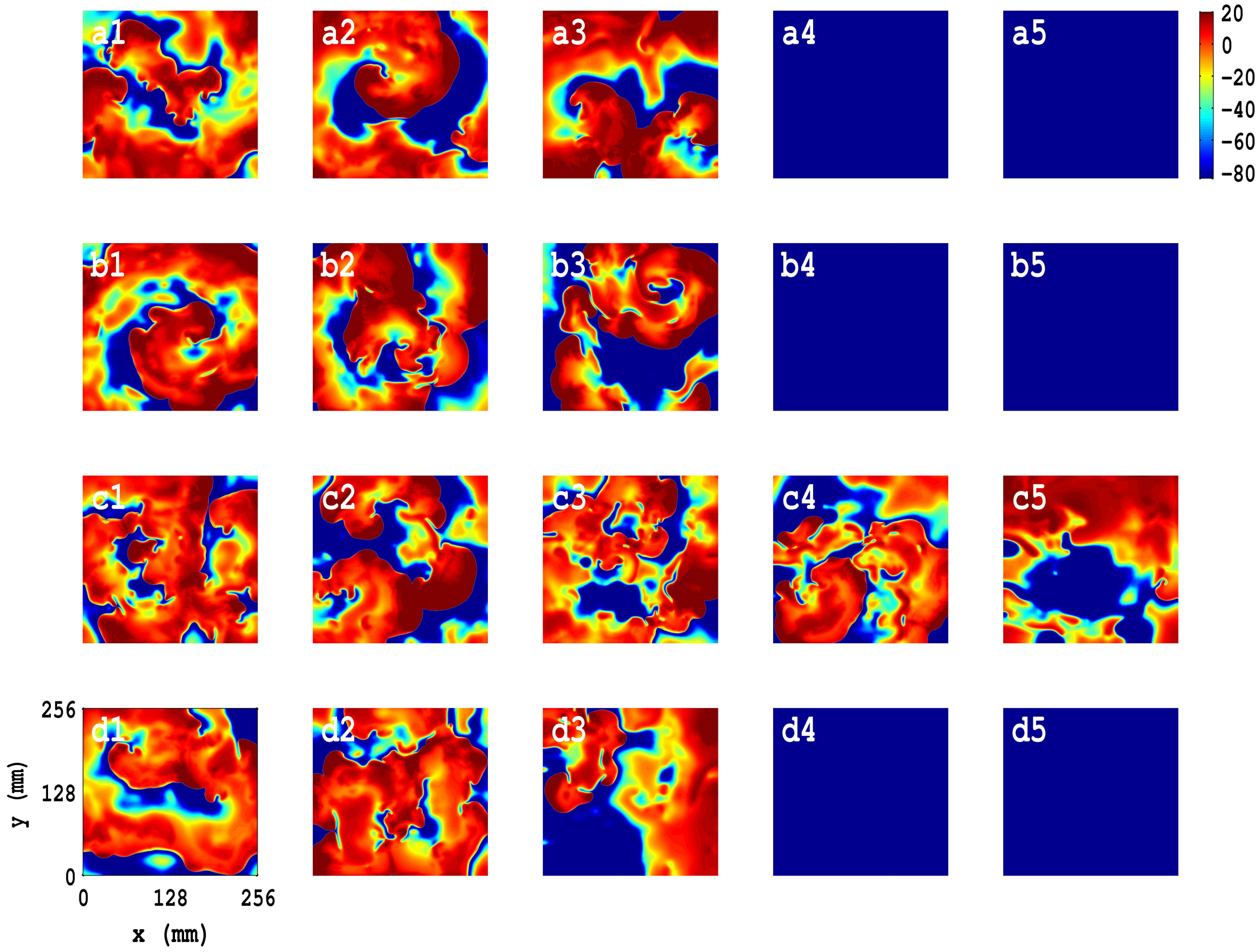

Figure S17. Illustrations of the rich variety of spatiotemporal patterns for the TP06 model, with PD and the initial condition $IC2$ (Fig. S5(f)): The analogs of the pseudocolor plots of V_m in Figs. S13 for the initial conditions $IC2$; the plots (a1)-(d5) here use the same PD parameters as their counterparts in Figs. S13; the Video S4 shows the spatiotemporal evolution of V_m for these cases for the time interval $0 \text{ s} \leq t \leq 4 \text{ s}$.

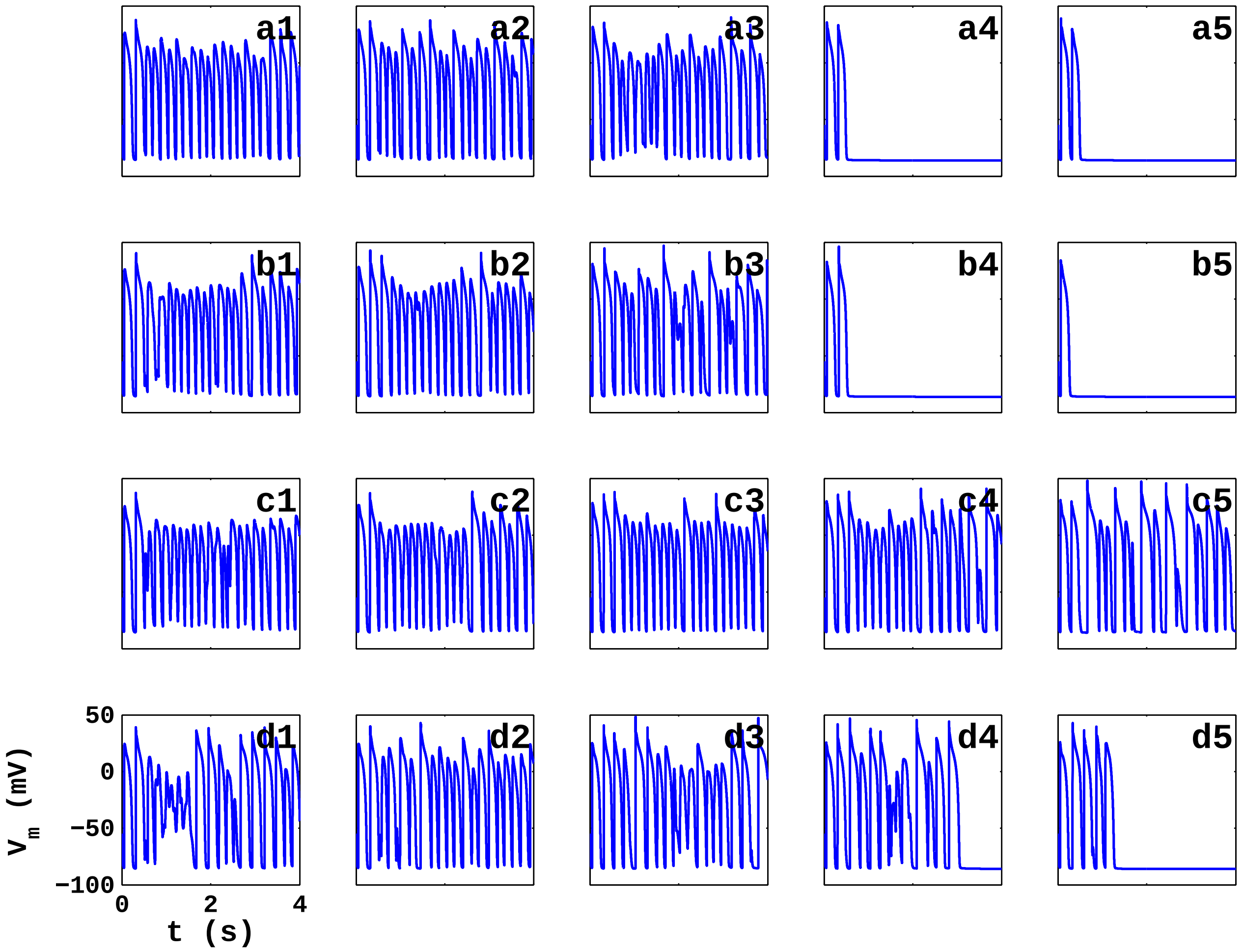

Figure S18. Plots of the local time series of $V_m(x, y, t)$, recorded from the representative points ($x = 125$ mm, $y = 125$ mm), in the TP06 model with the initial condition *IC2* and PD along both spatial directions; the spatiotemporal patterns of V_m are shown in Fig. S17.

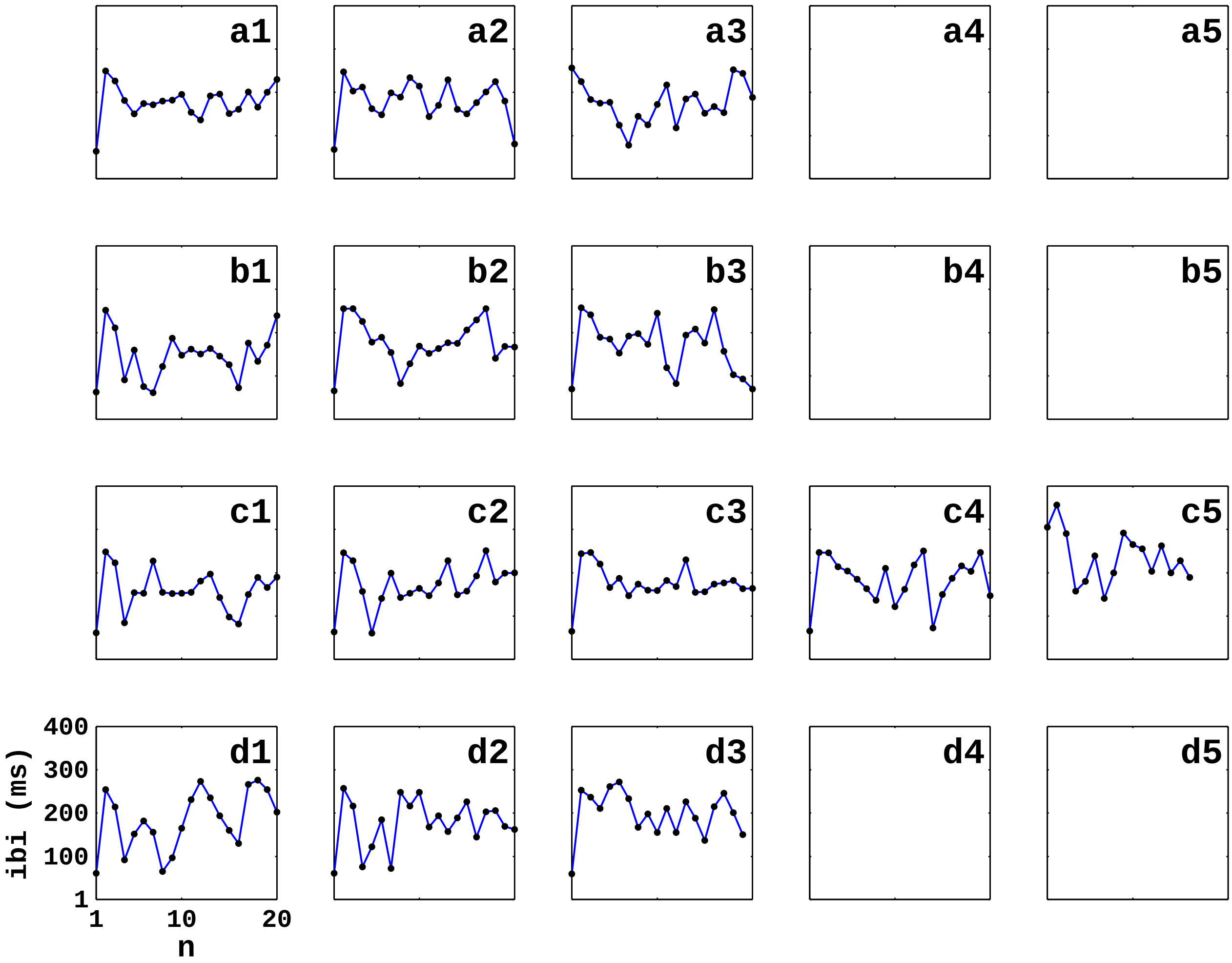

Figure S19. Plots of the ibi versus the beat number n that we obtain from the time series shown in Fig. S18.

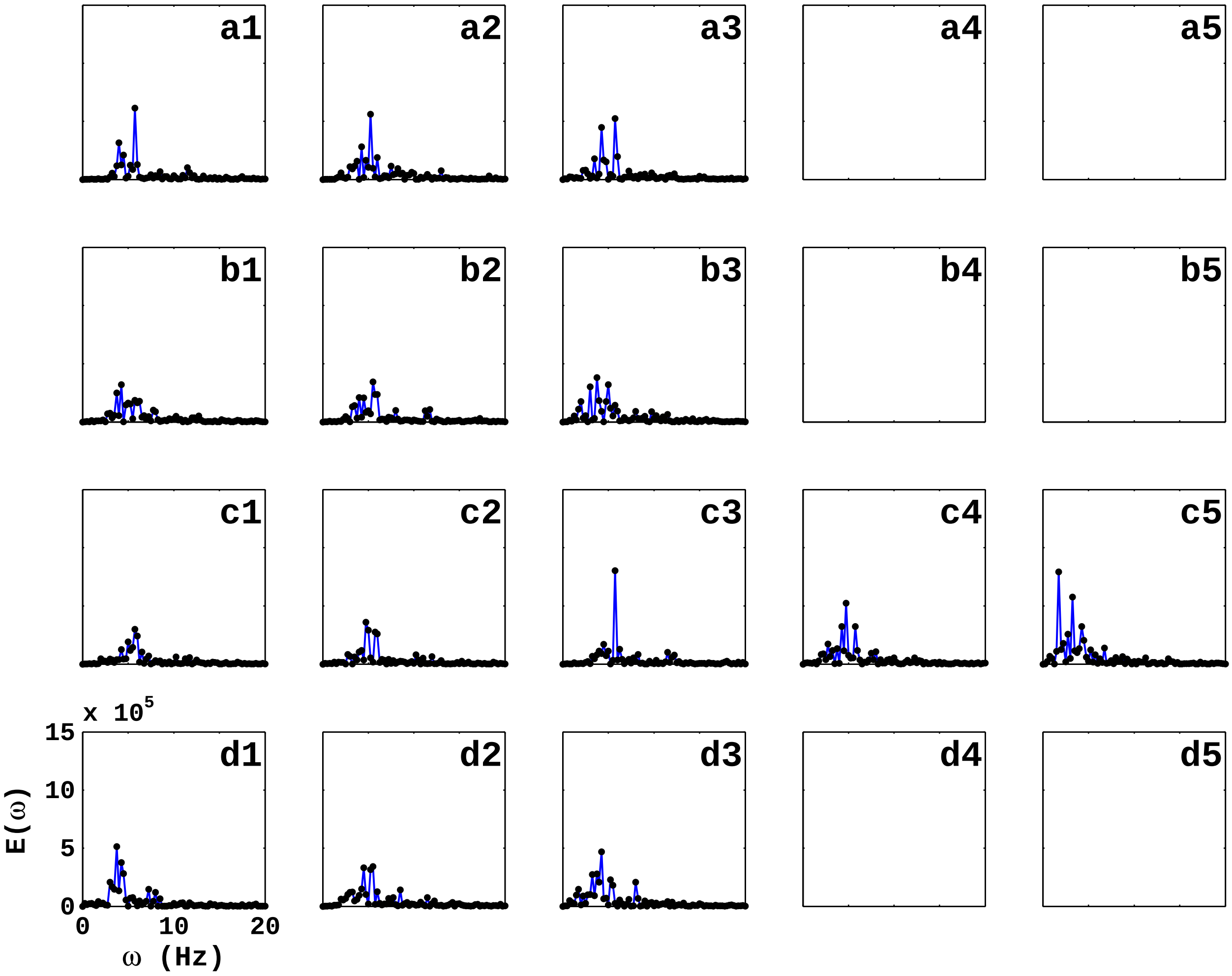

Figure S20. Plots of the power spectrum $E(\omega)$ of V_m obtained from a time series of length 2×10^5 for the data points in Fig. S18.

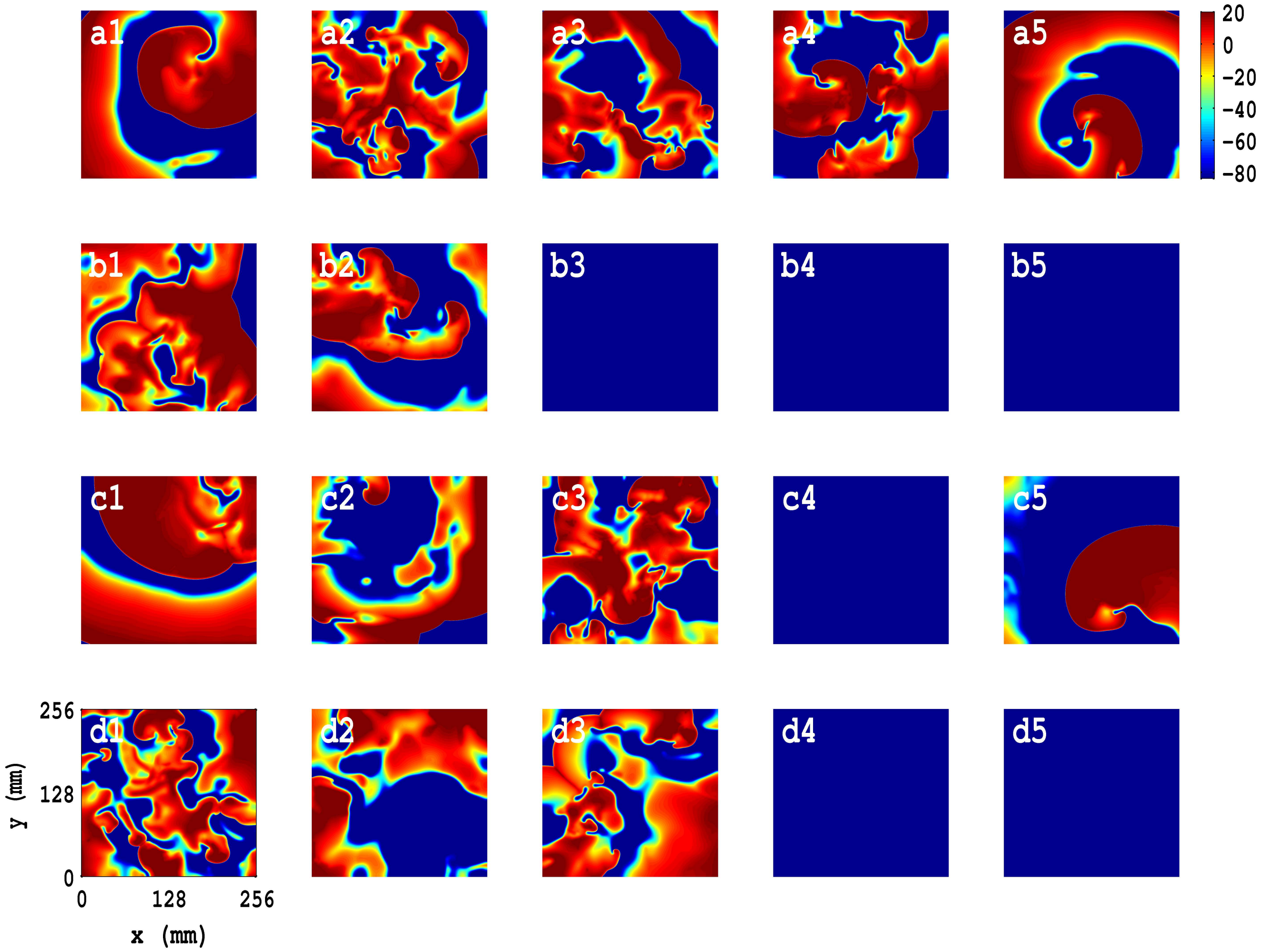

Figure S21. Illustrations of the rich variety of spatiotemporal patterns for the TP06 model, with PD and the initial condition *IC3* (Fig. S5(i)): The analogs of the pseudocolor plots of V_m in Figs. S13 for the initial conditions *IC3*; the plots (a1)-(d5) here use the same PD parameters as their counterparts in Figs. S13; the Video S5 shows the spatiotemporal evolution of V_m for these cases for the time interval $0 \text{ s} \leq t \leq 4 \text{ s}$.

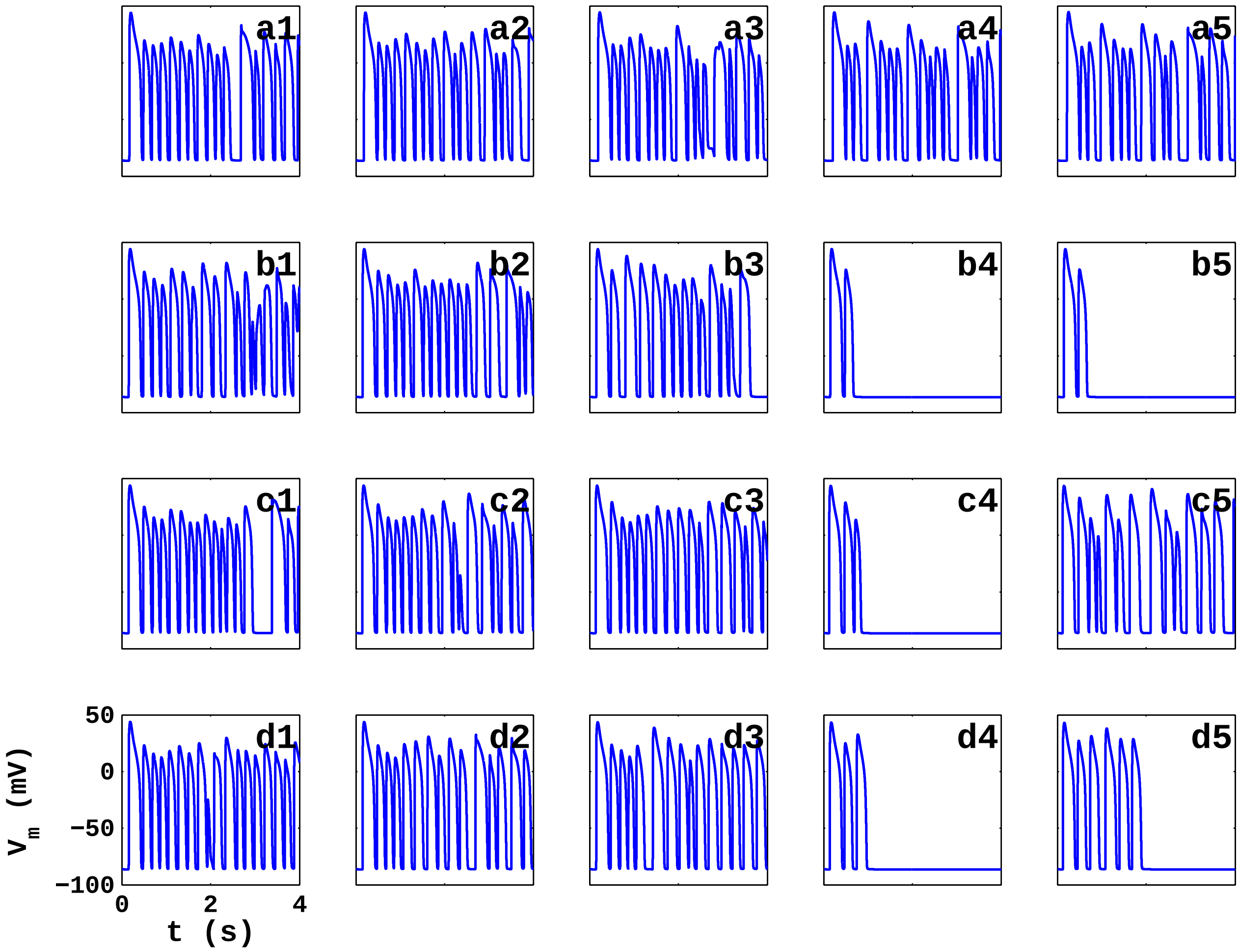

Figure S22. Plots of the local time series of $V_m(x, y, t)$, recorded from the representative points ($x = 125$ mm, $y = 125$ mm), in the TP06 model with the initial condition *IC3* and PD along both spatial directions; the spatiotemporal patterns of V_m are shown in Fig. 13 in the main text.

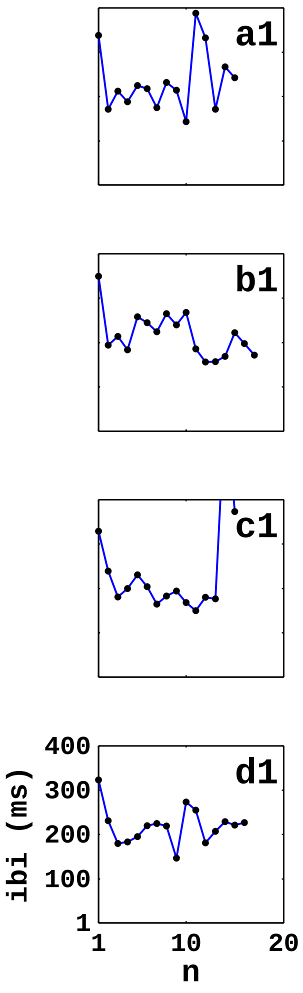

Figure S23. Plots of the ibi versus the beat number n that we obtain from the time series shown in Fig. S22.

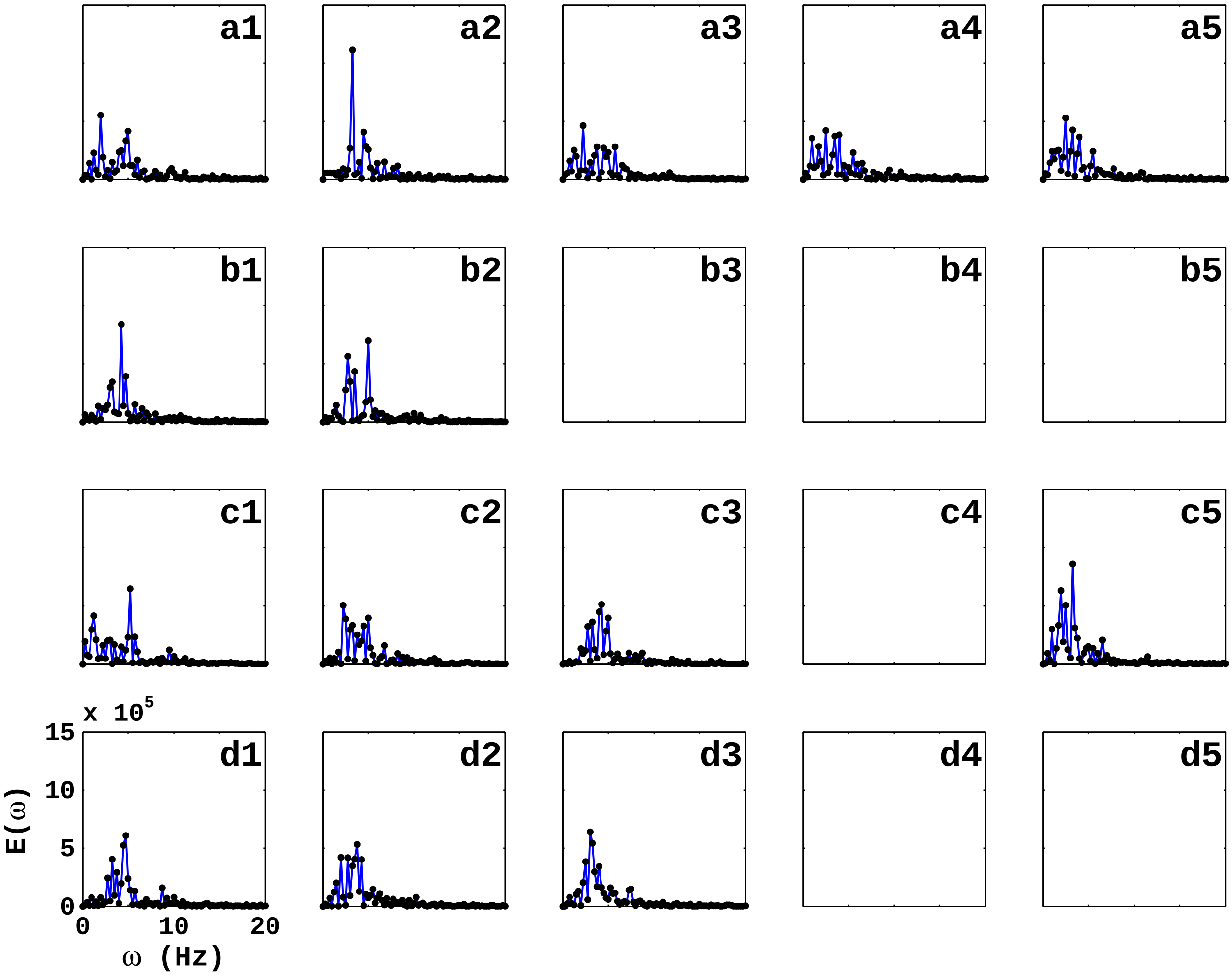

Figure S24. Plots of the power spectrum $E(\omega)$ of V_m obtained from a time series of length 2×10^5 for the data points in Fig. S22.

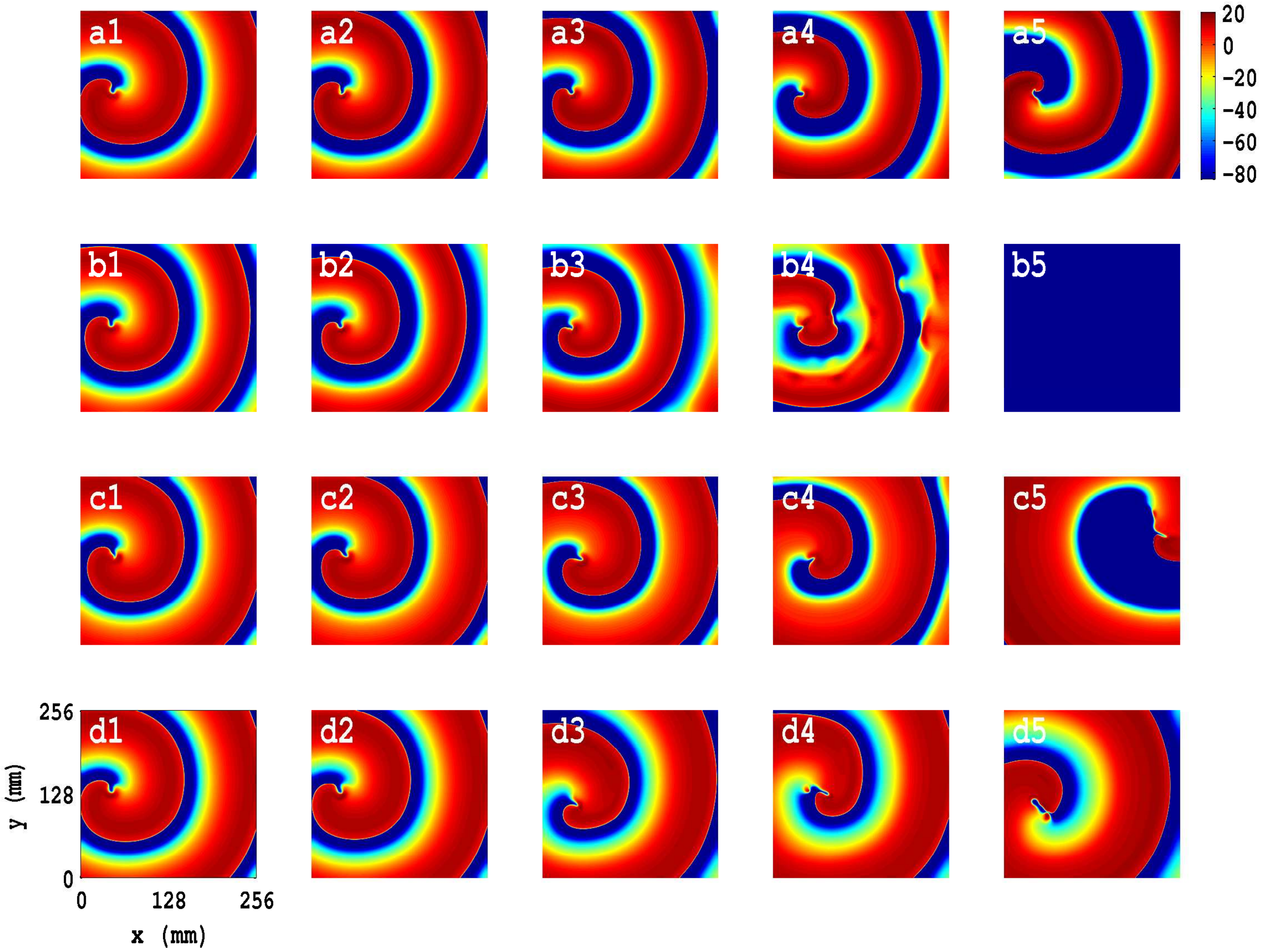

Figure S25. Illustrations of the rich variety of spatiotemporal patterns for the TNNP04 model, with PD along both x and y directions and the initial condition *IC1*: Representative pseudocolor plots of V_m with the initial condition *IC1* (Fig. S6(c)) and the parameter sets given in Table. 1 (main paper); the animations in Video S6 show the spatiotemporal evolution of V_m for these cases in the time interval $0 \text{ s} \leq t \leq 4 \text{ s}$.

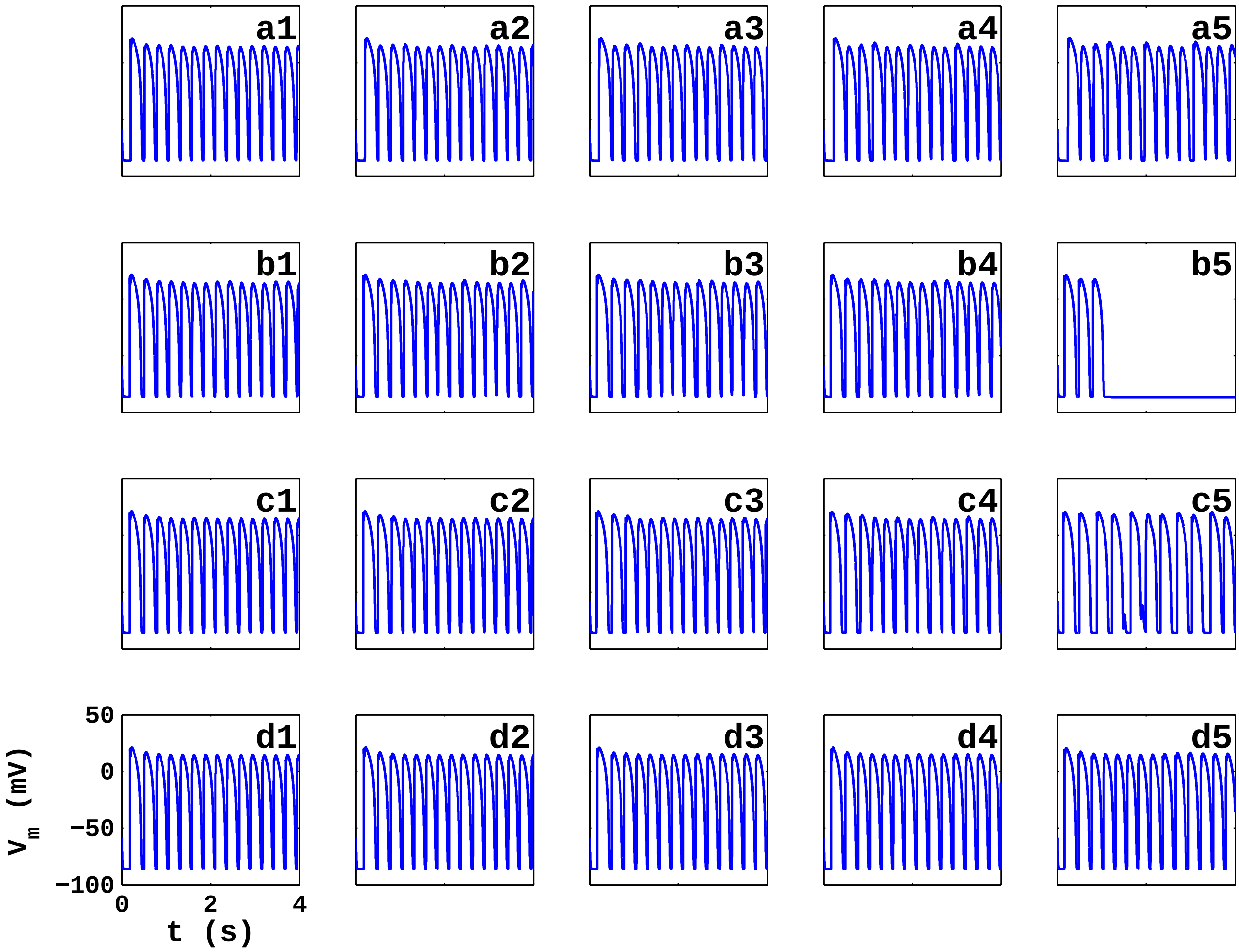

Figure S26. Plots of the local time series of $V_m(x, y, t)$, recorded from the representative points (x = 125 mm, y = 125 mm), in the TNNP04 model with the initial condition *IC1* and PD along both spatial directions; the spatiotemporal patterns of V_m are shown in Fig. S25.

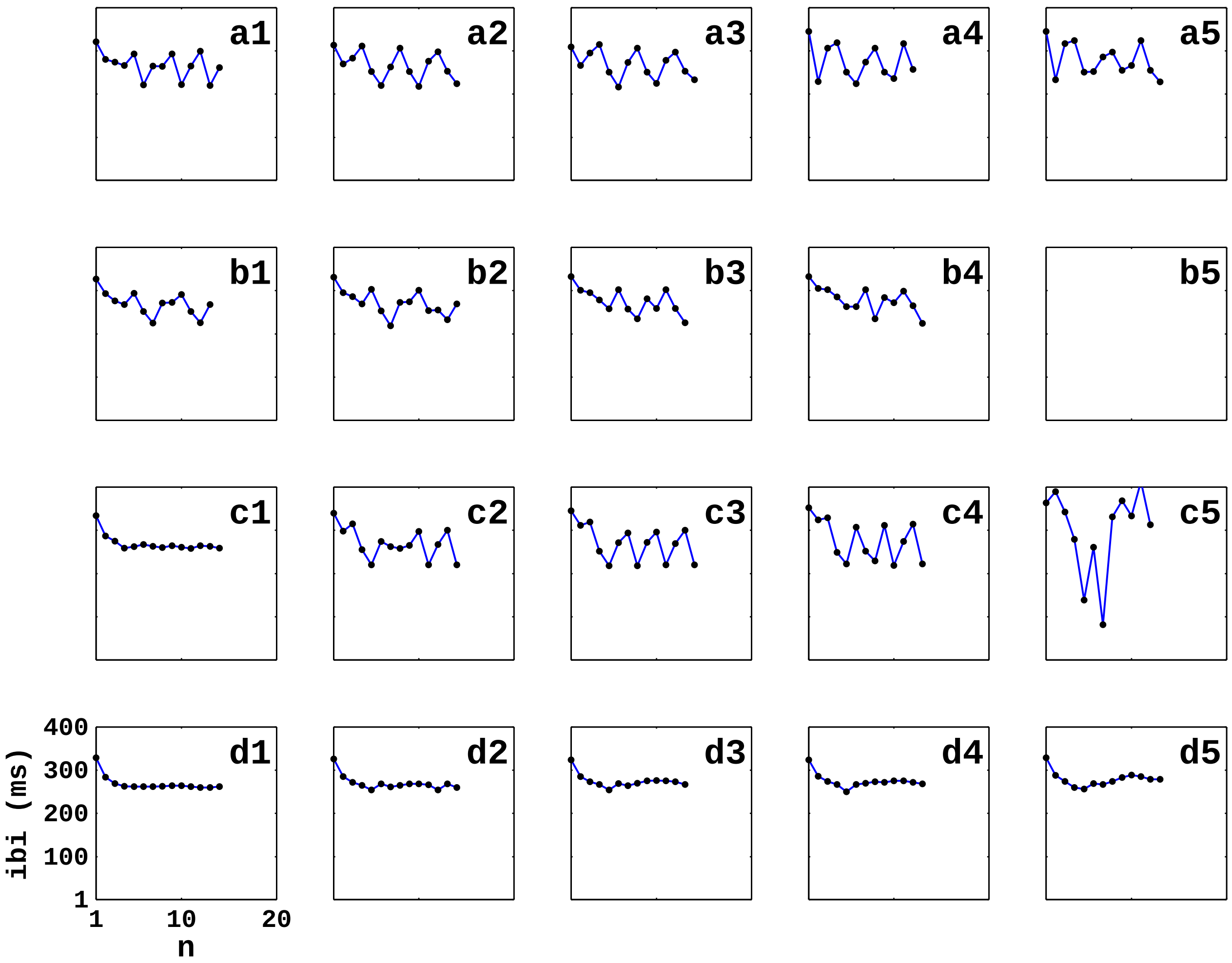

Figure S27. Plots of the ibi versus the beat number n that we obtain from the time series shown in Fig. S26.

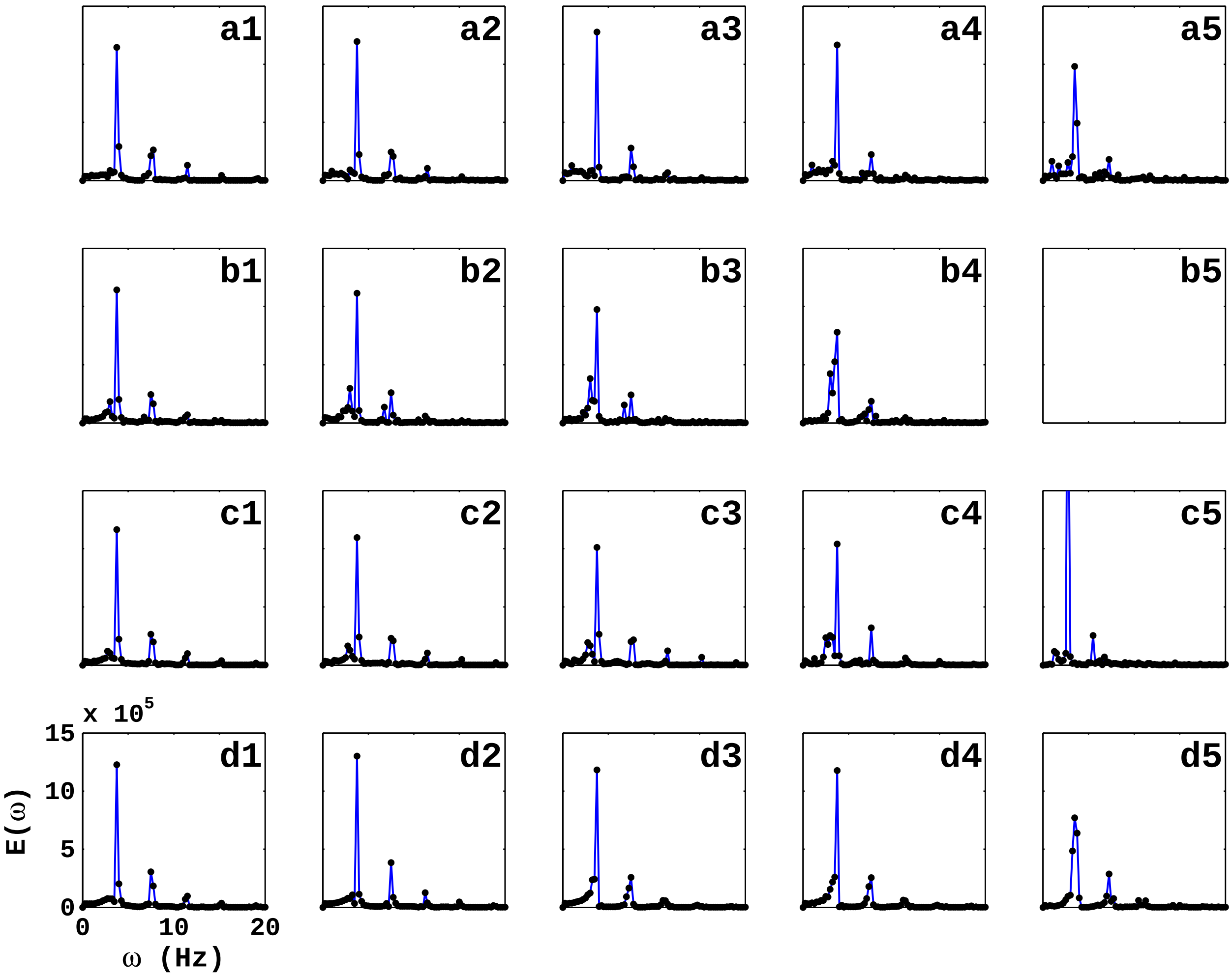

Figure S28. Plots of the power spectrum $E(\omega)$ of V_m obtained from a time series of length 2×10^5 for the data points in Fig. S26.

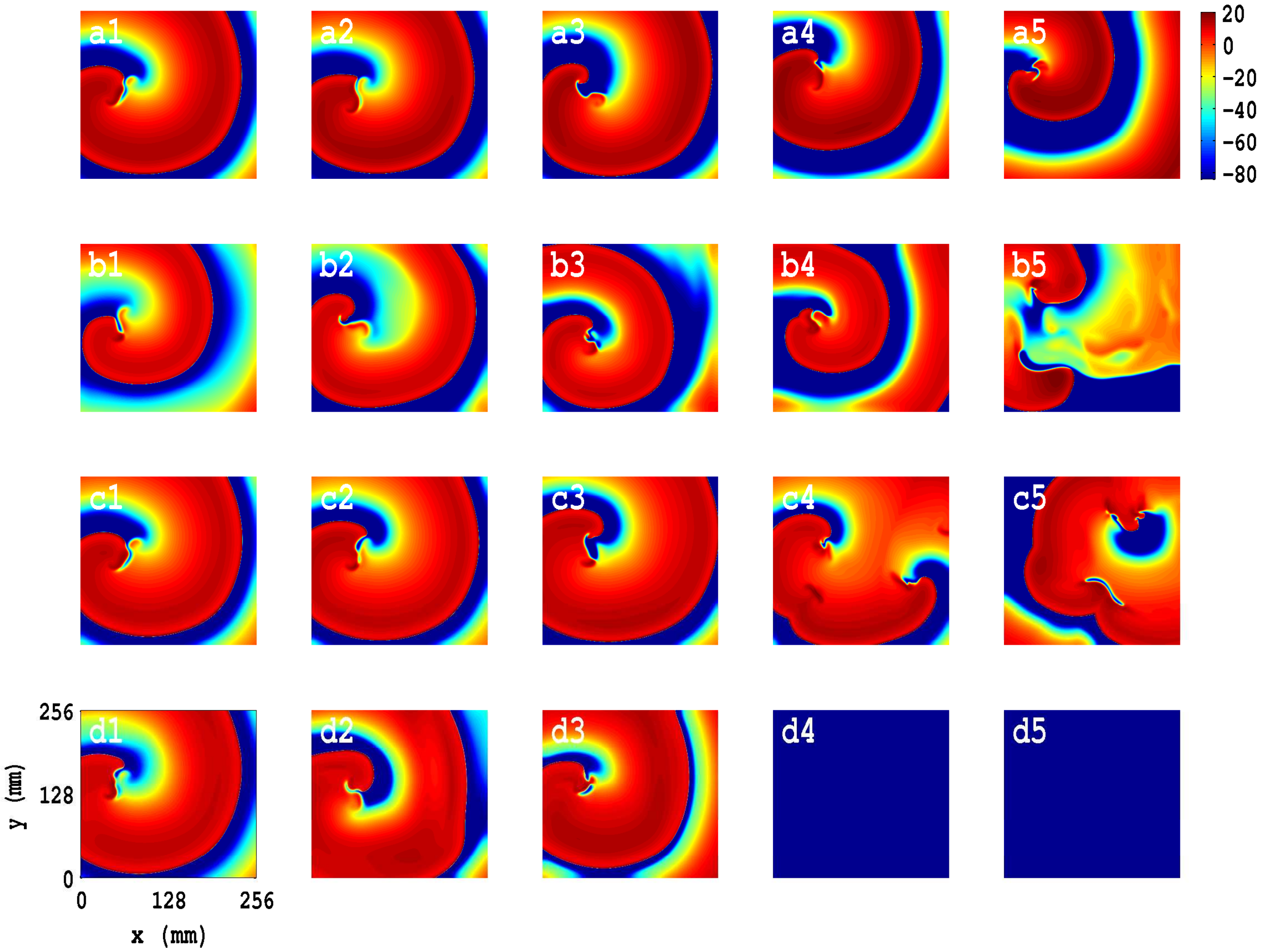

Figure S29. Illustrations of the rich variety of spatiotemporal patterns for the TNNP04 model, with PD along both x and y directions and the initial condition *IC2* (Fig. S6(f)): The analogs of the pseudocolor plots of V_m in Fig. S25 for the initial conditions *IC2*; the plots (a1)-(d5) here use the same PD parameters as their counterparts in Fig. S25; the Video S7 shows the spatiotemporal evolution of V_m for these cases for the time interval $0 \text{ s} \leq t \leq 4$ s.

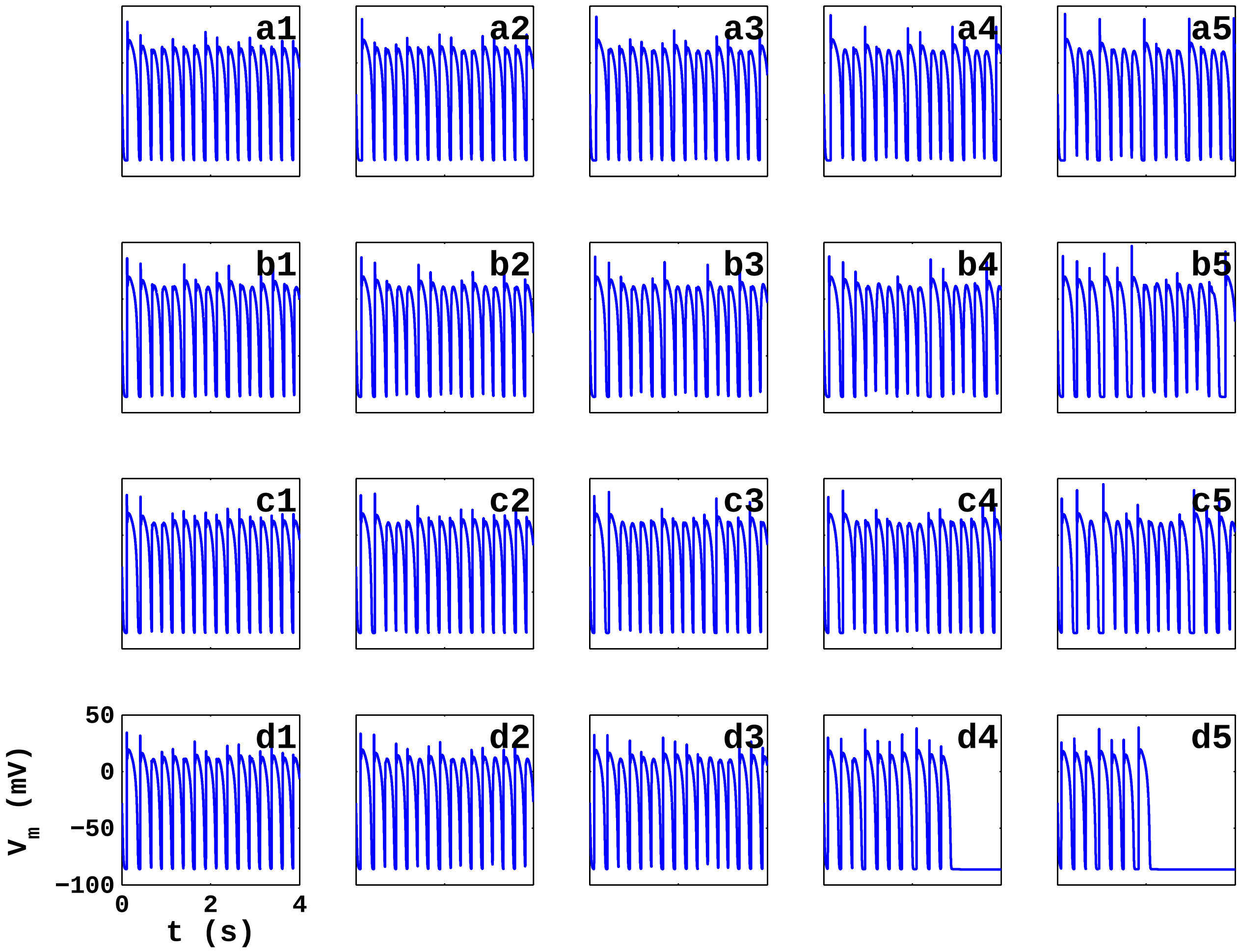

Figure S30. Plots of the local time series of $V_m(x, y, t)$, recorded from the representative points (x = 125 mm, y = 125 mm), in the TNNP04 model with the initial condition *IC2* and PD along both spatial directions; the spatiotemporal patterns of V_m are shown in Fig. S29.

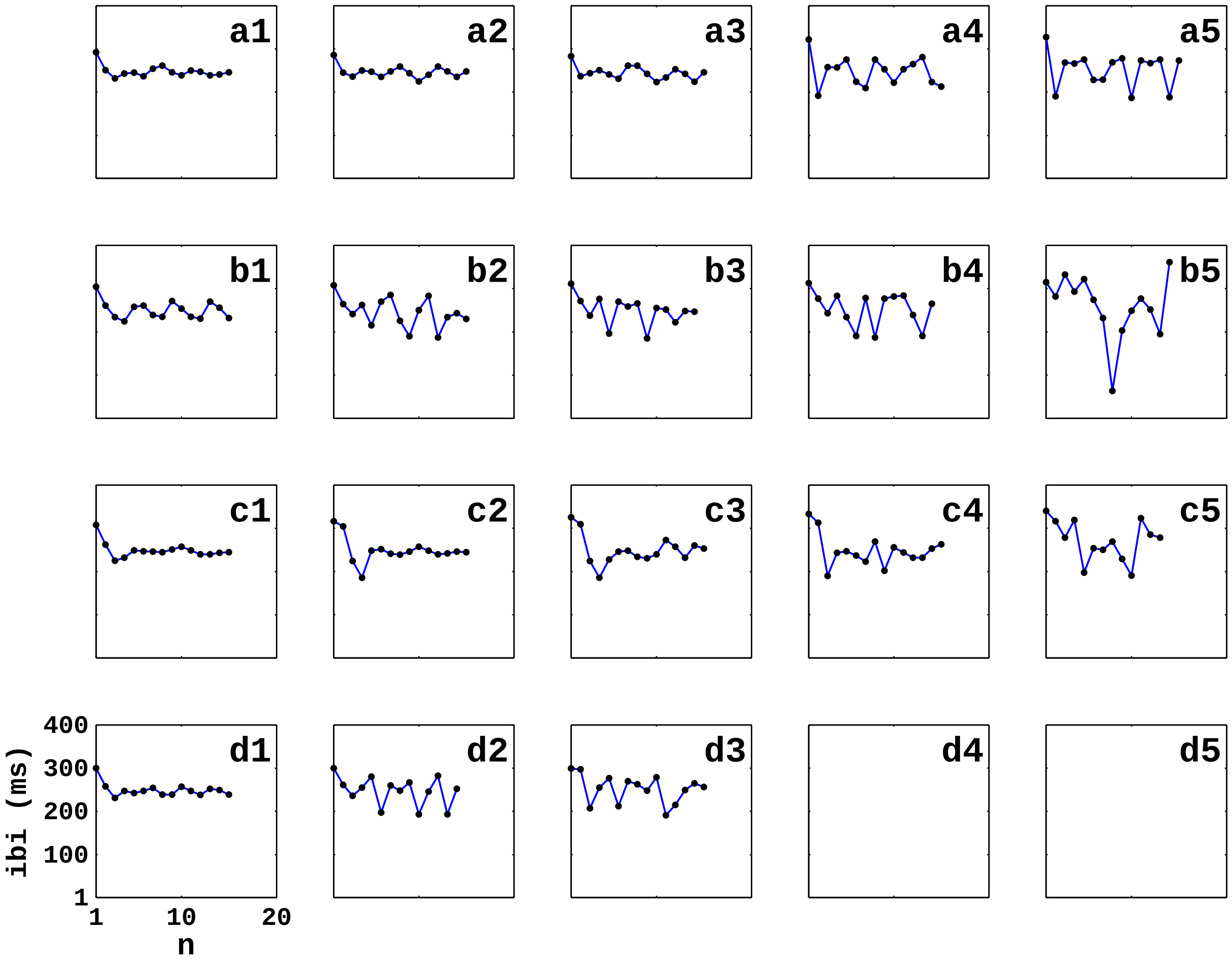

Figure S31. Plots of the ibi versus the beat number n that we obtain from the time series shown in Fig. S30.

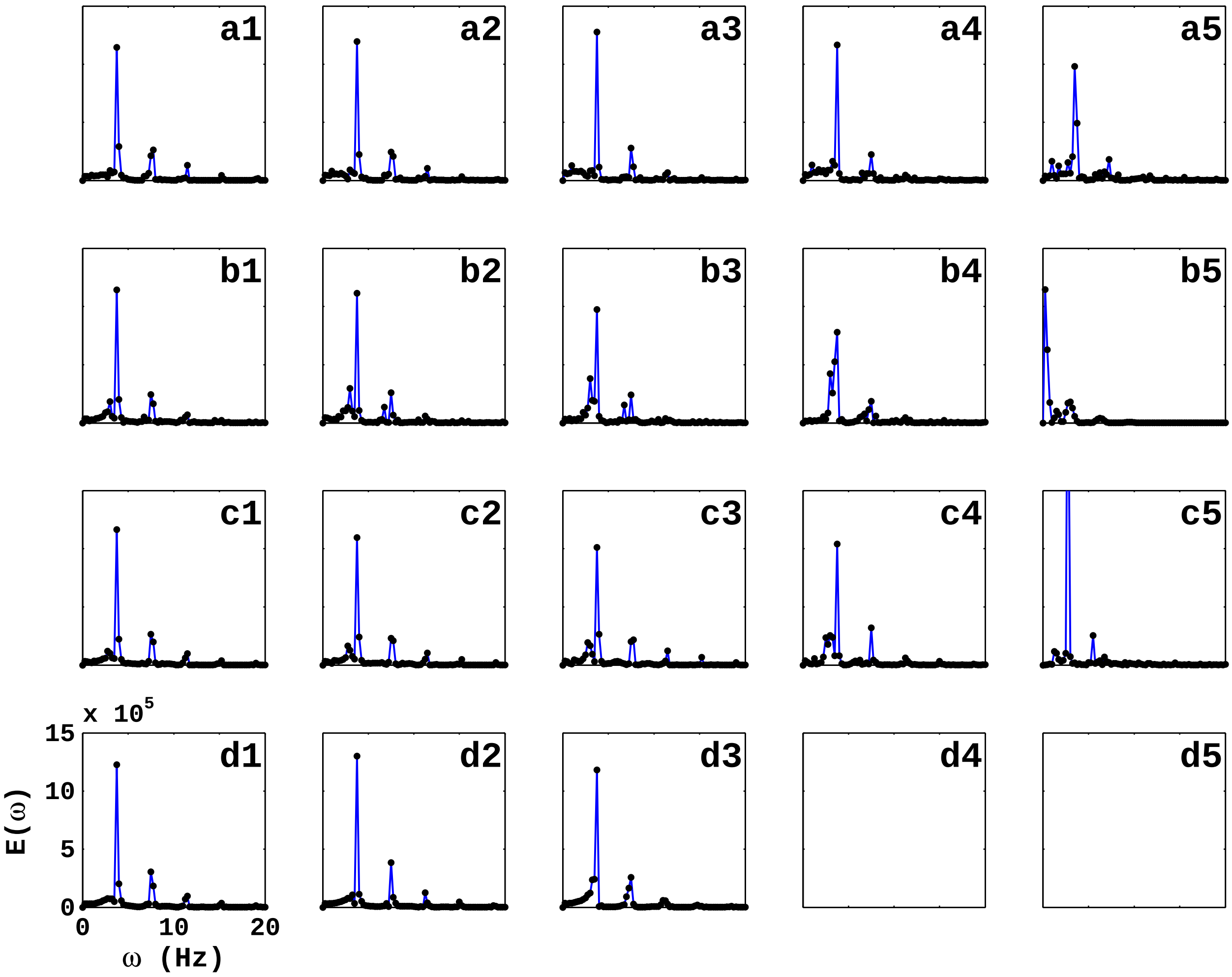

Figure S32. Plots of the power spectrum $E(\omega)$ of V_m obtained from a time series of length 2×10^5 for the data points in Fig. S30.

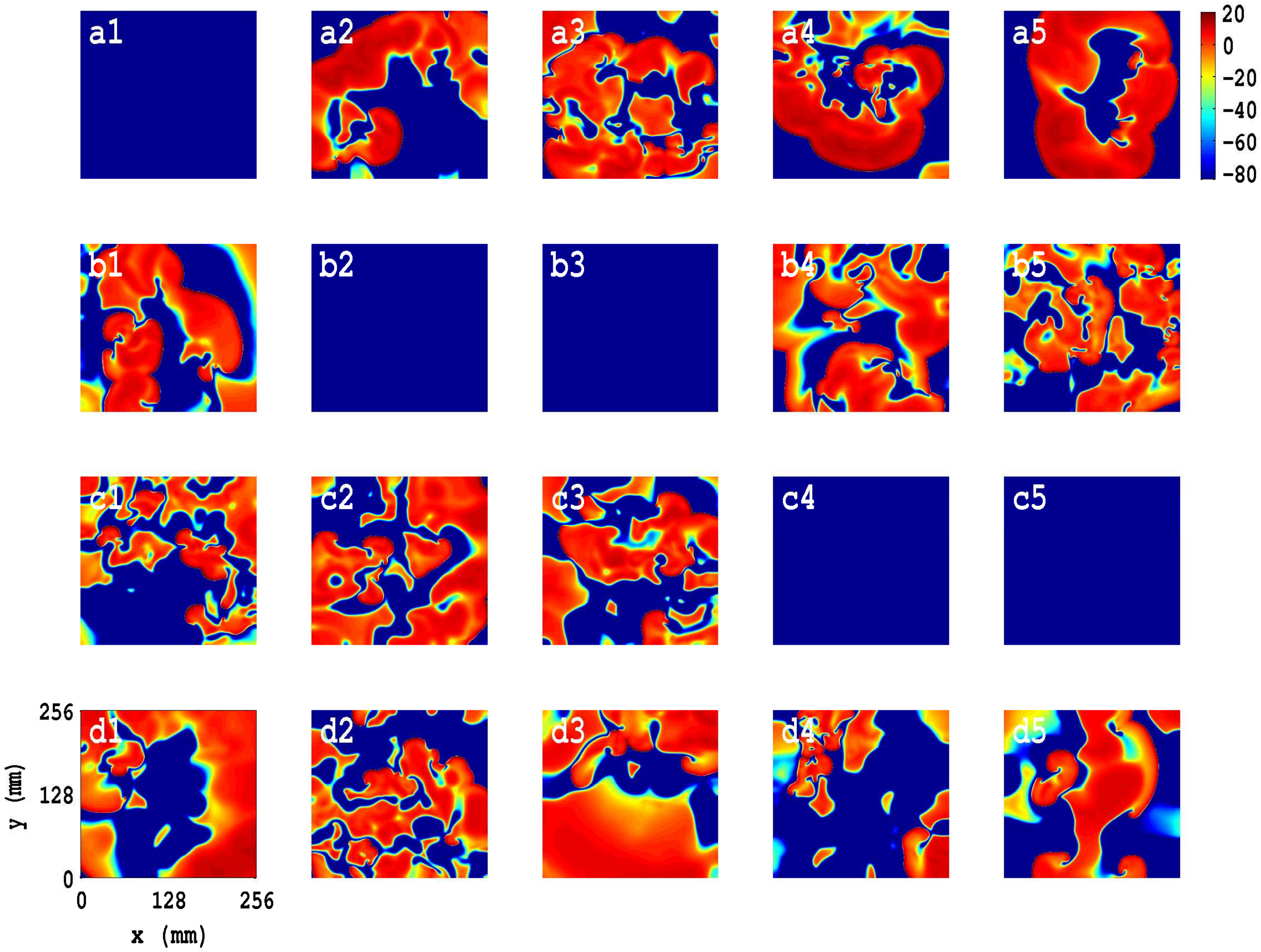

Figure S33. Illustrations of the rich variety of spatiotemporal patterns for the TNNP04 model, with PD along both x and y directions and the initial condition *IC3* (Fig. S6(i)): The analogs of the pseudocolor plots of V_m in Fig. S25 for the initial conditions *IC3*; the plots (a1)-(d5) here use the same PD parameters as their counterparts in Fig. S25; the Video S8 shows the spatiotemporal evolution of V_m for these cases for the time interval $0 \text{ s} \leq t \leq 4 \text{ s}$.

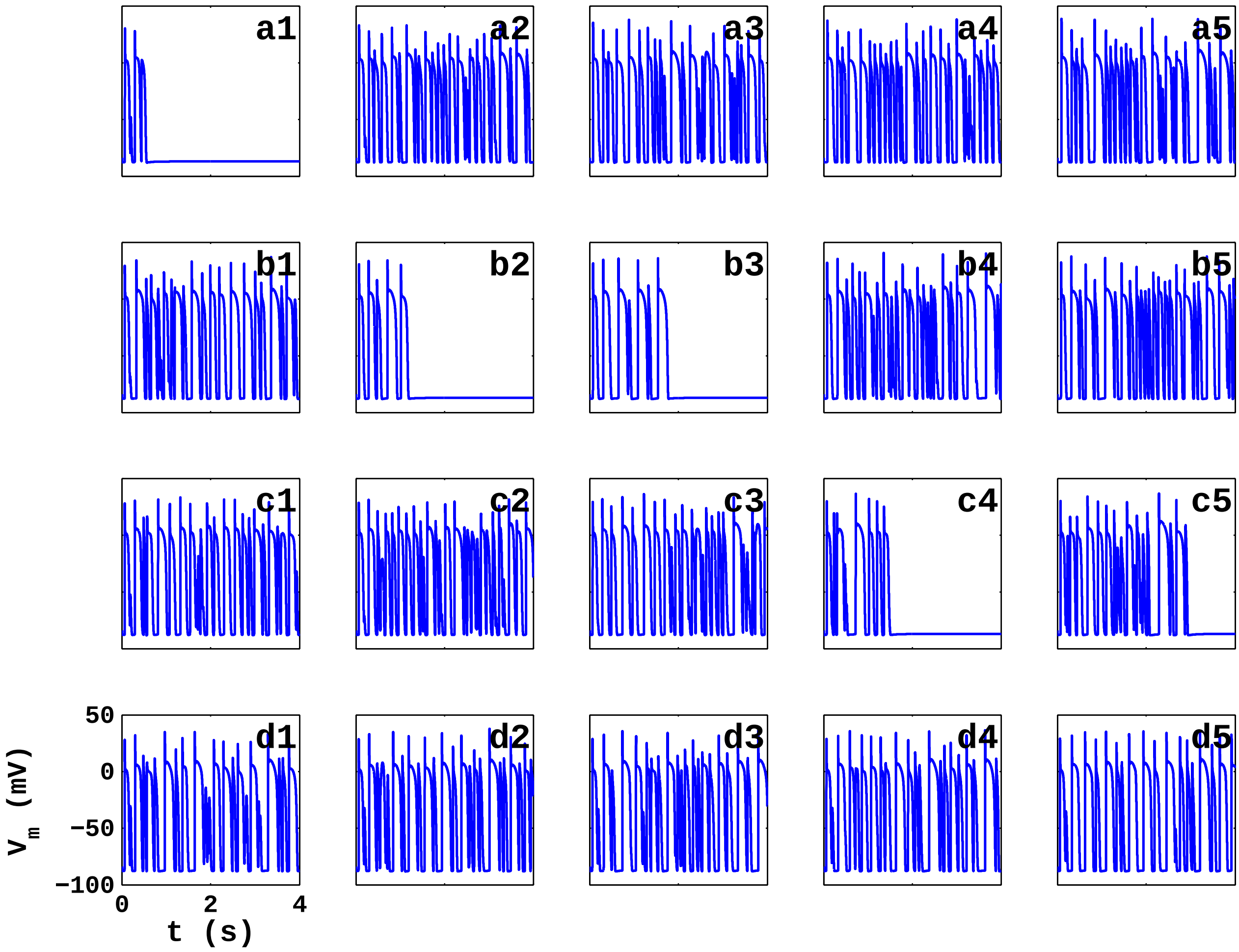

Figure S34. Plots of the local time series of $V_m(x, y, t)$, recorded from the representative points (x = 125 mm, y = 125 mm), in the TNNP04 model with the initial condition *IC3* and PD along both spatial directions; the spatiotemporal patterns of V_m are shown in Fig. S33.

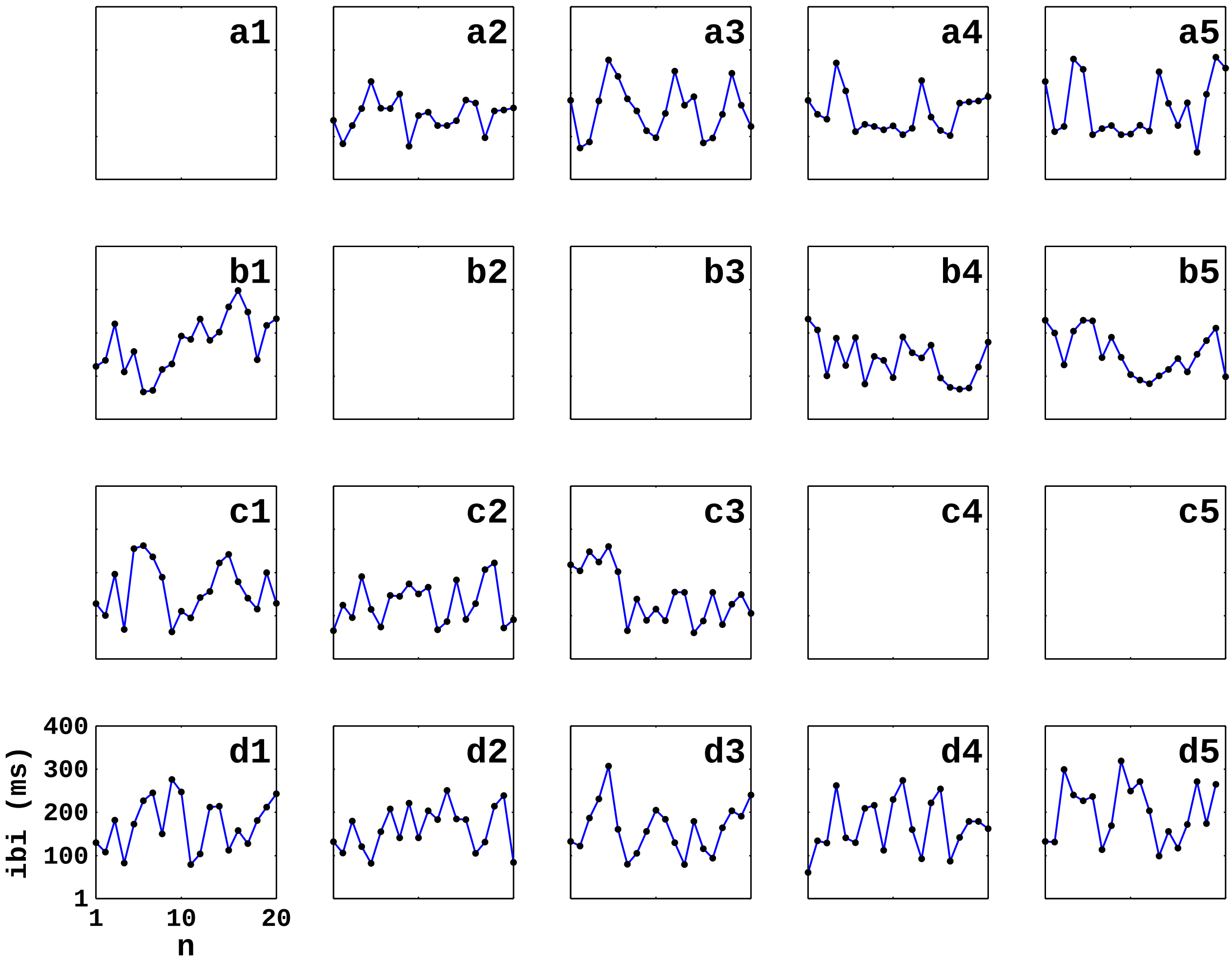

Figure S35. Plots of the ibi versus the beat number n that we obtain from the time series shown in Fig. S34.

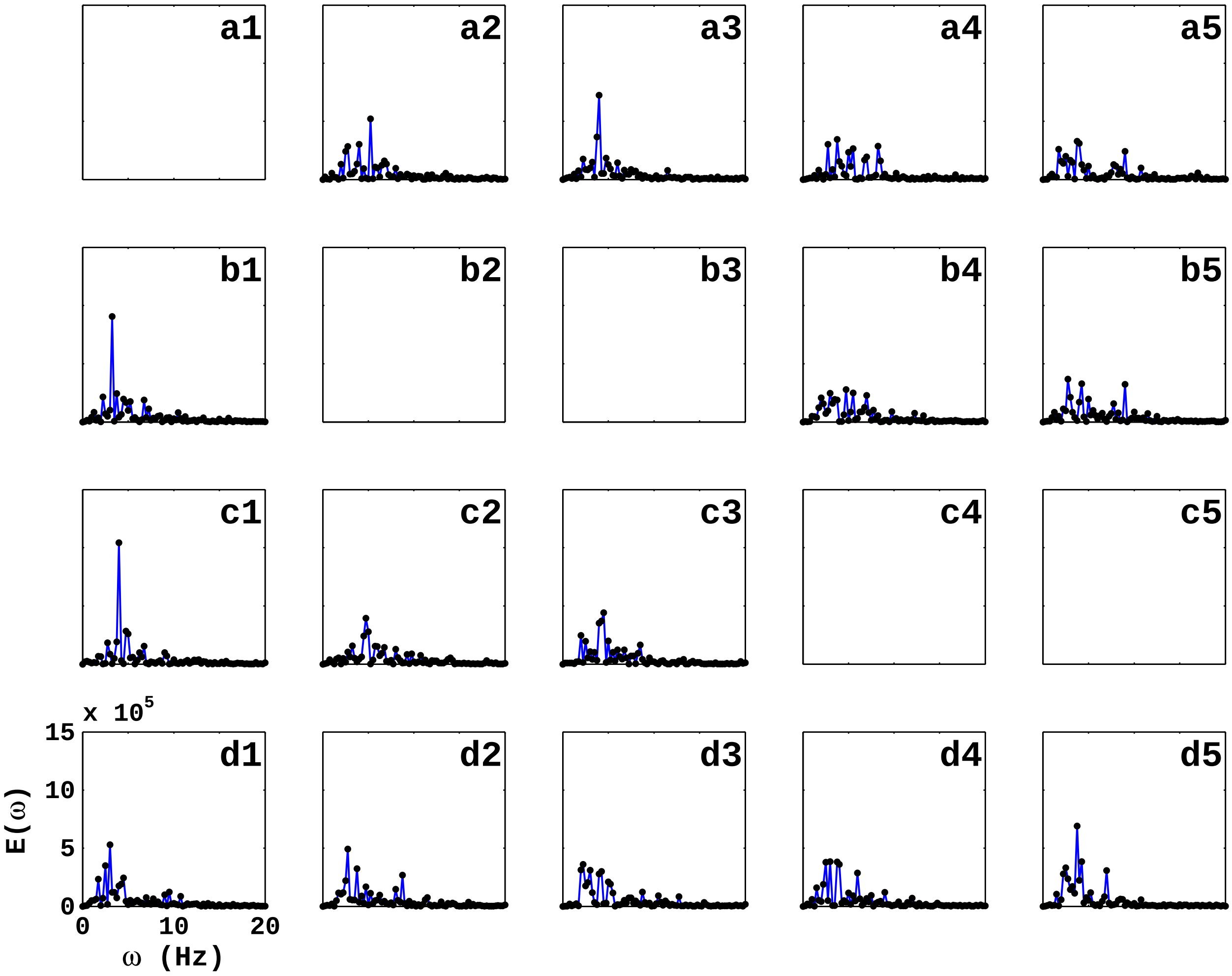

Figure S36. Plots of the power spectrum $E(\omega)$ of V_m obtained from a time series of length 2×10^5 for the data points in Fig. S34.

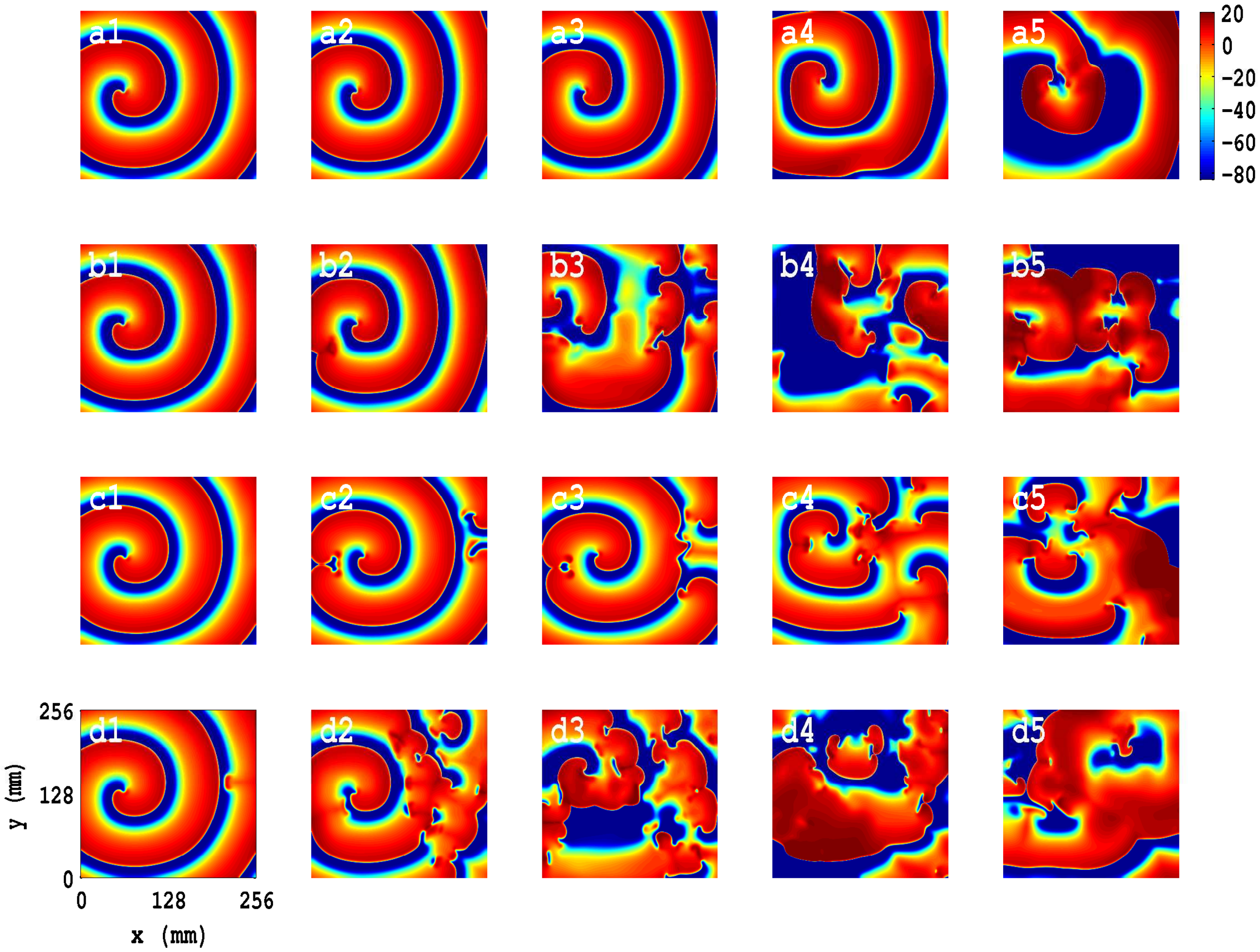

Figure S37. Illustrations of the rich variety of spatiotemporal patterns for the TP06 model, with PD along the x direction and the initial condition *IC1*: Representative pseudocolor plots of V_m with the initial condition *IC1* (Fig. S6(c)) and the parameter sets given in Table. 1 (main paper).

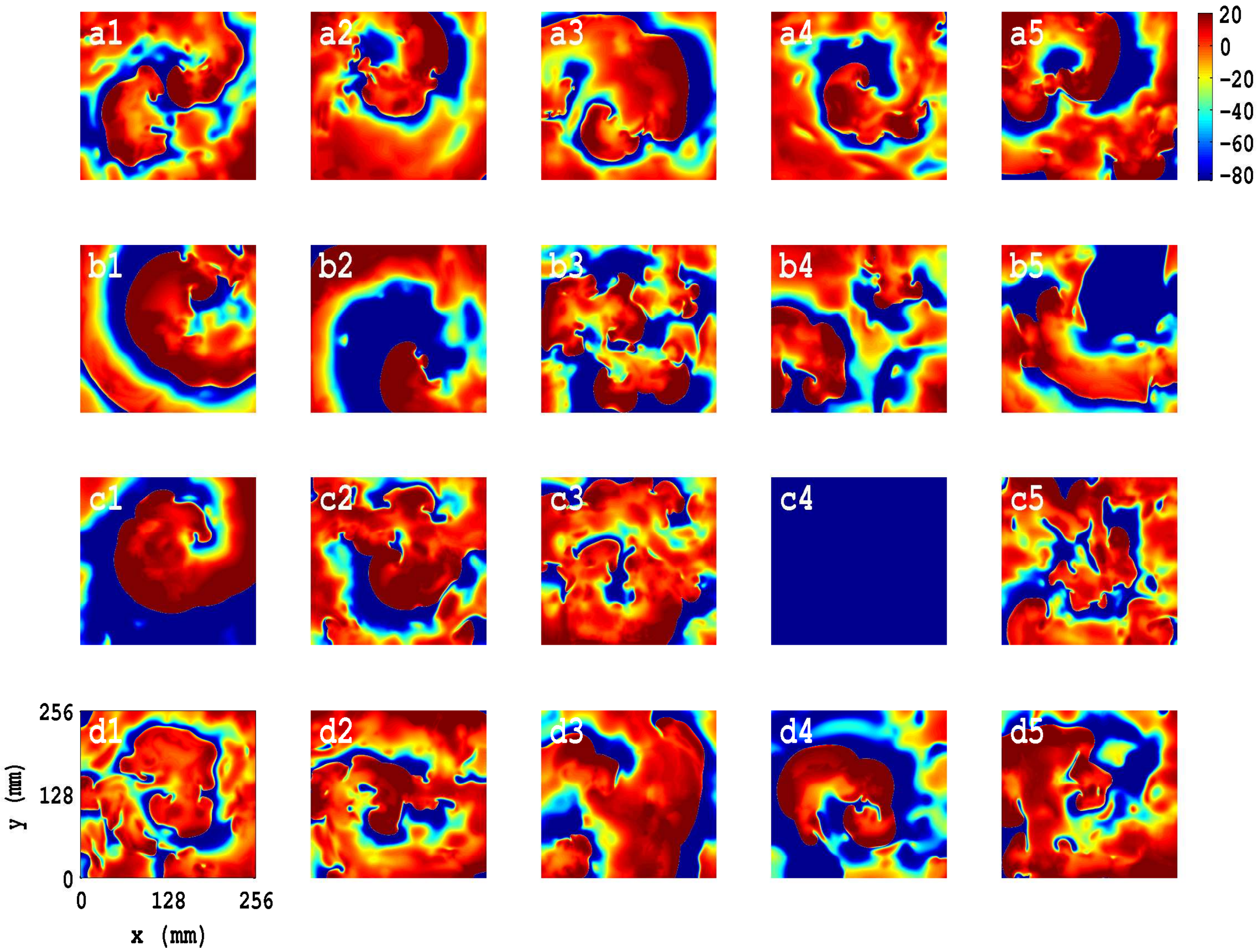

Figure S38. Illustrations of the rich variety of spatiotemporal patterns for the TP06 model, with PD along the x direction and the initial condition *IC2*: The analogs of the pseudocolor plots of V_m in Fig. S37 for the initial conditions *IC2*; the plots (a1)-(d5) here use the same PD parameters as their counterparts in Fig. S37.

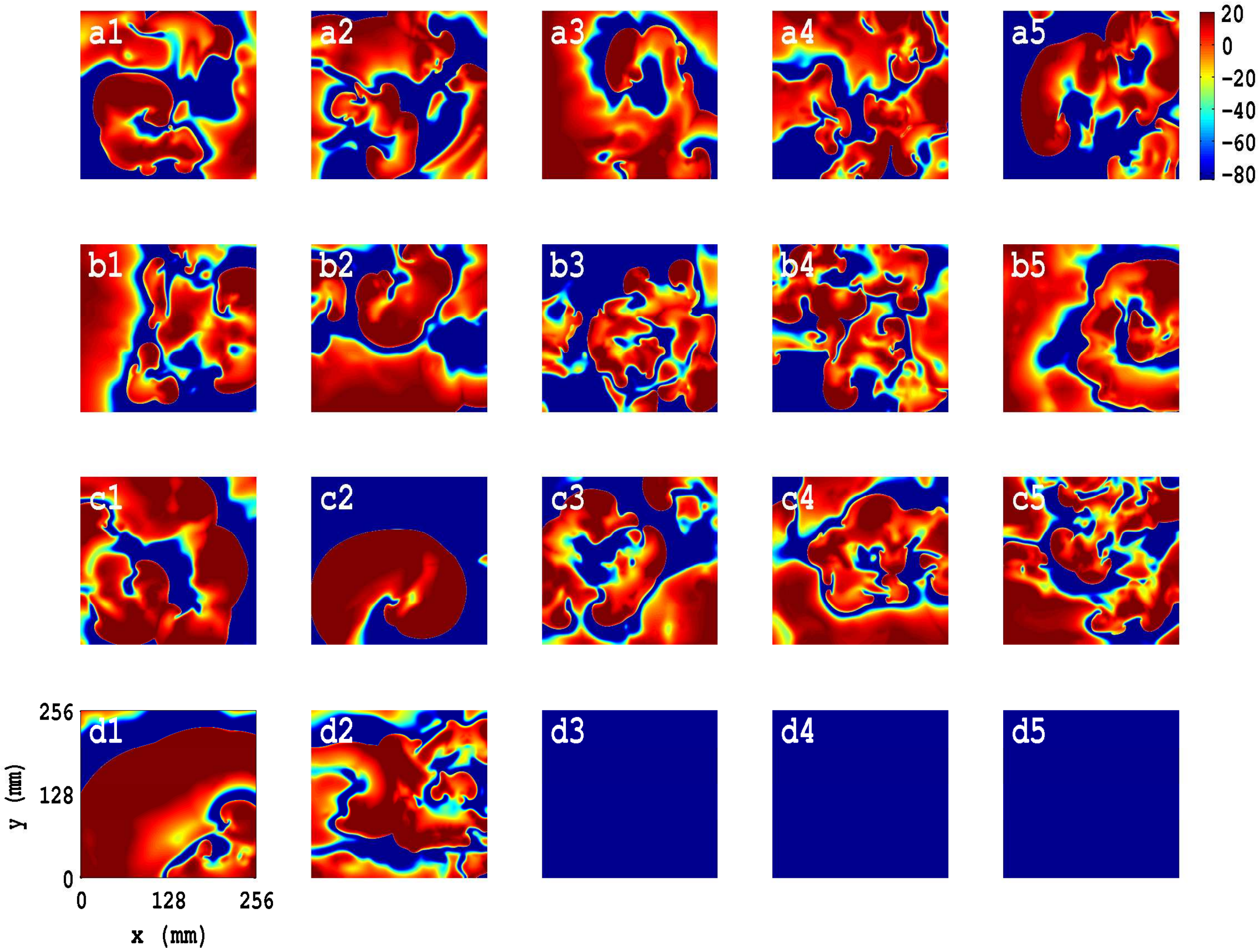

Figure S39. Illustrations of the rich variety of spatiotemporal patterns for the TP06 model, with PD along the x direction and the initial condition *IC3*: The analogs of the pseudocolor plots of V_m in Fig. S37 for the initial conditions *IC3*; the plots (a1)-(d5) here use the same PD parameters as their counterparts in Fig. S37.

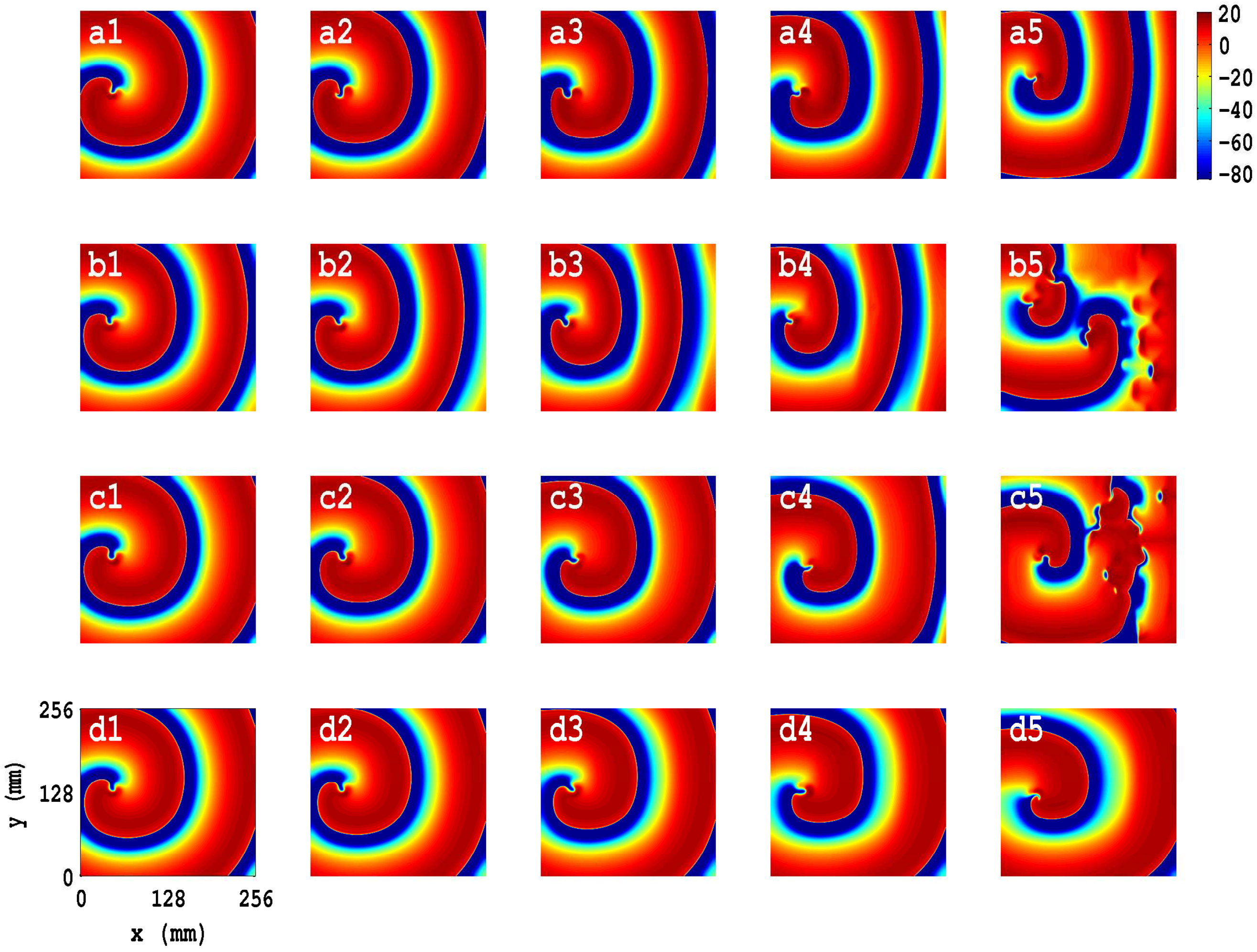

Figure S40. Illustrations of the rich variety of spatiotemporal patterns for the TNNP04 model, with PD along the x direction and the initial condition *IC1*: Representative pseudocolor plots of V_m with the initial condition *IC1* (Fig. S6(c)) and the parameter sets given in Table. 1 (main paper).

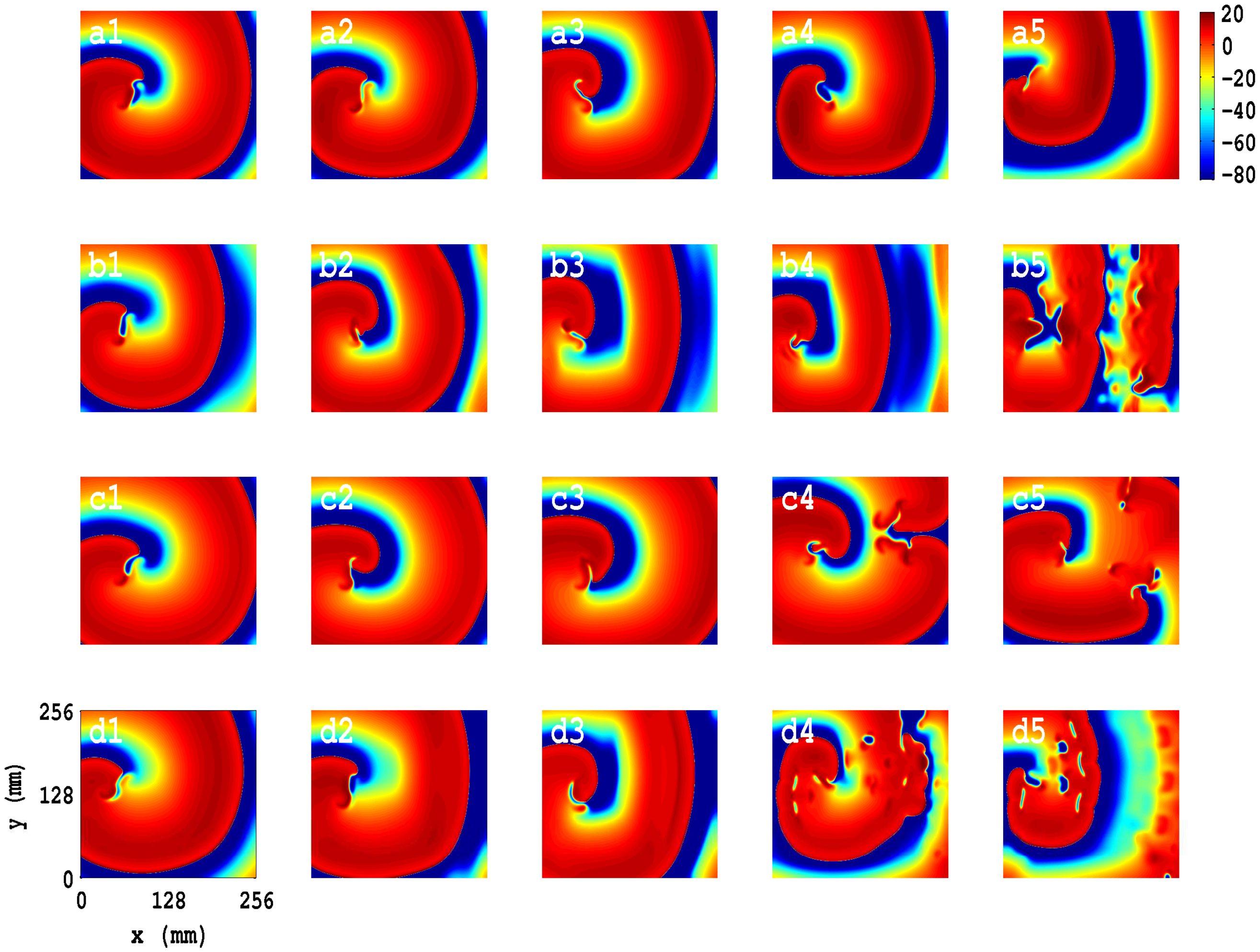

Figure S41. Illustrations of the rich variety of spatiotemporal patterns for the TNNP04 model, with PD along the x direction and the initial condition $IC2$: The analogs of the pseudocolor plots of V_m in Fig. S40 for the initial conditions $IC2$; the plots (a1)-(d5) here use the same PD parameters as their counterparts in Fig. S40.

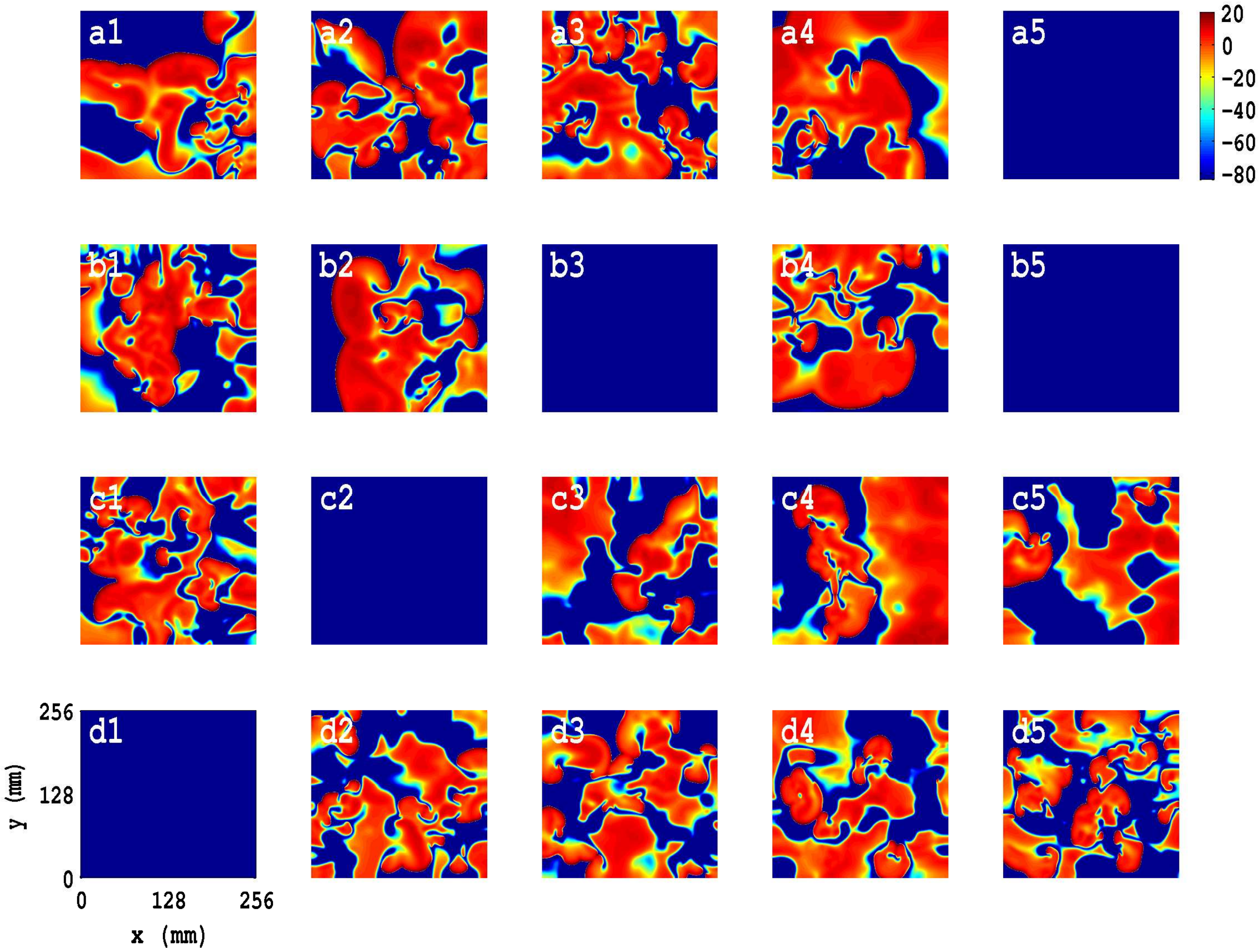

Figure S42. Illustrations of the rich variety of spatiotemporal patterns for the TNNP04 model, with PD along the x direction and the initial condition *IC3*: The analogs of the pseudocolor plots of V_m in Fig. S40 for the initial conditions *IC2*; the plots (a1)-(d5) here use the same PD parameters as their counterparts in Fig. S40.

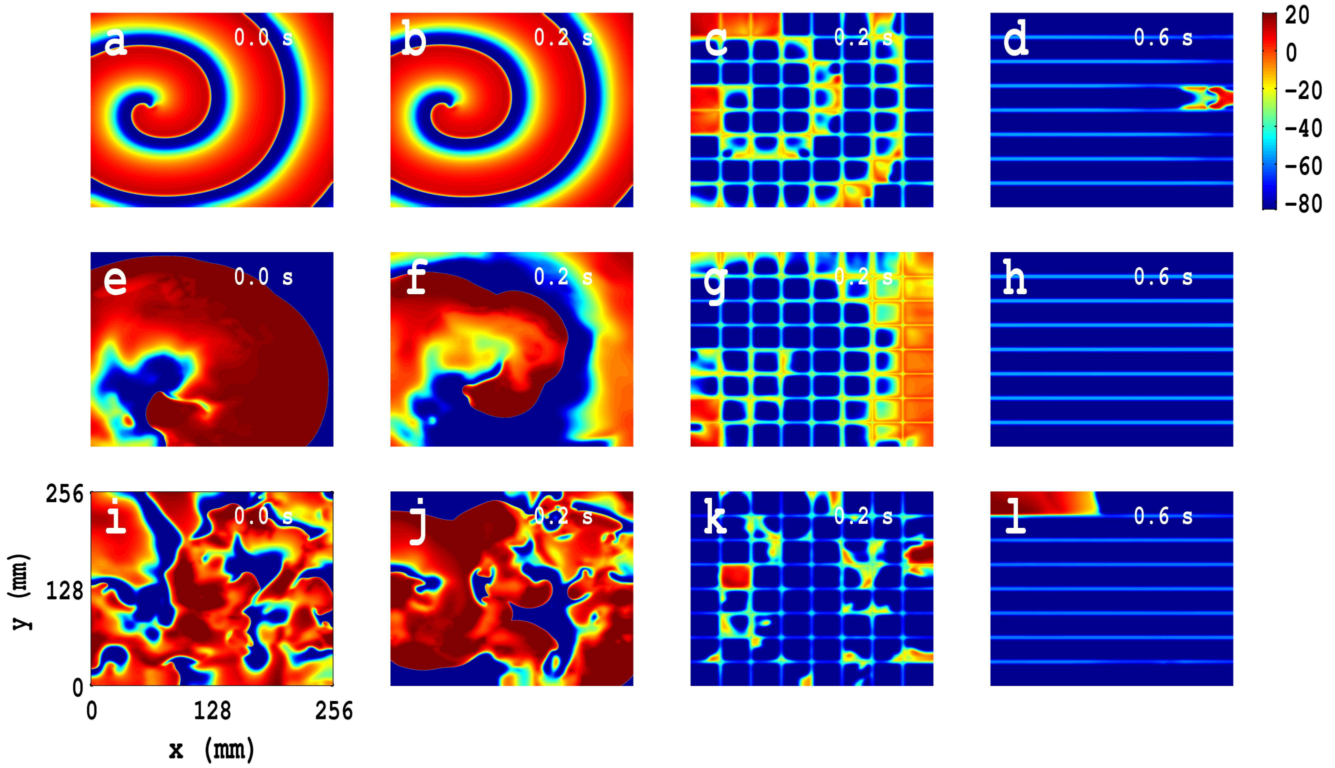

Figure S43. Spiral-wave suppression in the TP06 model, in the absence of PD, by low-amplitude pulses on square and line meshes: We illustrate spiral-wave suppression, via low-amplitude control pulses, in the TP06 model, in the absence of PD, by presenting pseudocolor plots of V_m. The spiral state, at time $t = 0$ s (a), with the $IC1$ initial condition, evolves, in the absence of the control, to an RS state (b), at time $t = 0.2$ s; this state is suppressed by the both square- and line-mesh suppression methods as shown in (c) and (d), at $t = 0.2$ s and $t = 0.6$ s, respectively. Similar plots for the $IC2$ and $IC3$ initial conditions are given, respectively, in (e)-(h) and (i)-(l); they show that an STSS and STMS states are suppressed by above square and line-mesh suppression algorithm. In all these cases we apply a control pulse of amplitude 75 pA/pF for $t = 0.2$ ms for the square mesh and an amplitude of 125 pA/pF for $t = 0.6$ ms for the line mesh.

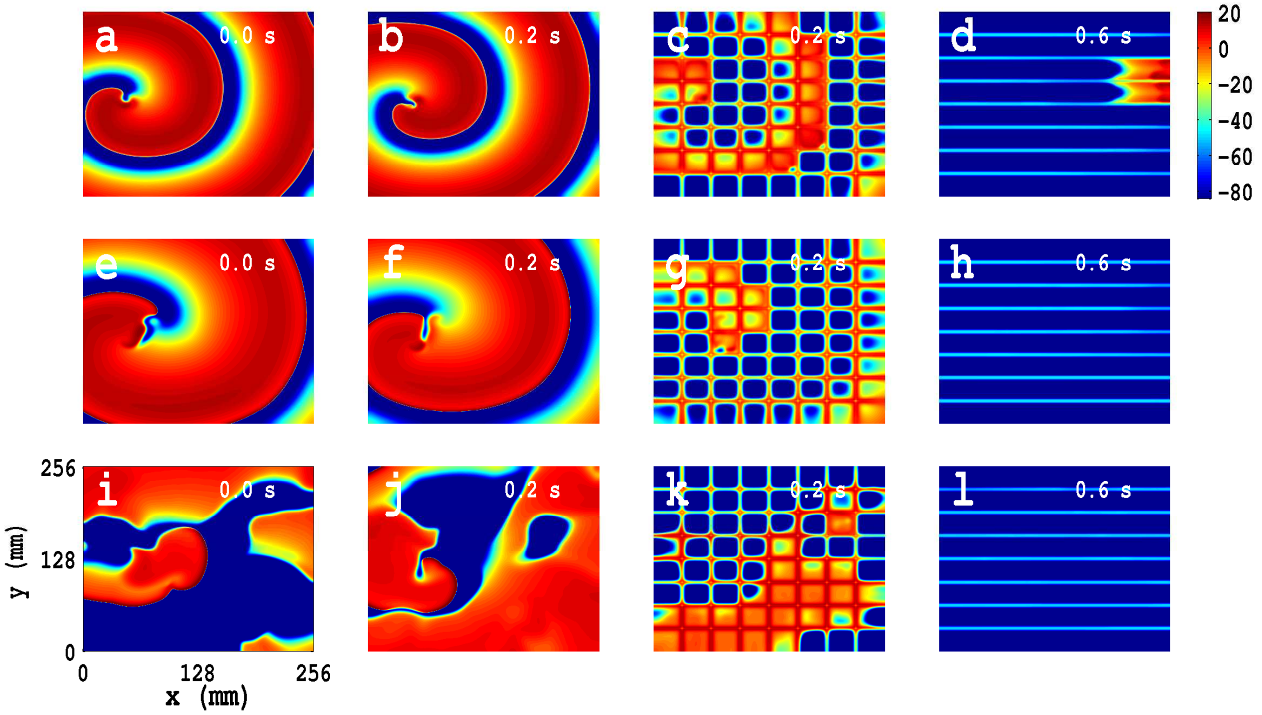

Figure S44. Spiral-wave suppression in the TNNP04 model, in the absence of PD, by low-amplitude pulses on square and line meshes: The analogs of the pseudocolor plots of V_m in Fig. S43. We illustrate an RS, RSZ, and STMS states are suppressed via low-amplitude control pulses, in the absence of PD, by presenting pseudocolor plots of V_m.

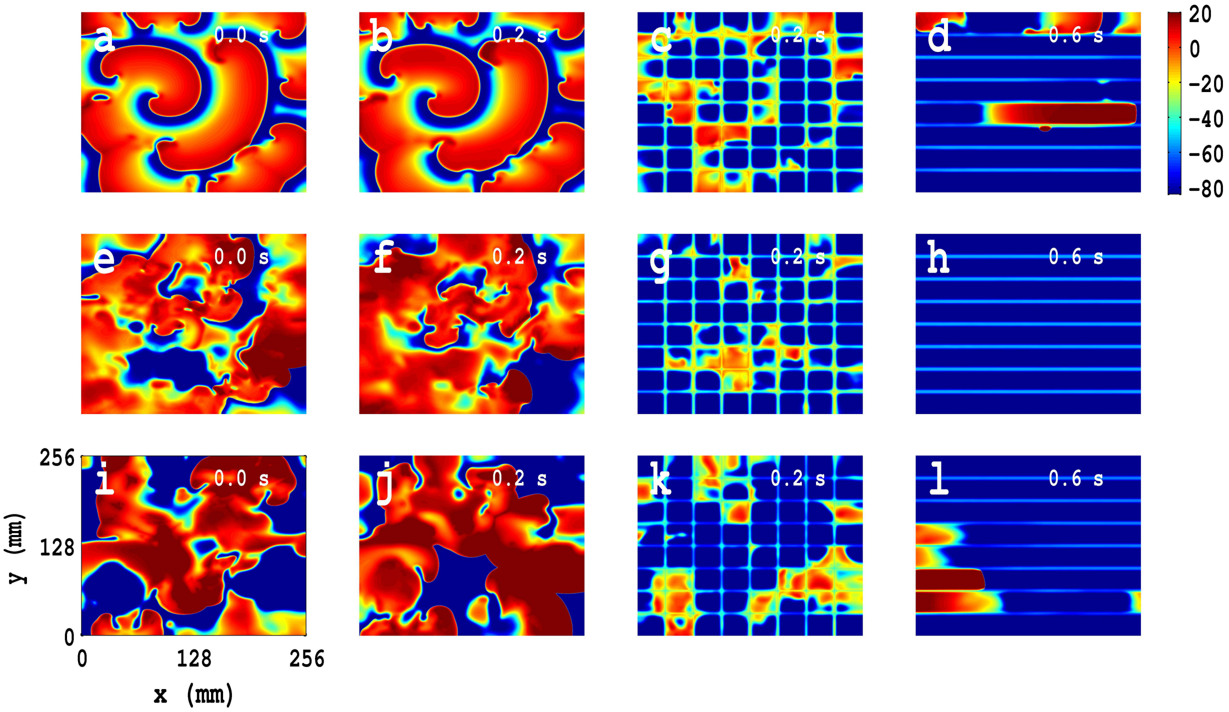

Figure S45. Spiral-wave suppression by low-amplitude pulses in the TP06 model with PD along only the x direction: We impose PD along the x direction and the illustrative amplitude $A_x = 0.3$ and frequeny $f_x = 5$ Hz; (a)-(l) are the analogs of Figs. S43(a)-(l), respectively. With the initial condition *IC1*, the spiral in (a), at $t = 0$ s, evolves, in the absence of control, to an MST state (b), at $t = 0.2$ s; this MST can be suppressed by both square- and line-mesh suppression (c) and (d) at $t = 0.2$ s and 0.6 s, respectively. For the *IC2* and *IC3* initial configurations, the analogs of these states are shown in (e)-(h) and (i)-(l), respectively; clearly, both our suppression schemes are successful in eliminating spiral turbulence with PD along one direction.

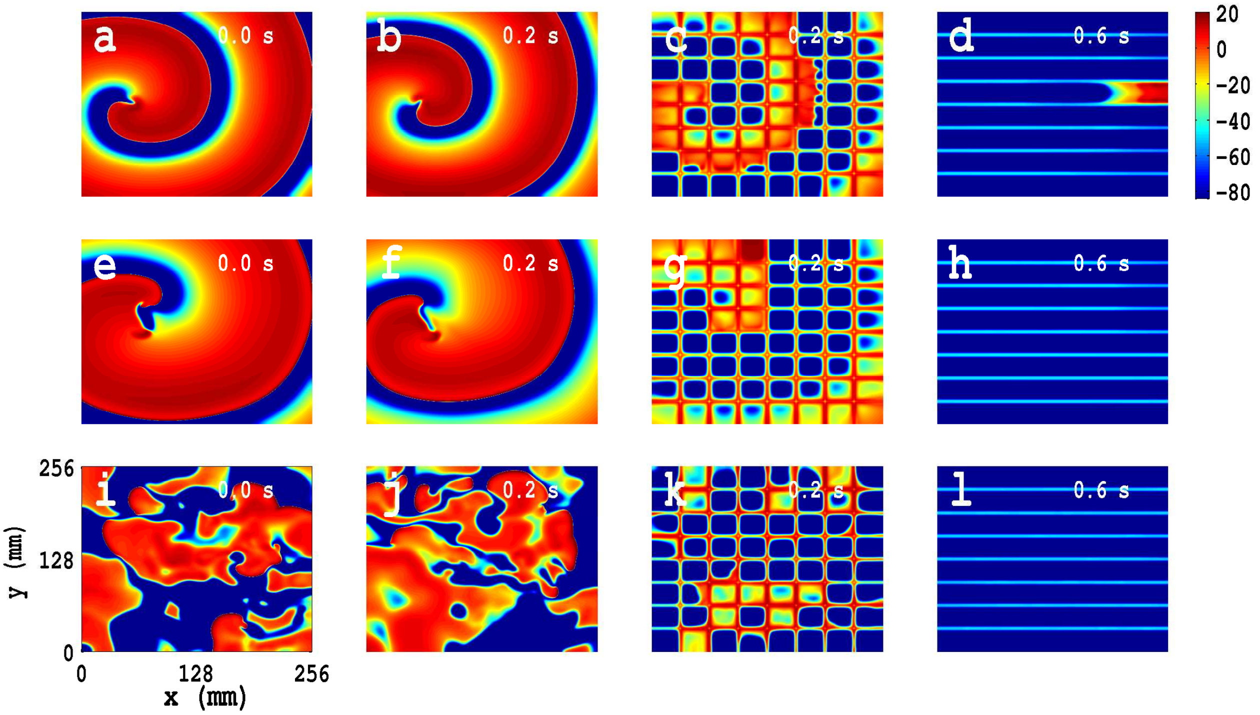

Figure S46. Spiral-wave suppression by low-amplitude pulses in the TNNP04 model with PD along only the x direction: We impose PD along the x direction and the illustrative amplitude $A_x = 0.3$ and frequency $f_x = 5$ Hz; (a)-(l) are the analogs of Figs. S43(a)-(l), respectively. With the initial condition *IC1*, the spiral in (a), at $t = 0$ s, evolves, in the absence of control, to an MST state (b), at $t = 0.2$ s; this MST can be suppressed by both square- and line-mesh suppression (c) and (d) at $t = 0.2$ s and 0.6 s, respectively. For the *IC2* and *IC3* initial configurations, the analogs of these states are shown in (e)-(h) and (i)-(l), respectively; clearly, both our suppression schemes are successful in eliminating spiral turbulence with PD along one direction.

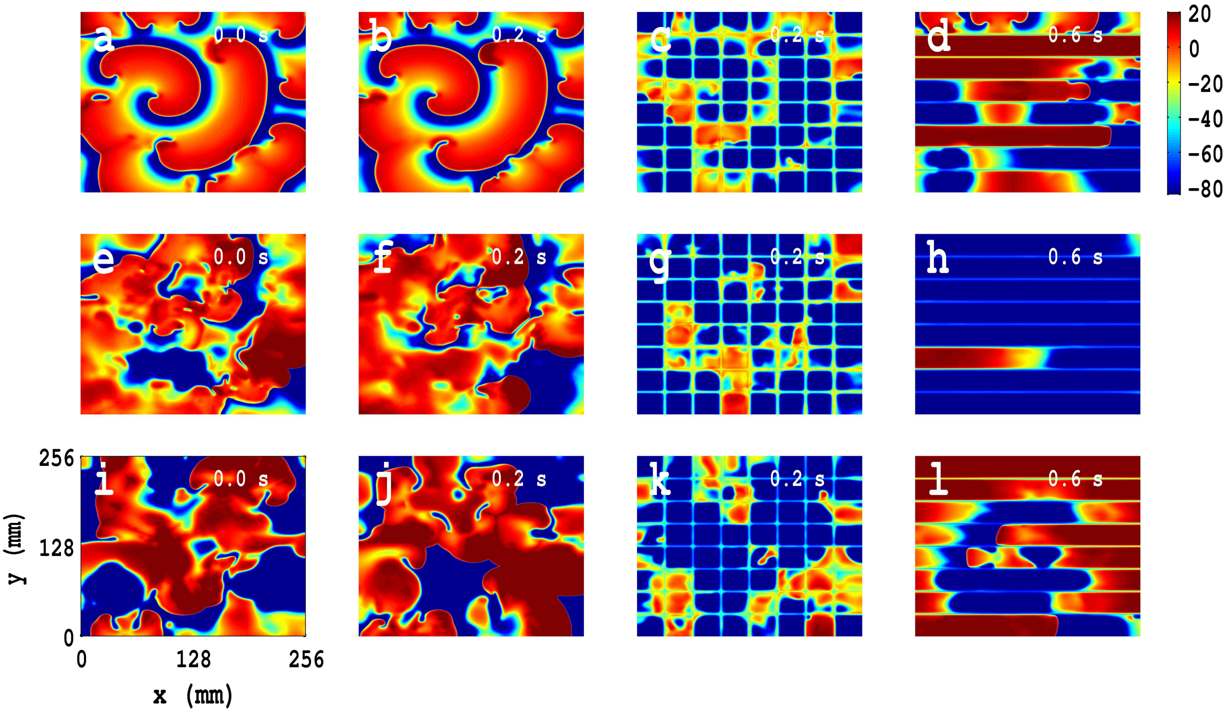

Figure S47. Spiral-wave suppression by low-amplitude pulses in the TP06 model with PD along both x and y directions: We impose PD along both x and y directions with the illustrative amplitudes $A_x = A_y = 0.3$ and frequencies $f_x = f_y = 5$ Hz; for this case (a)-(l) are the analogs of Figs. S43 (a)-(l), respectively. With the initial condition *IC1*, the spiral in (a), at $t = 0$ s, evolves, in the absence of the control, to the MST state in (b), at $t = 0.2$ s; this MST can be suppressed by the square-mesh technique but not by the line-mesh technique as we show in (c) and (d) at $t = 0.2$ s and 0.6 s, respectively; the parameters on the control mesh are as in Fig. S46. For the initial conditions *IC2* and *IC3*, the analogs of these states are shown, respectively, in (e)-(h) and (i)-(l). Thus, with PD along both directions, spiral turbulence can be suppressed by our square-mesh control but not the line-mesh method for $IC1$ and $IC3$ initial conditions.

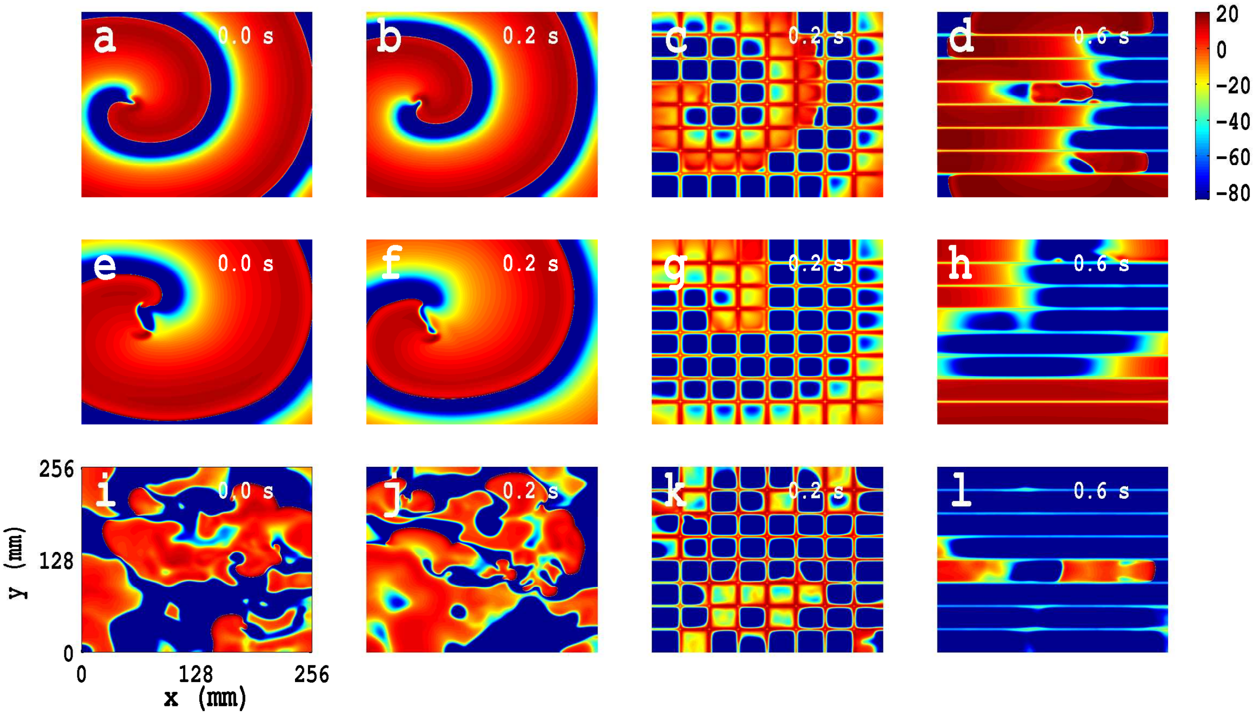

Figure S48. Spiral-wave suppression by low-amplitude pulses in the TNNP04 model with PD along both x and y directions: We impose PD along both x and y directions with the illustrative amplitudes $A_x = A_y = 0.3$ and frequencies $f_x = f_y = 5$ Hz; for this case (a)-(l) are the analogs of Figs. S43 (a)-(l), respectively. With the initial condition *IC1*, the spiral in (a), at $t = 0$ s, evolves, in the absence of the control, to the MST state in (b), at $t = 0.2$ s; this MST can be suppressed by the square-mesh technique but not by the line-mesh technique as we show in (c) and (d) at $t = 0.2$ s and 0.6 s, respectively; the parameters on the control mesh are as in Fig. S46. For the initial conditions *IC2* and *IC3*, the analogs of these states are shown, respectively, in (e)-(h) and (i)-(l). Thus, with PD along both directions, spiral turbulence can be suppressed by our square-mesh control but not the line-mesh method for $IC1$ and $IC3$ initial conditions.

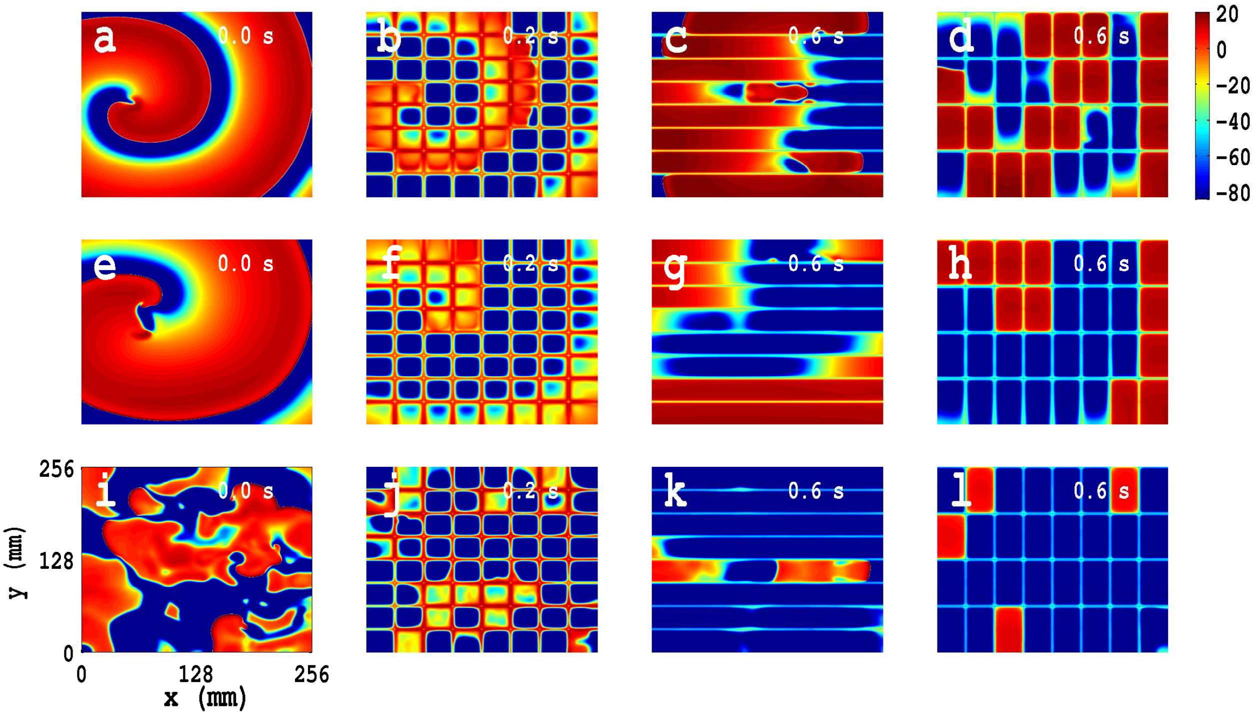

Figure S49. Comparison of spiral-wave suppression by low-amplitude pulses on square, line, and rectangular control meshes in the TNNP04 model, with PD along both x and y directions: We impose PD along both x and y directions with the illustrative amplitudes $A_x = A_y = 0.3$ and frequencies $f_x = f_y = 5$ Hz for the initial configurations *IC1*, *IC2*, and *IC3* (pseudocolor plots of V_m in (a), (e), and (f), respectively). We apply the following control pulses: amplitude 75 pA/pF for $t = 0.2$ s over a square mesh ((b), (f), and (j)), with each square block of side $l = 32$ mm; amplitude 125 pA/pF for $t = 0.6$ s over a line mesh ((c), (g), and (k)), with inter-line spacing $l = 32$ mm; amplitude 125 pA/pF for $t = 0.6$ s over a rectangular mesh ((d), (h), and (l)), with block sides $l_x = 32$ mm and $l_y = 64$ mm. These pseudocolor plots of V_m show that this spiral states, with *IC1*, *IC2*, and *IC3* initial conditions, are suppressed by both square- and rectangular-mesh suppression but not line-mesh suppression.
